# Is the effect of person-organisation fit on turnover intention mediated by job satisfaction? A survey of community health workers in China

**DOI:** 10.1136/bmjopen-2016-013872

**Published:** 2017-02-20

**Authors:** Mingji Zhang, Fei Yan, Wei Wang, Guohong Li

**Affiliations:** 1 School of Public Health, Shanghai Jiaotong University, Shanghai, China; 2 School of Public Health, Fudan University, Shanghai, China; 3 Key Laboratory of Health Technology Assessment (Ministry of Health), Collaborative Innovation Center of Social Risks Governance in Health, Shanghai, China

**Keywords:** Person organization fit, work attitude, community health workers

## Abstract

**Objectives:**

Person-organisation fit (P-O fit) is a predictor of work attitude. However, in the area of human resource for health, the literature of P-O fit is quite limited. It is unclear whether P-O fit directly or indirectly affects turnover intention. This study aims to examine the mediation effect of job satisfaction on the relationship between P-O fit and turnover intention based on data from China.

**Design and methods:**

This is a cross-sectional survey of community health workers (CHWs) in China in 2013. A questionnaire of P-O fit, job satisfaction and turnover intention was developed, and its validity and reliability were assessed. Multiple regression and structural equation modelling were used to examine the relationship among P-O fit, job satisfaction and turnover intention.

**Setting and participants:**

Multistage sampling was applied. In total, 656 valid questionnaire responses were collected from CHWs in four provincial regions in China, namely Shanghai, Shaanxi, Shandong and Anhui.

**Results:**

P-O fit was directly related to job satisfaction (standardised β 0.246) and inversely related to turnover intention (standardised β −0.186). In the mediation model, the total effect of P-O fit on turnover intention was −0.186 (p<0.001); the direct effect of P-O fit on turnover intention was −0.094 (p<0.01); the indirect effect of job satisfaction on the relationship between P-O fit and turnover intention was −0.092 (p<0.001).

**Conclusions:**

The effect of P-O fit on turnover intention was partially mediated through job satisfaction. It is suggested that more work attitude variables and different dimensions of P-O fit be taken into account to examine the complete mechanism of person-organisation interaction. Indirect measures of P-O fit should be encouraged in practice to enhance work attitudes of health workers.

**Strengths and limitations of this study**

- The questionnaire has good reliability and validity, so the results were trustworthy.
- This was the first person-organisation fit study on health workers in China at a national level.
- We did not use any established questionnaire to make this study more comparable.
- The number of attitudinal variables were limited, which may explain why the total effect and direct effect were both small.
- Data collected could not be representative of the whole population of community health workers in China. Nevertheless, through careful analysis methods, the findings of this study have external validity for theory verification.

## Introduction

Individuals make continuous effort to maintain a correspondence with the work environment, the process of which is called work adjustment.^1^ One result from this process is person-organisation fit (P-O fit), which is defined as “the compatibility between people and organisations that occurs when: (1) at least one entity provides what the other needs, or (2) they share similar fundamental characteristics, or (3) both”.^2^ P-O fit theory assumes that employees' work attitudes and behaviour are not caused by organisational or individual factors separately, but by the extent of P-O fit.^3^

There are several ways to measure P-O fit. Direct measurement (sometimes called subjective measurement) assesses fit by asking respondents to report their perceptions of individual correspondence with their organisation, whereas perceived indirect measurement compares separate assessments of the respondents' perceptions of individual characteristics and the characteristics of their organisations.^4^ Objective indirect fit asks the respondent to describe his/her own personal characteristics but then asks someone else to describe the organisational characteristics; the fit measure is constructed from personal and organisational characteristics.^5^

Researchers found four dimensions of P-O fit, namely value congruence, goal congruence, personality-climate congruence and needs-supplies congruence.^5^ Value congruence is the degree of fit between organisational and individual values.^6^ Researchers also use goal congruence, the degree to which organisational members agree on the priorities of organisational goals, to operationalise P-O fit.^7^ The third dimension describes P-O fit as a match between the characteristics of individual personality and organisational climate.^2^ From the needs-supplies perspective, P-O fit occurs when the organisation satisfies an individual's needs, desires or preferences.^2^ Meta-analysis reviews found that the majority of P-O fit studies were about value congruence.^5^ Few studies have been conducted using a dimension other than the value congruence framework,^8^ such as the needs-supplies dimension.

In the area of human resources for health, P-O fit literature was quite limited. One study on nurses found that value congruence influenced job satisfaction and their attitude towards wards.^9^ Another study was about nurses-supervisor value congruence, which is only a part of P-O fit.^10^ Until now, P-O fit of needs-supplies dimension and its relationship with job satisfaction and turnover intention have not been fully tested in the area of human resources for health.

In 2009, China embarked on a new round of health reform which emphasised reinforcing community health services (CHS).^11^ The role of CHS is to provide primary healthcare for residents within its catchment, focusing mostly on the elderly, patients with non-communicable diseases, pregnant women and children. Community health workers (CHWs) provide basic medical treatments but, more importantly, promote health through health education, health management, disease screening, etc. Briefly, basic public health services and basic medical services comprise functions of CHS. Thus, CHS reform policies propelled the forming of a new profession of (CHWs, most of whom were medical staff in primary level hospitals before the reform but are now providers of a combination of basic public health services and basic medical care. After several years' reform, work adjustment between CHWs and their work environment gradually settled down. Given the latest policy reform in China and the emergence of the CHW occupation, the relationship between P-O fit in the needs-supplies dimension, job satisfaction and turnover intention should be investigated to prevent job turnover and short-staffing.

### Hypothesis

P-O fit has a considerable effect on attitudinal outcomes, for example, intent to quit, job satisfaction and organisational commitment.^5^ When individual and organisational values showed poor fit, there were reduced job satisfaction and increased intentions to turnover.^12^ In the light of these previous findings, we proposed the first and second hypotheses about P-O fit from the needs-supplies dimension (we would simply use ‘P-O fit’ from now on), job satisfaction and turnover intention.

Hypothesis 1: Job satisfaction is positively related to P-O fit. Job satisfaction will increase when P-O fit increases.

Hypothesis 2: Turnover intention is negatively related to P-O fit. As P-O fit increases, turnover intention will decrease.

When employees perceived a decreased job satisfaction, they would become more inclined to consider leaving their job. Many studies conclude that job satisfaction is a stable predictor of turnover intention^13^ and turnover behaviour.^14^ Though P-O fit and job satisfaction are both predictors of turnover intention, whether interaction exists among the three variables has not been explored in the area of human resource for health.

What is widely acknowledged is that job satisfaction is determined by organisational resources as well as by the expectation of employees.^15^ ^16^ Accordingly, we can reasonably assume that P-O fit was the main cause of job satisfaction. Furthermore, P-O fit is an antecedent of work attitude, a conceptual structure, but not a variable of work attitude per se. In other words, individuals cannot directly feel P-O fit. Thus, the effect of P-O fit on turnover intention must be delivered through some work attitude variable, such as job satisfaction. Consequently, we state the third hypothesis.

Hypothesis 3: The effect of P-O fit on turnover intention is fully mediated by job satisfaction. When job satisfaction is controlled, P-O fit will no longer be significantly correlated with turnover intention.

## Methods

### Design

This study used data from ‘the Survey of Human Resources for Community Health Services’, one commissioned project of the 5th National Health Service Survey of China. The survey, conducted in 2013, investigated the quantity and structure of human resources in CHS, as well as P-O fit, job satisfaction and turnover intention of CHWs. The latter part of the investigation was what this study draws on.

Before the survey, investigators were trained about how to use the instruments and interview the participants. During the survey, all responses to the questionnaire were guided by investigators. Also, supervisors and managers of CHS were not present in the field to allow CHWs give genuine answers. Consequently, the quality of data collection was guaranteed, and bias during the survey was minimalised.

### Samples

The study applied multistage sampling. First, in a widely accepted way, provincial regions of China were stratified into developed area (East China), less-developed area (Middle China) and the least developed area (West China). Then, on the basis of performance in CHS reform, we purposively sampled four typical provincial regions (three provinces and one municipality; for convenience, they will all be addressed as ‘province’ afterwards), two from East China (Shanghai and Shandong), the other two from Middle China (Anhui) and West China (Shaanxi), respectively. Second, one or two urban districts from every province were also purposively selected according to their different performance in reform, and a total of seven districts were sampled. Third, CHS were sampled by their ownership (eg, private, collective or state-owned), and all staff of sampled CHS who were at work that day were surveyed. A total of 713 CHWs participated in face-to-face interviews, but a few pieces of the questionnaire were not finished because CHWs were called to work. Finally, 656 valid responses were collected. For those 656 pieces of questionnaire, missing data were filled according to the mean of their colleagues. All respondents were informed about investigation arrangements, and they provided written or verbal informed consent before being interviewed.

### Instruments

The variables of interest were P-O fit, job satisfaction and turnover intention. The possible confounders were location, gender, age and professional title among others. To acquire these data, we developed a P-O fit questionnaire for this study. The questionnaire included a scale to assess needs of CHWs and organisational supplies, measures for general job satisfaction and turnover intention, and questions for demographic information, for example, province, gender, age and professional title.

### Job satisfaction and turnover intention

A single-item was used to measure general job satisfaction by asking ‘To what extent do you think you are satisfied with your work’? Meta-analysis concluded that this measure for job satisfaction is acceptable, because it had decent reliability, brevity, face validity and sensitivity to detect changes in job satisfaction^17^ and also stronger correlation with turnover intention.^13^ Meta-analysis also proved validity of single-item measure, because it found that a single-item measure had significant positive correlation with the multiple-item job satisfaction measure (r=0.82).^18^ Responses were measured on a five-point Likert scale that was ranged in this way: 1=‘very dissatisfied’, 2=‘dissatisfied’, 3=‘no clear inclination’, 4=‘satisfied’ and 5=‘very satisfied.’ Although a multiitem scale of job satisfaction could represent the structure of facets of job satisfaction, the reliability and validity of both a multiitem scale and single-item measure are both acceptable. As long as only the overall job satisfaction is concerned, we could tolerate the neglect of structure of job satisfaction.

We also used a single-item measure for turnover intention by asking ‘How often do you think about leaving your job recently?’ Single-item turnover measure was first used by Spector (1985)^19^ and had demonstrated narrow or unambiguous construct.^20^ A five-point Likert scale was used, where 1=‘never consider to leave the job’, 2=‘seldom consider to leave the job’, 3=‘sometimes consider to leave the job’, 4=‘often consider to leave the job’ and 5=‘have thought about quitting all the time’.

### P-O fit scale

Results from meta-analysis indicated that subjective measures had weaker correlation with behavioural outcomes than perceived or objective fit measures.^8^ Also, direct measurement would probably increase common method bias.^8^ Therefore, we chose a perceived indirect approach for P-O fit measurement, that is, we acquired respondents' ratings on individual needs (P score) and organisation supplies (O score), respectively; then P-O fit was obtained by calculating P and O scores.^5^

Warr (2011)^21^ analysed 12 aspects of job features that were believed to represent the needs of employees. On the basis of expert consultation, we identified nine items from Warr's inventory and three other items of individual needs. The scale was constructed based on Alderfer's Existence, Relatedness and Growth (ERG) theory. Existence needs items include working condition, income level, benefits package, workload and job security. Relatedness needs items were coworker relationship, respect from community and participation in decision-making. Growth needs consist of career development, learning opportunity, honour and management. Participants were asked to rate the importance of the need items from 1 to 10, with ‘1’ indicating ‘the least important’ and ‘10’ indicating ‘the most important’. P-O fit scores were converted from the 10-point scale to a five-point scale to be consistent with the job satisfaction and turnover intention measures. Perceived organisation supplies used the same items as individual needs to guarantee a consistent measure between needs and supplies.^22^

After a pilot study of the questionnaire in three CHS in Shanghai, exploratory factor analysis revealed that the factor structure of P-O fit diverged from the three factor structure in the ERG theory. Only two factors of relatedness and growth were found, and three items were not included in this structure. Growth factor was later renamed ‘achievement’ to better represent the factor. Reliability and validity of this instrument were also examined (the P-O fit scale and its reliability and validity are shown in Results section). The aforementioned changes were made to adapt the questionnaire.

### Analysis methods

Calculation methods of the P-O fit index vary. However, the most commonly used method is difference score, which subtracts the total need score from the total supply score. This study also used difference score, because it keeps most of the original information of respondents' ratings.^5^ ^22^ To test theoretical assumptions with new empirical evidence of P-O fit in our areas of human resources for health, it is also reasonable to adopt the commonly used measure of P-O fit. A P-O fit score greater than zero indicates a positive person-organisation interaction, where the CHW's needs are fulfilled by the organisation, whereas a negative P-O fit score indicates that the organisation does not fulfil the individual's needs.

Multiple regression model was used in SPSS V.21. Two multiple regression models were tested. In model 1, P-O fit was used as an independent variable and JS as the dependent variable. In model 2, P-O fit was the independent variable of interest, while turnover intention was the dependent variable. Structural equation modelling was used in AMOS V.21.0, including confirmatory factor analysis (CFA), and path analysis. CFA was used to test reliability and validity of the P-O fit scale. Path analysis was applied to further test mediation among general job satisfaction, turnover intention and P-O fit, while controlling for confounders such as province, gender, age and professional title.

## Results

### Instrument validity and reliability

**Figure1:**
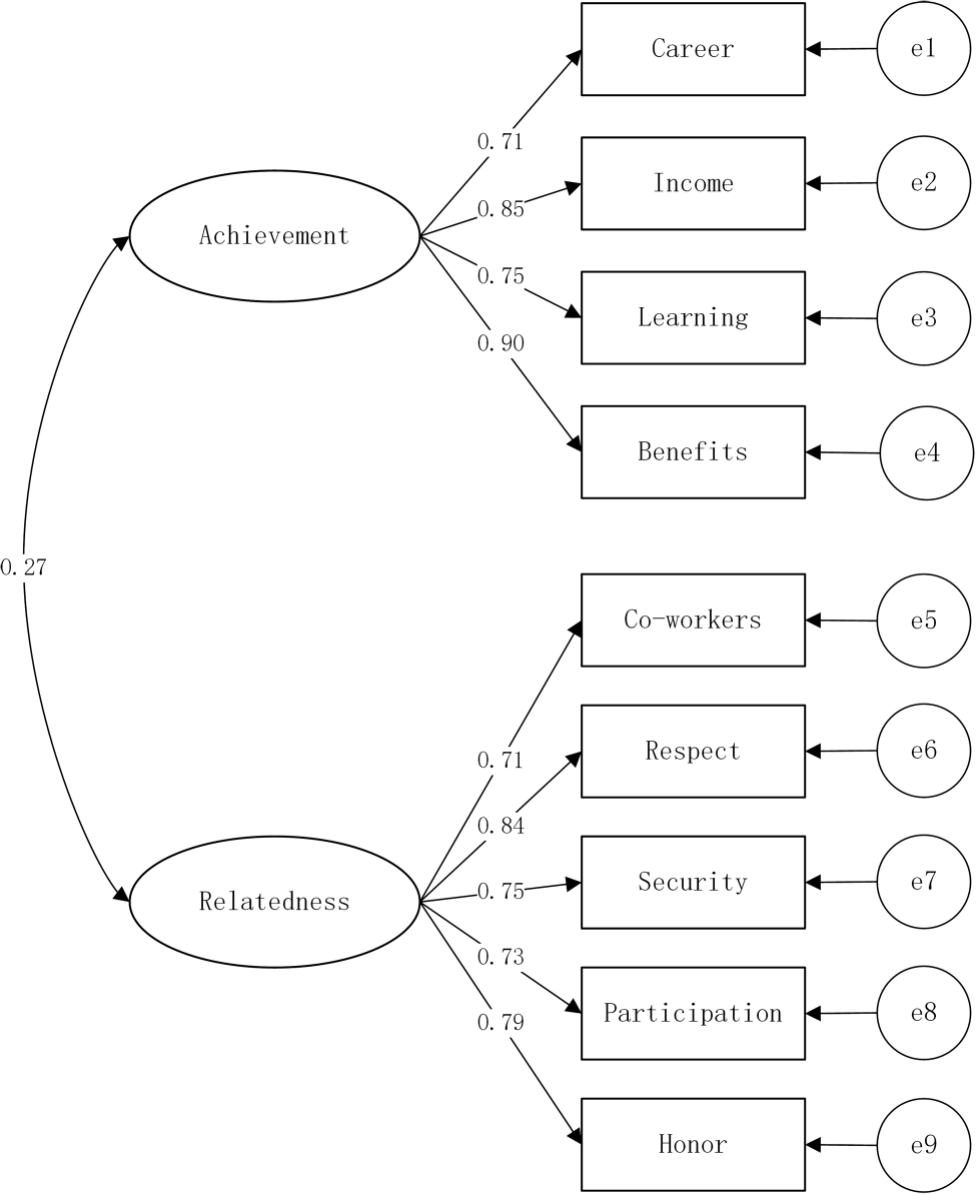
Confirmatory factor analysis of community health workers' (CHWs') needs structure of person-organisation fit. This figure shows that the structure of CHWs' needs are constituted of the achievement factor and the relatedness factor. e1 to e9 are error terms, while a rectangle icon is an observed variable and a circle icon is a latent variable. A single headed arrow shows a regression path from an independent variable to a dependent variable, while a double-headed arrow shows the covariance between the two variables.

We examined reliability and validity of the P-O fit scale. Cronbach's α was 0.830, suggesting a decent internal consistency. In CFA, we found that the nine items were significantly related to Relatedness or Achievement factor, as is shown in figure 1. Composite reliability for the Achievement factor was 0.88, and 0.87 for Relatedness, suggesting a good composite reliability.^23^ The average variance extracted (AVE) for Achievement was 0.65, 0.59 for Relatedness, and both were larger than 0.5, showing a good convergent validity.^23^ The two AVE statistics were also larger than the covariance of two factors, meeting the requirement for discriminant validity.^24^

Fit indices indicated that this scale had a good fit: CMINDF=4.21, RMSEA=0.07, GFI=0.97, CFI=0.976, and TLI=0.961. In sum, our P-O fit scale demonstrated acceptable reliability and validity for our study sample. The P-O fit scale, its response means and SDs are presented in table 1.

**Table 1:** Items of person-organisation fit scale (a five-point Likert scale)

	Means	SD
Needs: to what extent do you think the following items can motivate you to work harder?		
A good income	3.96	1.10
A good benefit package	3.74	1.22
Smooth career development	3.73	1.11
Rich learning opportunities	3.69	1.03
Participation in decision-making	3.3	1.10
Honour given by the organisation	3.41	1.11
Respect from the community	3.85	0.93
Good relationship with colleagues	3.95	0.95
Job security (a stable job)	3.66	1.12
Supplies: to what extent do you think your organisation provides the following items?		
A good income	2.67	0.79
A good benefit package	3.03	0.88
Smooth career development	2.78	0.90
Rich learning opportunities	3.33	0.78
Participation in decision-making	3.33	1.05
Honour given by the organisation	3.28	0.74
Respect from the community	3.8	0.69
Good relationship with colleagues	4.27	0.64
Job security (a stable job)	3.64	0.77

### Descriptive statistics

Demographic information of the study sample is represented in table 2. Across all sites, the majority of CHWs were female (about 80%), under the age of 40 years (about 69%) and junior-title professionals (except in Shanghai). CHWs professions were distributed differently in cities, especially in Shanghai where all doctors of clinical medicine were transferred to be general practitioners through a training and certification programme as required by the authority. The other cities were in the middle of the same course. The raw data of this study can be accessed in online supplementary files.

**Table 2:** Demographic information of study sample of community health workers in China, 2013

	Anhui (N=173)		Shandong (N=233)		Shaanxi (N=154)		Shanghai (N=96)		Total (N=656)	
	N	Per cent	N	Per cent	N	Per cent	N	Per cent	N	Per cent
Gender										
Female	119	68.8	200	85.8	124	80.5	79	82.3	522	79.6
Male	54	31.2	33	14.2	30	19.5	17	17.7	134	20.4
Age (years)										
<29	64	37.0	100	42.9	39	25.3	14	14.6	217	33.1
30–39	61	35.3	65	27.9	63	40.9	45	46.9	234	35.7
40–49	37	21.4	36	15.5	40	26.0	35	36.5	148	22.6
50–59	9	5.2	22	9.4	12	7.8	2	2.1	45	6.9
>60	2	1.2	10	4.3	0	0.0	0	0.0	12	1.8
Professional title										
No title	19	11.0	34	14.6	16	10.4	7	7.3	76	11.6
Junior	116	67.1	126	54.1	97	63.0	33	34.4	372	56.7
Intermediate	34	19.7	66	28.3	37	24.0	55	57.3	192	29.3
Senior	4	2.3	7	3.0	4	2.6	1	1.0	16	2.4
Professions										
Doctors of clinical medicine	18	10.4	22	9.4	9	5.8	0	0.0	49	7.5
General practitioners	35	20.2	37	15.9	29	18.8	39	40.6	140	21.3
Doctors of traditional medicine	12	6.9	13	5.6	8	5.2	1	1.0	34	5.2
Doctors of public health	8	4.6	18	7.7	11	7.1	15	15.6	52	7.9
Nurses	69	39.9	88	37.8	56	36.4	39	40.6	252	38.4
Medical laboratory technicians	31	17.9	55	23.6	41	26.6	2	2.1	129	19.7

10.1136/bmjopen-2016-013872.supp1 supplementary file

Table 3 exhibited mean scores of three main indicators of interest. CHWs had a job satisfaction score of 3.55 (SD=0.74), which means they were somewhere between ‘no clear inclination’ and ‘satisfied’ with their work. About 52.8% of CHWs reported that they were satisfied with their work (rating 4 or 5). The average turnover intention was below 3.00, indicating a low tendency to quit their job. Overall, 13.1% CHWs had a quite clear turnover intention (ie, rating 4 or 5). As to P-O fit, the mean P-O fit score was −0.35 (SD=0.78), which indicates a gap in the needs of CHWs and the resources provided by the organisation.

**Table 3:** Means, SDs and correlations of indicators of community health workers in China, 2013

	Mean	SD	1	2	3
1 General job satisfaction	3.55	0.74	1.00		
2 Turnover intention	2.49	0.91	−0.42*	1.00	
3 P-O fit	−0.35	0.78	0.24*	−0.19*	1.00

*Correlation is significant at the 0.01 level (2-tailed).

P-O fit, person-organisation fit.

### Hypothesis testing

#### Step 1

We tested the correlations between each of the three main indicators (table 3). All correlations were statistically significant (p<0.01). Both P-O fit (r=−0.19) and general job satisfaction (JS) (r=−0.42) were negatively correlated with turnover intention. P-O fit was positively correlated with general job satisfaction (r=0.244).

#### Step 2

We moved forward to use multiple regression model in which control variables were included. In model 1, P-O fit was used as an independent variable and JS as the dependent variable; in model 2, P-O fit was an independent variable while turnover intention was the dependent variable. Province, gender, age and professional title were treated as control variables in the two models and dummy variables were coded for provinces, age groups and professional titles.

In both models (table 4), gender and professional titles were not significantly correlated with the dependent variable (α=0.05), and thus were excluded. Model 1 revealed that P-O fit was positively associated with job satisfaction (standardised β=0.246, p<0.001). This supported Hypothesis 1. Model 2 showed that P-O fit was inversely correlated with turnover intention (standardised β=−0.186, p<0.001). Consequently, Hypothesis 2 was also supported.

**Table 4:** Two multiple regression models of JS on P-O fit and TI on P-O fit

	Standardised coefficients	p Value
Model 1: Regression of JS on P-O fit		
P-O fit	0.25	0.00
Province		
Shanghai	0.13	0.00
Shanxi	0.01	0.89
Shandong	0.06	0.22
Model 2: Regression of TI on P-O fit		
P-O fit	−0.19	0.00
Age group (years)		
>60	−0.06	0.10
50-	−0.10	0.02
40-	−0.12	0.01
30-	−0.07	0.10
Province		
Shanghai	−0.25	0.00
Shanxi	−0.12	0.01
Shandong	−0.16	0.00

JS, job satisfaction; P-O fit, person-organisation fit; TI, for turnover intention.

#### Step 3

**Figure 2:**
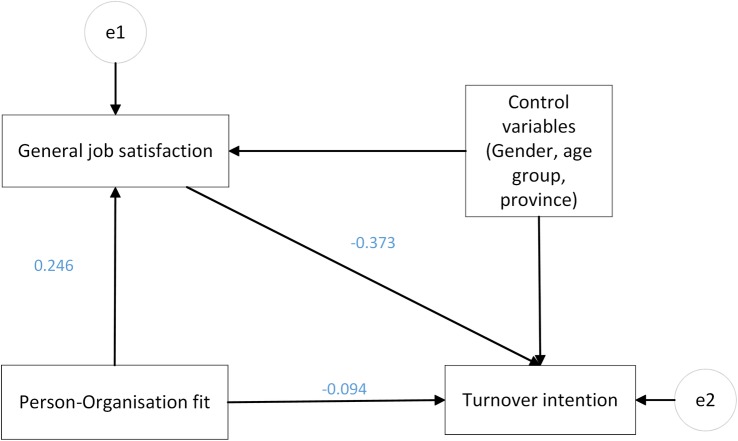
Mediation model with path analysis in AMOS. This figure shows that the effect of person-organisation fit on turnover intention is partially through job satisfaction. Control variables were included in the model. e1 and e2 are error terms, while rectangle icons are observed variables. A single-headed arrow shows the regression path from an independent variable to a dependent variable.

Path analysis was used to test mediation effect with control variables (province, gender, age, professional title) included in the model. As illustrated in figure 2, the Mediation Model accounted for the direct effect between P-O fit and turnover intention and also the indirect effect through job satisfaction. A good model fit was indicated by these fit indices: CMIN/DF=0.277, RMSEA=0.001, GFI=0.999, AGFI=0.997 and TLI=1.02. Since job satisfaction and turnover intention were both five-point Likert scale, bootstrap was performed to avoid the problem of multivariate non-normality. To calculate p value, we ran bias-corrected CIs.

It is notable that after the inclusion of job satisfaction, the effect of P-O fit on turnover intention decreased from −0.19 to −0.09 (table 5). The indirect effect on turnover intention was statistically significant (p<0.001). The results of Mediation Model suggest a partial mediation effect of job satisfaction on the relationship between P-O fit and turnover intention. However, the direct effect of P-O fit on turnover intention still existed. So Hypothesis 3 was not completely supported.

**Table 5:** Mediation effect test of job satisfaction on the relationship between P-O fit and turnover intention

	Total effect	Direct effect	Indirect effect
P-O fit → JS	–	0.25*	–
JS → TI	–	−0.37*	–
P-O fit → TI	−0.19*	−0.09†	−0.09*

These are all standardised effects.

*At the 0.001 level.

†Denotes statistical significance at the 0.01 level.

JS, job satisfaction; TI, turnover intention.

## Discussion

We conducted a mediation analysis to investigate the interrelationships between P-O fit, job satisfaction and turnover intention. Our results suggest that job satisfaction partially mediates the relationship between P-O fit and turnover intention. This study was among the first studies to explore relations among P-O fit, turnover intention and job satisfaction in human resources for health in the Chinese context.

Our study showed that the mean job satisfaction score of CHW was 3.55, which was close to the findings of Leiyu Shi in 2014 (3.35 to 3.57 for different specialties of CHWs).^25^ About 13.1% of CHWs had a clear turnover intention, lower than the finding reported by Sun *et al* in 2013 (38.7%).^26^ The similarity of job satisfaction findings with previous studies in China suggests that our findings could be reliable and generalisable in China. Furthermore, the sample size and the nation-level survey also support the generalisability of the findings in China.

Comparisons with international studies suggest both heterogeneity and similarity. Most P-O fit studies employed a subjective fit measurement to acquire the respondent's direct rating of their perception of congruence between the individual and the organisation.^27–29^ This study employed perceived indirect measurement and our findings were consistent with previous studies with indirect measurements. One study in the USA also found that a lack of P-O fit can lead to decreased job satisfaction and increased turnover intention.^12^ One study on nurses' P-O fit found that congruence on leadership practice was directly related to job satisfaction, and congruence on leadership support was inversely related to turnover intention.^10^ In Kristof's meta-analysis, results suggested that the effect of P-O fit from the needs-supplies dimension on job satisfaction was around 0.37,^4^ compared to which our result was lower (0.244). However, Verquer's meta-analysis found that P-O fit other than value congruence had an average effect of 0.24 on job satisfaction.^5^

Different from this study of mediation effect, one study in Turkey found that P-O fit was a moderator on the relationship of job satisfaction and intention.^30^ The different findings of two studies may rise from various causes. Instead of the needs-supplies congruence, the author used subjective direct measurement of P-O fit regarding personality, goal and value congruence. So the difference may attribute to the disparate measurements and/or dimensions of fit. On the other hand, the different findings of two studies could be compatible in a larger mechanism of the relationship among P-O fit, job satisfaction and turnover intention, which has not been fully explored yet.

Other than P-O fit and job satisfaction, age was also found to be significantly related to turnover intention. CHWs above 40 years' old were less possible to consider leaving their jobs, while in Leone's study in Portugal nurses from 35 to 39 had stronger intention to leave because they attempted to achieve career advancement in a better organistion or place.^31^ These findings were complementary, they could be explained by the fact that as health workers get older, they become more adapted to their work and less ambitious. Older as compared with younger workers exhibit more active problem-focused coping with strains in work, which reduce stress and intention to leave in older workers.^32^ Furthermore, older workers are more intrinsically than extrinsically motivated.^33^ Thus, they become less ambitious in pursuing wealth and status.

### Limitations

The study was limited in three aspects. First, as P-O fit research from needs-supplies dimension were few, we did not find any established questionnaire to make this study more comparable. Second, the number of attitudinal variables in this study were limited, which may explain why the total effect and direct effect were both small. Third, considering the vastness of China and its inner diversity in socioeconomic and cultural quality, we cannot claim the generalisability of our data, especially when taking the purposive sampling method into account. Nevertheless, multistage sampling and stratified sampling make our data representative of at least some of China's CHWs. As to the findings, confounders like provinces, age groups and professional titles were controlled in analysis, so that the influence of social factors and the population subgroup on the results was diminished. Therefore, as a piece of evidence of CHWs' P-O fit, the findings should have external validity for theory verification.

### Implications

The findings of this study indicate that P-O fit can help human resource managers identify the inconsistencies between the staff and the organisation to reduce dissatisfaction and turnover. For instance, indirect fit measurement, especially objective measurement of P-O fit, can be used in recruiting to find suitable workers. Specifically, an established organizstion profile is compared with the applicant's report of individual characteristics to assess P-O fit.^8^ ^34^ Therefore, more research and practice of P-O fit can help alleviate the persistent problem of workforce turnover in many developing countries.

Although the findings were consistent with previous job satisfaction research in China, we need to further understand the different or conflicted findings in existing studies. For this purpose, person-environment interaction variables (eg, work-life interaction) and other attitude variables (eg, organisation commitment) should be included to delineate the mechanism of person-organisation interaction and its attitude outcomes.

## Conclusions

We tested hypotheses regarding the relationships between P-O fit, general job satisfaction and turnover intention of CHWs in China. The results indicated that the effect of P-O fit on turnover intention was partially mediated by job satisfaction. We suggest the application of P-O fit scale in the area of human resources for health. Furthermore, there exist different, even conflicting; findings, the whole mechanism of the relationship among P-O fit, job satisfaction, turnover intention and other work attitude indicators should be explored.
