# Supplementary material for: Is the effect of person-organisation fit on turnover intention mediated by job satisfaction? A survey of community health workers in China
Source: BMJ Open. 2017 Feb 20;7(2):e013872. doi: 10.1136/bmjopen-2016-013872 (PMC5337699; doi:10.1136/bmjopen-2016-013872)
Supplement: supplementary file [file bmjopen-2016-013872supp.pdf]

variable definition is at the end of the file

ID	ORG	ORGID	DISTRICT	province	rank	quotastat	GENDER	MARRI	AGE	agegrp	WORKYEAR	post2	FULLTIME	mngment
1	安康	32	4	2	2	1	0	1	21	1	1	6	1	2
2	安康	32	4	2	2	2	0	1	21	1	2	6	1	2
3	安康	32	4	2	1	2	0	1	23	1	1	4	#NULL!	2
4	安康	32	4	2	1	2	0	1	24	1	3	6	1	2
5	安康	32	4	2	1	1	0	2	25	1	5	6	1	2
6	安康	32	4	2	1	2	0	1	26	1	1	1	1	2
7	安康	32	4	2	2	2	0	2	26	1	3	3	1	2
8	安康	32	4	2	3	1	0	1	26	1	3	3	1	2
9	安康	32	4	2	2	2	0	2	27	1	1	3	1	2
10	安康	32	4	2	2	2	0	2	27	1	7	5	1	2
11	安康	32	4	2	2	1	0	2	29	1	5	5	1	2
12	安康	32	4	2	3	1	0	2	34	2	1	5	1	2
13	安康	32	4	2	2	3	0	2	37	2	13	5	1	2
14	安康	32	4	2	2	3	0	2	38	2	15	6	1	#NULL!
15	安康	32	4	2	2	1	0	2	39	2	15	3	1	2
16	安康	32	4	2	3	3	0	2	51	4	32	2	#NULL!	2
17	安康	32	4	2	3	1	0	2	53	4	37	1	1	2
18	安康	32	4	2	1	1	1	1	24	1	1	4	1	2
19	安康	32	4	2	1	1	1	1	27	1	6	6	1	2
20	安康	32	4	2	2	1	1	2	32	2	6	2	1	2
21	柏景湾	3	1	1	2	2	0	1	22	1	1	5	2	2
22	柏景湾	3	1	1	2	1	0	1	22	1	2	5	1	2
23	柏景湾	3	1	1	2	2	0	2	56	4	30	2	2	2
24	柏景湾	3	1	1	2	2	1	2	36	2	10	2	2	2
25	宝铁	54	5	3	2	2	0	2	25	1	2	6	1	2
26	宝铁	54	5	3	2	1	0	2	32	2	10	4	2	2
27	宝铁	54	5	3	2	2	0	2	32	2	12	2	1	2
28	宝铁	54	5	3	2	1	0	2	37	2	16	2	2	2
29	宝铁	54	5	3	3	2	0	2	55	4	37	5	2	2
30	北站	60	7	4	2	2	0	1	23	1	3	5	1	2
31	北站	60	7	4	1	1	0	1	25	1	4	5	1	2
32	北站	60	7	4	2	2	0	1	26	1	4	4	1	2
33	北站	60	7	4	2	2	0	2	27	1	4	2	1	2
34	北站	60	7	4	2	1	0	1	27	1	5	5	1	2

ID	ORG	ORGID	DISTRICT	province	rank	quotastat	GENDER	MARRI	AGE	agegrp	WORKYEAR	post2	FULLTIME	mngment
35	北站	60	7	4	3	1	0	2	30	2	9	2	1	2
36	北站	60	7	4	2	1	0	2	33	2	14	5	1	2
37	北站	60	7	4	2	1	0	2	33	2	14	5	1	#NULL!
38	北站	60	7	4	3	1	0	3	35	2	17	5	1	1
39	北站	60	7	4	3	1	0	2	42	3	22	5	1	1
40	北站	60	7	4	3	1	0	2	45	3	23	2	1	1
41	北站	60	7	4	2	1	0	2	47	3	26	2	1	2
42	北站	60	7	4	3	1	1	2	35	2	13	2	1	1
43	北站	60	7	4	2	2	1	2	37	2	12	3	1	2
44	北站	60	7	4	3	1	1	2	37	2	13	2	1	2
45	北站	60	7	4	3	2	1	2	40	3	11	2	1	2
46	北站	60	7	4	2	1	1	1	40	3	18	2	1	2
47	亳州路	2	1	1	2	2	0	2	26	1	4	5	1	2
48	亳州路	2	1	1	2	2	0	2	27	1	6	5	1	2
49	亳州路	2	1	1	2	2	0	2	27	1	15	2	1	2
50	亳州路	2	1	1	2	2	0	1	29	1	5	5	1	2
51	亳州路	2	1	1	2	2	0	2	31	2	11	2	1	2
52	亳州路	2	1	1	2	1	0	2	32	2	11	5	1	2
53	亳州路	2	1	1	2	1	0	2	32	2	11	6	#NULL!	2
54	亳州路	2	1	1	2	2	0	2	34	2	13	5	1	2
55	亳州路	2	1	1	2	2	0	2	34	2	14	5	1	2
56	亳州路	2	1	1	2	1	0	2	34	2	16	5	1	1
57	亳州路	2	1	1	2	2	0	2	38	2	20	2	1	2
58	亳州路	2	1	1	2	1	0	2	43	3	18	5	1	2
59	亳州路	2	1	1	4	1	0	2	49	3	25	6	1	2
60	亳州路	2	1	1	3	1	0	2	53	4	33	6	1	2
61	亳州路	2	1	1	2	2	1	2	34	2	9	2	1	1
62	亳州路	2	1	1	3	1	1	2	35	2	14	2	1	2
63	亳州路	2	1	1	3	2	1	2	42	3	14	2	1	1
64	亳州路	2	1	1	4	1	1	2	46	3	21	3	1	2
65	亳州路	2	1	1	2	1	1	2	54	4	33	1	1	2
66	大湖	12	2	1	2	2	0	1	28	1	5	5	1	2
67	大湖	12	2	1	2	2	0	2	34	2	4	5	1	2
68	大湖	12	2	1	3	1	0	2	40	3	20	5	1	2

ID	ORG	ORGID	DISTRICT	province	rank	quotastat	GENDER	MARRI	AGE	agegrp	WORKYEAR	post2	FULLTIME	mngment
69	大庆	50	5	3	2	3	0	2	30	2	8	5	#NULL!	2
70	大庆	50	5	3	2	3	0	2	40	3	20	5	2	2
71	大庆	50	5	3	2	3	1	2	49	3	25	4	#NULL!	2
72	代王	56	6	3	2	1	0	1	25	1	2	6	1	2
73	代王	56	6	3	2	1	0	2	31	2	5	6	1	2
74	代王	56	6	3	2	1	0	2	34	2	13	5	1	2
75	代王	56	6	3	2	1	0	2	34	2	13	5	1	2
76	代王	56	6	3	3	1	0	2	36	2	17	5	1	2
77	代王	56	6	3	2	1	0	2	45	3	19	2	#NULL!	2
78	代王	56	6	3	3	1	0	2	45	3	25	5	1	1
79	代王	56	6	3	3	1	0	2	51	4	30	2	1	2
80	代王	56	6	3	2	1	1	1	30	2	5	4	1	2
81	代王	56	6	3	2	1	1	2	34	2	8	2	1	1
82	代王	56	6	3	3	1	1	2	45	3	22	6	1	2
83	德宽	15	2	1	2	2	0	1	20	1	1	5	1	2
84	德宽	15	2	1	2	2	0	1	22	1	1	5	2	2
85	德宽	15	2	1	2	1	0	1	23	1	1	5	1	2
86	德宽	15	2	1	2	2	0	1	24	1	2	6	1	2
87	德宽	15	2	1	2	2	0	2	34	2	12	2	1	2
88	德宽	15	2	1	2	2	0	2	35	2	6	2	1	2
89	德宽	15	2	1	2	2	0	2	42	3	20	5	1	2
90	德宽	15	2	1	3	2	0	2	43	3	20	4	#NULL!	2
91	德宽	15	2	1	2	2	1	2	31	2	5	3	1	1
92	德宽	15	2	1	3	2	1	2	47	3	30	2	1	1
93	甸柳	23	3	2	2	2	0	1	20	1	5	5	1	2
94	甸柳	23	3	2	2	2	0	1	23	1	2	5	1	2
95	甸柳	23	3	2	2	2	0	2	27	1	2	2	1	2
96	甸柳	23	3	2	2	2	0	1	28	1	3	4	1	2
97	甸柳	23	3	2	2	2	0	2	28	1	6	5	1	2
98	甸柳	23	3	2	2	2	0	1	28	1	6	5	1	2
99	甸柳	23	3	2	2	2	0	2	32	2	6	6	1	2
100	甸柳	23	3	2	3	2	0	2	33	2	4	2	1	2
101	甸柳	23	3	2	3	2	0	2	36	2	10	2	1	2
102	甸柳	23	3	2	4	1	0	2	40	3	16	1	1	2

ID	ORG	ORGID	DISTRICT	province	rank	quotastat	GENDER	MARRI	AGE	agegrp	WORKYEAR	post2	FULLTIME	mngment
103	甸柳	23	3	2	3	1	0	2	46	3	27	5	1	2
104	甸柳	23	3	2	3	1	0	2	52	4	32	5	1	2
105	甸柳	23	3	2	3	2	1	2	37	2	12	2	1	2
106	甸柳	23	3	2	3	1	1	2	38	2	17	2	1	1
107	东风路	45	5	3	1	2	0	1	20	1	2	6	1	2
108	东风路	45	5	3	2	2	0	1	23	1	4	5	1	2
109	东风路	45	5	3	2	2	0	1	24	1	1	6	2	2
110	东风路	45	5	3	2	1	0	1	25	1	1	6	1	2
111	东风路	45	5	3	2	2	0	1	25	1	4	6	1	1
112	东风路	45	5	3	2	2	0	2	26	1	5	6	2	2
113	东风路	45	5	3	2	2	0	2	26	1	7	5	1	2
114	东风路	45	5	3	2	2	0	2	29	1	5	6	1	2
115	东风路	45	5	3	2	2	0	2	29	1	8	5	1	2
116	东风路	45	5	3	2	2	0	2	29	1	9	5	2	2
117	东风路	45	5	3	2	1	0	2	29	1	10	6	#NULL!	2
118	东风路	45	5	3	2	2	0	2	30	2	7	5	1	2
119	东风路	45	5	3	2	2	0	2	30	2	9	5	2	2
120	东风路	45	5	3	3	2	0	2	30	2	9	5	1	2
121	东风路	45	5	3	2	2	0	2	31	2	12	5	2	2
122	东风路	45	5	3	2	2	0	2	33	2	10	5	1	2
123	东风路	45	5	3	2	2	0	2	33	2	12	5	1	2
124	东风路	45	5	3	2	2	0	2	37	2	15	2	1	2
125	东风路	45	5	3	3	2	0	2	37	2	17	4	1	1
126	东风路	45	5	3	3	2	0	2	37	2	19	6	2	2
127	东风路	45	5	3	2	1	0	2	40	3	16	6	1	2
128	东风路	45	5	3	3	2	0	2	57	4	38	5	1	2
129	东风路	45	5	3	2	2	1	1	30	2	8	6	1	2
130	东风路	45	5	3	2	1	1	2	40	3	19	6	#NULL!	1
131	东岭	51	5	3	2	1	1	2	38	2	13	3	1	2
132	斗鸡	47	5	3	2	1	0	2	36	2	19	6	#NULL!	2
133	斗鸡	47	5	3	2	1	0	2	39	2	19	6	#NULL!	2
134	斗鸡	47	5	3	2	1	0	2	42	3	23	2	1	2
135	斗鸡	47	5	3	4	1	0	2	47	3	2	3	1	#NULL!
136	敦化路	28	4	2	2	1	0	2	30	2	3	4	1	2

ID	ORG	ORGID	DISTRICT	province	rank	quotastat	GENDER	MARRI	AGE	agegrp	WORKYEAR	post2	FULLTIME	mngment
137	敦化路	28	4	2	2	1	0	2	32	2	11	6	1	2
138	敦化路	28	4	2	2	1	0	2	35	2	10	6	1	2
139	敦化路	28	4	2	3	1	0	2	40	3	20	5	1	1
140	敦化路	28	4	2	4	1	0	2	48	3	25	2	#NULL!	2
141	敦化路	28	4	2	3	1	0	2	55	4	36	2	#NULL!	2
142	敦化路	28	4	2	3	2	0	2	61	5	30	1	2	2
143	敦化路	28	4	2	1	1	1	2	26	1	2	4	1	2
144	佛山苑	17	3	2	1	2	0	1	24	1	2	5	1	2
145	佛山苑	17	3	2	2	2	0	2	24	1	3	5	1	2
146	佛山苑	17	3	2	2	2	0	2	27	1	6	5	1	2
147	佛山苑	17	3	2	2	2	0	2	33	2	13	5	1	2
148	佛山苑	17	3	2	3	2	0	2	62	5	40	2	2	2
149	佛山苑	17	3	2	3	2	1	2	58	4	37	6	1	2
150	福彩	34	4	2	1	2	0	1	23	1	1	5	1	2
151	福彩	34	4	2	2	2	0	1	25	1	4	5	2	2
152	福彩	34	4	2	2	2	0	1	27	1	4	6	1	2
153	福彩	34	4	2	2	2	0	2	31	2	10	6	1	2
154	福彩	34	4	2	2	2	0	2	35	2	11	5	1	2
155	福彩	34	4	2	3	3	0	2	64	5	45	1	#NULL!	2
156	钢厂	49	5	3	2	1	0	2	35	2	13	2	1	2
157	钢厂	49	5	3	3	1	0	2	43	3	23	5	1	2
158	钢厂	49	5	3	2	1	1	2	39	2	14	2	1	2
159	钢厂	49	5	3	4	1	1	2	52	4	29	1	1	1
160	高河梗	8	1	1	2	2	0	1	18	1	2	5	1	2
161	高河梗	8	1	1	2	2	0	2	48	3	20	1	1	2
162	高河梗	8	1	1	2	2	1	2	32	2	6	3	1	2
163	高河梗	8	1	1	2	2	1	2	35	2	12	2	1	1
164	高河梗	8	1	1	4	2	1	2	71	5	46	3	2	1
165	古城	9	1	1	2	2	0	1	22	1	2	5	1	2
166	古城	9	1	1	2	2	0	2	28	1	6	5	1	2
167	古城	9	1	1	2	2	1	2	30	2	5	3	1	2
168	古城	9	1	1	2	2	1	2	30	2	7	6	1	2
169	古城	9	1	1	2	2	1	2	38	2	11	2	1	1
170	海尔绿城	24	3	2	1	2	0	2	29	1	7	5	#NULL!	2

ID	ORG	ORGID	DISTRICT	province	rank	quotastat	GENDER	MARRI	AGE	agegrp	WORKYEAR	post2	FULLTIME	mngment
171	海尔绿城	24	3	2	2	2	1	2	35	2	11	2	1	2
172	海尔绿城	24	3	2	3	2	1	2	70	5	48	3	#NULL!	2
173	海棠街道	4	1	1	2	2	0	1	19	1	3	6	1	2
174	海棠街道	4	1	1	2	2	0	1	20	1	0	5	1	2
175	海棠街道	4	1	1	2	2	0	1	20	1	2	5	2	2
176	海棠街道	4	1	1	1	2	0	1	21	1	1	6	#NULL!	2
177	海棠街道	4	1	1	2	2	0	1	22	1	1	6	1	2
178	海棠街道	4	1	1	2	2	0	2	23	1	1	5	1	1
179	海棠街道	4	1	1	2	2	0	1	23	1	1	6	1	1
180	海棠街道	4	1	1	1	2	0	2	23	1	3	5	1	2
181	海棠街道	4	1	1	2	2	0	1	24	1	5	5	#NULL!	2
182	海棠街道	4	1	1	2	2	0	2	26	1	4	5	1	2
183	海棠街道	4	1	1	2	2	0	2	28	1	7	5	1	2
184	海棠街道	4	1	1	2	1	0	2	30	2	10	6	1	2
185	海棠街道	4	1	1	2	2	0	2	31	2	7	5	2	1
186	海棠街道	4	1	1	1	2	0	2	33	2	11	1	1	1
187	海棠街道	4	1	1	2	2	0	2	44	3	20	6	1	2
188	海棠街道	4	1	1	2	2	1	1	27	1	4	1	1	2
189	海棠街道	4	1	1	2	1	1	1	30	2	10	2	1	2
190	海棠街道	4	1	1	3	3	1	2	66	5	45	6	#NULL!	#NULL!
191	即墨路	29	4	2	2	2	0	1	25	1	7	6	1	2
192	即墨路	29	4	2	2	2	0	1	26	1	7	6	1	2
193	即墨路	29	4	2	2	2	0	1	27	1	7	5	1	2
194	即墨路	29	4	2	1	2	0	2	30	2	5	1	1	2
195	即墨路	29	4	2	2	1	0	2	31	2	2	3	1	2
196	即墨路	29	4	2	2	1	0	2	32	2	14	5	1	2
197	即墨路	29	4	2	3	1	0	2	33	2	13	6	1	2
198	即墨路	29	4	2	3	1	0	2	40	3	21	5	1	2
199	即墨路	29	4	2	1	1	0	2	49	3	26	6	1	2
200	即墨路	29	4	2	3	1	1	2	40	3	17	3	1	2
201	健民	30	4	2	1	2	0	1	22	1	1	1	1	2
202	健民	30	4	2	2	2	0	1	22	1	1	6	2	2
203	健民	30	4	2	2	2	0	1	22	1	2	6	2	2
204	健民	30	4	2	2	2	0	2	27	1	3	6	1	2

ID	ORG	ORGID	DISTRICT	province	rank	quotastat	GENDER	MARRI	AGE	agegrp	WORKYEAR	post2	FULLTIME	mngment
205	健民	30	4	2	2	1	0	2	27	1	4	5	1	1
206	健民	30	4	2	4	3	0	2	60	5	30	2	#NULL!	2
207	健民	30	4	2	1	2	1	1	26	1	2	1	1	2
208	健民	30	4	2	2	2	1	1	26	1	2	2	1	2
209	交口	57	6	3	1	2	0	1	23	1	1	5	2	2
210	交口	57	6	3	2	2	0	1	23	1	1	5	2	2
211	交口	57	6	3	1	1	0	1	23	1	1	6	1	2
212	交口	57	6	3	2	2	0	2	27	1	6	1	2	2
213	交口	57	6	3	3	2	0	2	32	2	7	1	1	2
214	交口	57	6	3	2	2	0	2	43	3	26	5	#NULL!	2
215	交口	57	6	3	1	1	1	1	25	1	2	6	1	2
216	交口	57	6	3	2	1	1	2	28	1	3	2	1	2
217	交口	57	6	3	3	1	1	2	54	4	33	6	1	2
218	骊山第一	58	6	3	2	1	0	2	31	2	10	5	1	2
219	骊山第一	58	6	3	1	1	0	2	33	2	10	5	1	2
220	骊山第一	58	6	3	1	1	0	2	35	2	13	6	#NULL!	#NULL!
221	骊山第一	58	6	3	2	1	0	2	36	2	15	5	1	2
222	骊山第一	58	6	3	3	1	0	2	38	2	15	2	1	1
223	骊山第一	58	6	3	2	1	0	2	41	3	13	2	1	2
224	骊山第一	58	6	3	2	1	0	2	41	3	23	2	1	2
225	骊山第一	58	6	3	2	1	1	2	34	2	10	2	1	1
226	骊山第一	58	6	3	2	1	1	2	46	3	23	2	1	2
227	辽宁路	37	4	2	2	2	0	1	24	1	2	5	1	2
228	辽宁路	37	4	2	2	1	0	2	26	1	3	5	1	2
229	辽宁路	37	4	2	2	2	0	1	26	1	3	5	1	2
230	辽宁路	37	4	2	2	1	0	1	26	1	3	6	1	2
231	辽宁路	37	4	2	2	1	0	2	29	1	3	2	1	2
232	辽宁路	37	4	2	2	2	0	2	31	2	10	6	1	2
233	辽宁路	37	4	2	3	1	0	2	42	3	20	5	1	1
234	辽宁路	37	4	2	3	1	0	2	42	3	21	5	1	1
235	菱湖	11	2	1	2	2	0	1	23	1	4	5	1	2
236	菱湖	11	2	1	1	2	0	1	25	1	1	2	1	2
237	菱湖	11	2	1	2	2	0	2	25	1	3	6	1	2
238	菱湖	11	2	1	1	2	0	1	26	1	1	1	1	2

ID	ORG	ORGID	DISTRICT	province	rank	quotastat	GENDER	MARRI	AGE	agegrp	WORKYEAR	post2	FULLTIME	mngment
239	菱湖	11	2	1	2	2	0	2	30	2	10	5	1	2
240	菱湖	11	2	1	2	2	0	2	31	2	6	2	1	2
241	菱湖	11	2	1	2	1	0	2	32	2	10	5	1	2
242	菱湖	11	2	1	2	1	0	2	33	2	14	2	1	2
243	菱湖	11	2	1	2	2	0	2	41	3	23	5	2	2
244	菱湖	11	2	1	3	1	0	2	44	3	21	6	1	1
245	菱湖	11	2	1	1	2	1	1	26	1	1	2	2	2
246	菱湖	11	2	1	2	1	1	2	28	1	6	2	1	1
247	菱湖	11	2	1	3	2	1	1	29	1	5	1	1	2
248	菱湖	11	2	1	1	2	1	2	37	2	7	1	2	2
249	和平	31	4	2	2	2	0	1	22	1	1	6	1	2
250	和平	31	4	2	1	2	0	1	23	1	1	1	1	2
251	和平	31	4	2	3	1	0	2	43	3	21	5	1	2
252	和平	31	4	2	3	2	0	2	46	3	21	5	1	2
253	和平	31	4	2	3	2	0	2	58	4	38	6	1	2
254	和平	31	4	2	3	2	0	2	64	5	38	1	2	2
255	和平	31	4	2	3	2	1	2	38	2	18	2	1	1
256	彭浦新村	61	7	4	2	2	0	1	20	1	1	5	1	2
257	彭浦新村	61	7	4	1	2	0	1	20	1	1	6	2	2
258	彭浦新村	61	7	4	2	2	0	2	24	1	3	5	1	2
259	彭浦新村	61	7	4	2	2	0	1	24	1	3	5	1	2
260	彭浦新村	61	7	4	1	1	0	2	26	1	1	4	2	2
261	彭浦新村	61	7	4	2	1	0	2	26	1	5	4	1	2
262	彭浦新村	61	7	4	2	1	0	1	30	2	10	5	1	2
263	彭浦新村	61	7	4	2	2	0	2	31	2	8	4	1	2
264	彭浦新村	61	7	4	2	1	0	2	32	2	11	5	1	2
265	彭浦新村	61	7	4	2	1	0	1	32	2	12	5	1	2
266	彭浦新村	61	7	4	2	1	0	2	33	2	12	5	2	2
267	彭浦新村	61	7	4	3	1	0	2	34	2	12	2	1	2
268	彭浦新村	61	7	4	3	1	0	2	34	2	15	5	1	2
269	彭浦新村	61	7	4	3	1	0	2	35	2	13	2	1	2
270	彭浦新村	61	7	4	3	1	0	2	35	2	13	2	1	2
271	彭浦新村	61	7	4	2	1	0	2	35	2	15	4	1	2
272	彭浦新村	61	7	4	3	1	0	1	36	2	16	6	1	2

ID	ORG	ORGID	DISTRICT	province	rank	quotastat	GENDER	MARRI	AGE	agegrp	WORKYEAR	post2	FULLTIME	mngment
273	彭浦新村	61	7	4	3	1	0	2	36	2	17	5	1	2
274	彭浦新村	61	7	4	3	1	0	2	37	2	10	2	1	2
275	彭浦新村	61	7	4	3	1	0	2	37	2	11	2	1	2
276	彭浦新村	61	7	4	3	1	0	2	37	2	13	2	1	2
277	彭浦新村	61	7	4	3	1	0	2	37	2	13	2	1	2
278	彭浦新村	61	7	4	2	1	0	2	37	2	18	2	1	2
279	彭浦新村	61	7	4	3	1	0	2	37	2	18	5	1	2
280	彭浦新村	61	7	4	2	1	0	2	38	2	19	5	1	2
281	彭浦新村	61	7	4	3	1	0	2	40	3	19	5	1	2
282	彭浦新村	61	7	4	3	1	0	2	40	3	21	5	1	2
283	彭浦新村	61	7	4	3	1	0	2	41	3	14	2	1	2
284	彭浦新村	61	7	4	3	1	0	2	41	3	22	5	1	2
285	彭浦新村	61	7	4	3	1	0	2	41	3	22	5	1	2
286	彭浦新村	61	7	4	2	1	0	2	41	3	23	5	1	2
287	彭浦新村	61	7	4	3	1	0	2	42	3	23	5	1	2
288	彭浦新村	61	7	4	1	1	0	2	42	3	23	5	1	2
289	彭浦新村	61	7	4	2	1	0	1	42	3	24	5	1	2
290	彭浦新村	61	7	4	3	1	0	2	43	3	23	2	1	2
291	彭浦新村	61	7	4	1	1	0	2	43	3	23	5	1	2
292	彭浦新村	61	7	4	3	1	0	2	43	3	23	5	1	2
293	彭浦新村	61	7	4	3	1	0	2	43	3	23	5	1	2
294	彭浦新村	61	7	4	3	1	0	2	43	3	24	2	1	2
295	彭浦新村	61	7	4	2	1	0	2	45	3	26	5	1	2
296	彭浦新村	61	7	4	3	1	0	2	46	3	27	5	1	2
297	彭浦新村	61	7	4	3	1	0	2	47	3	23	2	1	2
298	彭浦新村	61	7	4	3	1	0	2	47	3	28	5	1	2
299	彭浦新村	61	7	4	3	1	0	2	48	3	25	2	1	1
300	彭浦新村	61	7	4	3	1	0	2	49	3	23	2	1	2
301	彭浦新村	61	7	4	3	2	0	2	50	4	30	2	1	2
302	彭浦新村	61	7	4	2	2	1	2	33	2	7	2	1	2
303	彭浦新村	61	7	4	3	1	1	2	35	2	12	2	1	1
304	彭浦新村	61	7	4	3	1	1	2	36	2	13	2	1	2
305	彭浦新村	61	7	4	3	1	1	2	38	2	16	2	1	1
306	彭浦新村	61	7	4	3	1	1	2	41	3	12	2	1	2

ID	ORG	ORGID	DISTRICT	province	rank	quotastat	GENDER	MARRI	AGE	agegrp	WORKYEAR	post2	FULLTIME	mngment
307	彭浦新村	61	7	4	3	1	1	2	44	3	23	2	1	2
308	彭浦新村	61	7	4	3	1	1	2	56	4	37	2	1	2
309	彭浦镇	59	7	4	2	1	0	1	25	1	2	4	1	2
310	彭浦镇	59	7	4	2	2	0	2	26	1	2	5	1	2
311	彭浦镇	59	7	4	2	2	0	2	30	2	3	2	1	2
312	彭浦镇	59	7	4	2	1	0	2	30	2	7	5	1	2
313	彭浦镇	59	7	4	3	1	0	2	31	2	9	4	1	2
314	彭浦镇	59	7	4	3	1	0	2	33	2	9	2	1	2
315	彭浦镇	59	7	4	2	2	0	2	33	2	12	4	1	2
316	彭浦镇	59	7	4	3	1	0	2	35	2	14	4	1	2
317	彭浦镇	59	7	4	3	1	0	2	35	2	14	4	1	2
318	彭浦镇	59	7	4	2	1	0	2	35	2	14	5	1	2
319	彭浦镇	59	7	4	3	1	0	2	35	2	15	4	1	2
320	彭浦镇	59	7	4	3	1	0	2	35	2	16	5	1	2
321	彭浦镇	59	7	4	3	1	0	2	36	2	17	4	1	1
322	彭浦镇	59	7	4	3	1	0	2	40	3	18	5	1	2
323	彭浦镇	59	7	4	3	1	0	2	40	3	21	5	2	2
324	彭浦镇	59	7	4	3	1	0	2	42	3	11	2	1	2
325	彭浦镇	59	7	4	2	1	0	2	42	3	18	5	1	2
326	彭浦镇	59	7	4	1	2	0	2	43	3	7	4	1	1
327	彭浦镇	59	7	4	4	2	0	2	43	3	18	2	1	2
328	彭浦镇	59	7	4	3	1	0	2	43	3	22	2	1	2
329	彭浦镇	59	7	4	3	2	0	2	49	3	20	2	#NULL!	2
330	彭浦镇	59	7	4	3	1	1	2	28	1	5	2	1	2
331	彭浦镇	59	7	4	3	2	1	2	30	2	2	2	1	2
332	彭浦镇	59	7	4	2	1	1	2	31	2	9	4	1	2
333	彭浦镇	59	7	4	1	1	1	2	32	2	8	4	1	2
334	彭浦镇	59	7	4	3	1	1	2	37	2	17	2	1	#NULL!
335	棋盘	16	3	2	2	2	0	1	26	1	6	5	#NULL!	2
336	棋盘	16	3	2	2	2	0	2	34	2	12	4	1	2
337	棋盘	16	3	2	2	1	0	2	41	3	26	5	1	2
338	棋盘	16	3	2	3	1	0	2	45	3	20	5	1	2
339	棋盘	16	3	2	2	2	1	2	36	2	11	2	1	2
340	千佛山办	22	3	2	2	1	0	1	25	1	1	2	1	2

ID	ORG	ORGID	DISTRICT	province	rank	quotastat	GENDER	MARRI	AGE	agegrp	WORKYEAR	post2	FULLTIME	mngment
341	千佛山办	22	3	2	1	1	0	2	26	1	1	6	1	2
342	千佛山办	22	3	2	1	2	0	2	27	1	4	5	1	2
343	千佛山办	22	3	2	2	1	0	2	28	1	4	6	1	2
344	千佛山办	22	3	2	3	1	0	2	35	2	10	2	1	2
345	千佛山办	22	3	2	2	1	0	2	38	2	5	4	1	2
346	千佛山办	22	3	2	2	1	0	2	38	2	16	6	1	2
347	千佛山办	22	3	2	3	1	0	2	39	2	18	5	1	2
348	千佛山办	22	3	2	3	1	0	2	40	3	20	5	1	2
349	千佛山办	22	3	2	3	1	0	2	40	3	21	2	1	2
350	千佛山办	22	3	2	3	1	0	2	43	3	25	5	1	2
351	千佛山办	22	3	2	3	1	0	2	48	3	30	5	1	2
352	千佛山办	22	3	2	4	1	0	2	50	4	24	2	1	2
353	千佛山办	22	3	2	4	2	1	2	60	5	40	2	2	1
354	青后	19	3	2	2	2	0	1	25	1	1	4	1	2
355	青后	19	3	2	2	1	0	2	38	2	18	6	1	2
356	青后	19	3	2	3	1	0	2	48	3	29	5	1	2
357	青后	19	3	2	3	1	0	2	50	4	32	5	1	2
358	青后	19	3	2	2	1	1	2	50	4	30	6	#NULL!	2
359	青后	19	3	2	3	1	1	2	50	4	31	6	1	2
360	盛福中心	18	3	2	2	2	0	1	21	1	1	5	1	2
361	盛福中心	18	3	2	2	2	0	1	25	1	5	5	1	2
362	盛福中心	18	3	2	2	2	0	2	28	1	7	5	1	2
363	盛福中心	18	3	2	2	2	0	2	29	1	6	5	1	2
364	盛福中心	18	3	2	2	2	0	2	30	2	6	5	1	2
365	盛福中心	18	3	2	3	1	0	2	40	3	20	5	1	2
366	盛福中心	18	3	2	3	1	0	2	50	4	31	2	1	2
367	盛福中心	18	3	2	3	2	0	2	57	4	30	2	2	2
368	盛福中心	18	3	2	1	2	1	1	23	1	2	6	2	2
369	盛福中心	18	3	2	3	1	1	2	35	2	15	6	1	2
370	师东新村	25	3	2	2	2	0	1	24	1	1	5	1	2
371	师东新村	25	3	2	2	1	0	1	27	1	5	5	1	2
372	师东新村	25	3	2	1	2	0	1	29	1	4	5	1	2
373	师东新村	25	3	2	3	2	0	2	34	2	9	1	1	2
374	师东新村	25	3	2	1	1	0	2	48	3	3	6	1	1

ID	ORG	ORGID	DISTRICT	province	rank	quotastat	GENDER	MARRI	AGE	agegrp	WORKYEAR	post2	FULLTIME	mngment
375	师东新村	25	3	2	3	1	1	2	41	3	15	2	1	1
376	师东新村	25	3	2	1	1	1	2	44	3	3	6	1	1
377	师东新村	25	3	2	3	1	1	2	56	4	30	2	1	2
378	十亩园	20	3	2	2	2	0	1	26	1	1	3	1	2
379	十亩园	20	3	2	2	2	0	2	32	2	6	3	1	2
380	十亩园	20	3	2	1	1	0	2	35	2	11	6	#NULL!	1
381	十亩园	20	3	2	3	1	0	2	41	3	20	5	1	1
382	十亩园	20	3	2	2	1	0	2	52	4	36	6	1	2
383	十亩园	20	3	2	3	1	1	2	41	3	21	6	1	2
384	石油	46	5	3	2	2	0	1	18	1	1	5	2	2
385	石油	46	5	3	2	2	0	2	30	2	8	6	#NULL!	2
386	石油	46	5	3	2	2	0	2	32	2	10	6	2	2
387	石油	46	5	3	2	2	0	2	32	2	11	5	2	2
388	石油	46	5	3	2	2	0	2	34	2	12	6	1	2
389	石油	46	5	3	2	1	1	2	29	1	7	3	1	2
390	石油	46	5	3	1	2	1	2	41	3	21	2	2	1
391	石油	46	5	3	2	3	1	2	48	3	21	3	#NULL!	2
392	双岗	1	1	1	2	1	0	1	19	1	1	6	1	2
393	双岗	1	1	1	1	2	0	1	22	1	1	5	1	2
394	双岗	1	1	1	2	2	0	1	22	1	1	5	#NULL!	2
395	双岗	1	1	1	2	1	0	1	26	1	2	5	1	2
396	双岗	1	1	1	2	1	0	2	26	1	7	5	1	2
397	双岗	1	1	1	2	2	0	1	28	1	4	6	1	2
398	双岗	1	1	1	2	1	0	2	28	1	5	5	1	2
399	双岗	1	1	1	2	1	0	2	28	1	7	5	1	2
400	双岗	1	1	1	2	2	0	2	29	1	7	6	1	2
401	双岗	1	1	1	2	2	0	2	30	2	7	5	1	2
402	双岗	1	1	1	3	2	0	2	33	2	8	2	1	2
403	双岗	1	1	1	3	1	0	2	33	2	11	5	1	1
404	双岗	1	1	1	2	1	0	2	35	2	14	5	1	2
405	双岗	1	1	1	2	2	0	2	36	2	12	2	1	2
406	双岗	1	1	1	2	1	0	2	36	2	15	5	1	2
407	双岗	1	1	1	2	1	0	2	41	3	21	6	1	2
408	双岗	1	1	1	3	1	0	2	41	3	22	6	1	2

ID	ORG	ORGID	DISTRICT	province	rank	quotastat	GENDER	MARRI	AGE	agegrp	WORKYEAR	post2	FULLTIME	mngment
409	双岗	1	1	1	3	1	0	2	43	3	21	2	1	1
410	双岗	1	1	1	2	2	0	2	47	3	18	5	2	2
411	双岗	1	1	1	3	2	1	2	33	2	8	2	1	1
412	双岗	1	1	1	2	2	1	2	34	2	13	3	1	2
413	双岗	1	1	1	3	2	1	2	39	2	14	2	1	1
414	双岗	1	1	1	3	2	1	2	41	3	18	2	1	1
415	双岗	1	1	1	2	1	1	2	42	3	10	2	1	5
416	双桃站	39	4	2	2	2	0	1	23	1	1	5	1	2
417	双桃站	39	4	2	2	2	0	2	27	1	4	5	1	2
418	双桃站	39	4	2	2	1	0	2	29	1	8	6	1	2
419	双桃站	39	4	2	2	2	0	2	30	2	6	5	1	2
420	双桃站	39	4	2	2	3	0	2	30	2	8	5	1	2
421	双桃站	39	4	2	2	2	0	2	38	2	9	3	1	2
422	双桃站	39	4	2	2	1	0	2	40	3	20	5	2	2
423	双桃站	39	4	2	1	2	0	2	42	3	19	5	1	2
424	双桃站	39	4	2	2	1	0	2	43	3	17	6	1	2
425	双桃站	39	4	2	3	1	0	2	45	3	20	1	1	#NULL!
426	双桃站	39	4	2	3	3	0	2	60	5	36	1	#NULL!	2
427	双桃中心	40	4	2	1	2	0	1	23	1	1	6	1	2
428	双桃中心	40	4	2	3	1	0	2	35	2	10	2	1	1
429	双桃中心	40	4	2	2	1	0	2	37	2	17	5	#NULL!	2
430	双桃中心	40	4	2	3	3	0	2	54	4	36	6	1	2
431	双桃中心	40	4	2	2	1	1	2	55	4	25	2	1	2
432	四总队	48	5	3	2	1	0	2	42	3	17	6	1	2
433	四总队	48	5	3	3	1	0	2	42	3	20	5	1	2
434	四总队	48	5	3	4	1	0	2	54	4	30	2	1	1
435	登州路	36	4	2	2	2	0	1	21	1	2	5	1	2
436	登州路	36	4	2	1	2	0	2	27	1	2	6	1	2
437	登州路	36	4	2	2	2	0	2	27	1	7	6	1	2
438	登州路	36	4	2	2	2	0	2	29	1	5	6	1	2
439	登州路	36	4	2	2	2	0	1	29	1	6	5	1	2
440	登州路	36	4	2	2	2	0	1	32	2	10	5	1	2
441	登州路	36	4	2	2	2	0	1	33	2	10	5	1	2
442	登州路	36	4	2	3	2	0	2	39	2	18	2	1	2

ID	ORG	ORGID	DISTRICT	province	rank	quotastat	GENDER	MARRI	AGE	agegrp	WORKYEAR	post2	FULLTIME	mngment
443	登州路	36	4	2	2	2	1	2	31	2	9	2	1	2
444	台东八路	27	4	2	2	1	0	1	25	1	2	5	1	2
445	台东八路	27	4	2	2	1	0	1	25	1	3	6	1	2
446	台东八路	27	4	2	2	1	0	1	25	1	5	3	1	2
447	台东八路	27	4	2	2	1	0	2	30	2	5	4	1	2
448	台东八路	27	4	2	2	2	0	2	30	2	9	6	1	2
449	台东八路	27	4	2	2	1	0	2	31	2	4	4	1	2
450	台东八路	27	4	2	3	1	0	2	47	3	23	2	1	2
451	台东八路	27	4	2	2	3	0	2	52	4	35	5	#NULL!	2
452	台东八路	27	4	2	2	3	0	2	53	4	33	6	1	2
453	台东八路	27	4	2	3	2	0	2	59	4	35	6	2	#NULL!
454	台东八路	27	4	2	2	2	1	1	27	1	1	2	1	2
455	亭南	14	2	1	1	2	0	1	22	1	3	6	2	2
456	亭南	14	2	1	2	2	0	2	26	1	5	5	1	2
457	亭南	14	2	1	2	2	0	2	34	2	10	5	1	2
458	亭南	14	2	1	2	2	0	2	41	3	12	2	1	1
459	亭南	14	2	1	1	1	1	2	41	3	6	4	1	1
460	亭南	14	2	1	3	2	1	2	47	3	24	2	#NULL!	2
461	王家碾	53	5	3	1	2	0	1	22	1	1	5	2	2
462	王家碾	53	5	3	2	2	0	2	25	1	5	6	2	2
463	王家碾	53	5	3	3	2	0	1	32	2	1	5	#NULL!	2
464	王家碾	53	5	3	2	2	0	2	42	3	21	5	2	2
465	王家碾	53	5	3	3	3	0	2	57	4	36	3	#NULL!	2
466	阜新	38	4	2	1	2	0	1	21	1	0	4	1	2
467	阜新	38	4	2	1	2	0	1	25	1	4	5	1	2
468	阜新	38	4	2	2	2	0	1	27	1	4	6	1	2
469	阜新	38	4	2	3	1	0	2	37	2	19	3	1	1
470	阜新	38	4	2	3	3	0	2	60	5	42	5	#NULL!	2
471	阜新	38	4	2	4	2	1	2	60	5	25	1	1	2
472	卧龙寺站	42	5	3	3	1	0	2	33	2	13	5	1	1
473	卧龙寺站	42	5	3	2	1	0	2	37	2	12	3	1	2
474	卧龙寺站	42	5	3	3	1	0	2	37	2	17	1	1	2
475	卧龙寺站	42	5	3	1	1	0	2	41	3	20	2	1	2
476	卧龙寺中	43	5	3	2	1	0	1	23	1	2	5	2	2

ID	ORG	ORGID	DISTRICT	province	rank	quotastat	GENDER	MARRI	AGE	agegrp	WORKYEAR	post2	FULLTIME	mngment
477	卧龙寺中	43	5	3	3	2	0	1	23	1	2	6	1	2
478	卧龙寺中	43	5	3	2	2	0	1	25	1	1	5	1	2
479	卧龙寺中	43	5	3	2	2	0	2	25	1	2	5	2	2
480	卧龙寺中	43	5	3	2	2	0	1	26	1	2	6	#NULL!	2
481	卧龙寺中	43	5	3	2	2	0	2	30	2	6	6	1	2
482	卧龙寺中	43	5	3	2	1	0	2	30	2	9	6	1	2
483	卧龙寺中	43	5	3	2	2	0	2	32	2	10	5	1	2
484	卧龙寺中	43	5	3	1	2	0	2	34	2	11	5	2	2
485	卧龙寺中	43	5	3	2	2	0	2	36	2	14	5	1	2
486	卧龙寺中	43	5	3	2	2	0	2	38	2	18	5	#NULL!	2
487	卧龙寺中	43	5	3	2	2	0	2	38	2	18	5	2	2
488	卧龙寺中	43	5	3	2	2	0	2	42	3	20	4	1	2
489	卧龙寺中	43	5	3	3	1	0	2	45	3	22	2	#NULL!	2
490	卧龙寺中	43	5	3	2	2	0	4	46	3	28	6	1	2
491	卧龙寺中	43	5	3	2	1	0	2	49	3	26	2	1	2
492	卧龙寺中	43	5	3	2	1	0	2	49	3	30	5	1	2
493	卧龙寺中	43	5	3	3	3	0	2	55	4	34	2	1	2
494	卧龙寺中	43	5	3	3	1	1	2	28	1	6	3	2	2
495	卧龙寺中	43	5	3	2	2	1	2	30	2	4	2	1	2
496	卧龙寺中	43	5	3	3	3	1	2	53	4	32	2	#NULL!	2
497	无棣路	35	4	2	1	2	0	1	25	1	3	4	1	2
498	无棣路	35	4	2	2	1	0	1	27	1	3	2	1	2
499	无棣路	35	4	2	2	1	0	2	31	2	12	5	1	2
500	无棣路	35	4	2	3	1	0	2	38	2	19	5	1	1
501	无棣路	35	4	2	4	1	0	2	49	3	32	5	1	2
502	无棣路	35	4	2	2	1	1	2	31	2	11	6	1	1
503	无棣路	35	4	2	3	1	1	2	47	3	21	2	1	1
504	西关	41	5	3	2	2	0	1	22	1	2	5	#NULL!	#NULL!
505	西关	41	5	3	1	2	0	1	23	1	1	4	1	2
506	西关	41	5	3	2	1	0	1	23	1	1	5	1	2
507	西关	41	5	3	1	2	0	1	23	1	1	6	#NULL!	2
508	西关	41	5	3	1	1	0	2	28	1	3	5	2	2
509	西关	41	5	3	2	2	0	2	30	2	10	5	1	2
510	西关	41	5	3	2	2	0	2	31	2	11	4	1	2

ID	ORG	ORGID	DISTRICT	province	rank	quotastat	GENDER	MARRI	AGE	agegrp	WORKYEAR	post2	FULLTIME	mngment
511	西关	41	5	3	2	1	0	2	33	2	11	6	1	2
512	西关	41	5	3	2	1	0	2	37	2	17	5	1	1
513	西关	41	5	3	2	1	0	2	38	2	13	2	1	2
514	西关	41	5	3	2	1	0	2	40	3	22	5	1	1
515	西关	41	5	3	2	1	0	2	41	3	22	5	1	2
516	西关	41	5	3	1	1	0	2	43	3	22	6	1	1
517	西关	41	5	3	3	1	0	2	50	4	27	2	1	2
518	西关	41	5	3	2	2	0	2	56	4	36	6	#NULL!	2
519	西关	41	5	3	2	1	1	2	29	1	7	6	1	2
520	西关	41	5	3	2	2	1	2	35	2	12	1	2	2
521	西关	41	5	3	3	1	1	2	42	3	20	1	1	2
522	西关	41	5	3	2	1	1	2	42	3	27	6	1	1
523	西关	41	5	3	3	1	1	2	50	4	27	6	1	2
524	逍遥津	6	1	1	2	2	0	1	23	1	3	5	1	#NULL!
525	逍遥津	6	1	1	2	2	0	1	24	1	3	5	1	2
526	逍遥津	6	1	1	2	2	0	1	24	1	4	5	1	2
527	逍遥津	6	1	1	2	2	0	1	25	1	2	6	1	2
528	逍遥津	6	1	1	1	2	0	2	27	1	4	5	1	2
529	逍遥津	6	1	1	2	2	0	2	28	1	4	2	1	2
530	逍遥津	6	1	1	2	2	0	2	28	1	4	5	1	2
531	逍遥津	6	1	1	2	2	0	2	28	1	13	6	1	2
532	逍遥津	6	1	1	1	2	0	2	31	2	5	6	1	2
533	逍遥津	6	1	1	2	2	0	2	32	2	10	5	1	2
534	逍遥津	6	1	1	2	2	0	2	33	2	13	5	1	2
535	逍遥津	6	1	1	2	1	0	2	34	2	14	2	1	2
536	逍遥津	6	1	1	2	2	0	2	34	2	14	4	1	2
537	逍遥津	6	1	1	2	1	0	2	36	2	6	2	1	2
538	逍遥津	6	1	1	2	2	0	2	36	2	13	5	1	2
539	逍遥津	6	1	1	2	1	0	2	39	2	10	1	1	1
540	逍遥津	6	1	1	2	2	0	2	43	3	19	6	#NULL!	2
541	逍遥津	6	1	1	3	1	0	2	43	3	25	3	1	2
542	逍遥津	6	1	1	2	2	1	1	26	1	3	3	1	2
543	逍遥津	6	1	1	2	1	1	2	30	2	3	5	1	2
544	逍遥津	6	1	1	2	1	1	2	30	2	10	6	1	2

ID	ORG	ORGID	DISTRICT	province	rank	quotastat	GENDER	MARRI	AGE	agegrp	WORKYEAR	post2	FULLTIME	mngment
545	逍遥津	6	1	1	1	1	1	2	34	2	7	4	1	1
546	逍遥津	6	1	1	2	1	1	2	35	2	11	2	1	2
547	逍遥津	6	1	1	1	2	1	2	37	2	15	6	1	2
548	逍遥津	6	1	1	2	2	1	2	38	2	18	3	1	2
549	逍遥津	6	1	1	3	2	1	2	40	3	16	6	1	2
550	逍遥津	6	1	1	3	1	1	2	43	3	18	6	#NULL!	1
551	逍遥津	6	1	1	3	1	1	2	45	3	22	3	1	2
552	逍遥津	6	1	1	2	1	1	2	48	3	26	2	#NULL!	2
553	逍遥津	6	1	1	2	1	1	2	59	4	37	6	#NULL!	2
554	小港	33	4	2	1	2	0	1	26	1	2	1	2	2
555	小港	33	4	2	2	2	0	2	26	1	4	5	1	2
556	小港	33	4	2	2	2	0	2	30	2	8	5	1	2
557	小港	33	4	2	2	2	0	2	37	2	13	5	2	2
558	小港	33	4	2	2	2	1	1	26	1	3	3	1	2
559	新福路	55	5	3	2	2	0	1	22	1	1	5	1	2
560	新福路	55	5	3	3	2	0	2	47	3	23	2	2	2
561	新福路	55	5	3	1	2	1	2	25	1	2	4	1	1
562	杏花街道	5	1	1	1	2	0	1	23	1	1	4	1	1
563	杏花街道	5	1	1	2	2	0	1	23	1	2	5	1	2
564	杏花街道	5	1	1	2	2	0	1	23	1	2	5	1	2
565	杏花街道	5	1	1	1	1	0	2	26	1	4	5	2	2
566	杏花街道	5	1	1	2	1	0	2	26	1	5	5	1	2
567	杏花街道	5	1	1	2	2	0	2	27	1	5	1	1	2
568	杏花街道	5	1	1	2	1	0	2	29	1	8	5	1	1
569	杏花街道	5	1	1	2	2	0	2	30	2	7	2	1	2
570	杏花街道	5	1	1	2	2	0	2	32	2	12	5	1	1
571	杏花街道	5	1	1	2	1	0	2	34	2	10	6	1	2
572	杏花街道	5	1	1	2	2	0	2	35	2	7	5	#NULL!	2
573	杏花街道	5	1	1	1	1	0	2	47	3	27	5	1	2
574	杏花街道	5	1	1	1	2	1	1	26	1	1	1	1	2
575	杏花街道	5	1	1	2	2	1	2	28	1	7	3	1	2
576	杏花街道	5	1	1	2	1	1	2	31	2	6	1	1	1
577	杏花街道	5	1	1	1	1	1	2	38	2	10	4	1	1
578	杏花街道	5	1	1	2	1	1	2	41	3	20	4	1	2

ID	ORG	ORGID	DISTRICT	province	rank	quotastat	GENDER	MARRI	AGE	agegrp	WORKYEAR	post2	FULLTIME	mngment
579	杏花街道	5	1	1	3	1	1	2	52	4	30	6	1	1
580	延安路	26	4	2	2	2	0	1	25	1	3	6	1	2
581	延安路	26	4	2	2	1	0	2	25	1	5	6	1	2
582	延安路	26	4	2	1	1	0	1	26	1	2	1	#NULL!	2
583	延安路	26	4	2	2	1	0	1	27	1	2	1	1	2
584	延安路	26	4	2	2	1	0	1	27	1	2	4	1	2
585	延安路	26	4	2	2	1	0	2	27	1	3	6	1	2
586	延安路	26	4	2	2	2	0	2	27	1	7	5	1	2
587	延安路	26	4	2	2	1	0	1	28	1	8	5	1	2
588	延安路	26	4	2	2	1	0	2	35	2	12	5	1	2
589	延安路	26	4	2	2	1	0	2	37	2	15	5	1	2
590	延安路	26	4	2	2	2	0	2	37	2	16	6	1	2
591	延安路	26	4	2	3	1	0	1	39	2	15	4	1	2
592	延安路	26	4	2	1	2	0	2	43	3	20	6	1	2
593	延安路	26	4	2	2	1	0	2	47	3	29	5	#NULL!	2
594	延安路	26	4	2	2	3	0	2	54	4	30	4	#NULL!	2
595	延安路	26	4	2	3	2	0	2	56	4	37	5	2	2
596	延安路	26	4	2	3	1	1	2	33	2	6	2	1	2
597	延安路	26	4	2	2	1	1	2	53	4	35	6	1	#NULL!
598	姚家	21	3	2	2	2	0	1	22	1	1	5	1	2
599	姚家	21	3	2	2	2	0	1	22	1	1	5	1	2
600	姚家	21	3	2	2	2	0	1	24	1	2	4	1	2
601	姚家	21	3	2	2	2	0	1	25	1	1	5	1	2
602	姚家	21	3	2	1	2	0	2	25	1	2	1	1	2
603	姚家	21	3	2	2	1	0	2	25	1	6	4	2	2
604	姚家	21	3	2	1	2	0	2	26	1	2	1	1	2
605	姚家	21	3	2	2	2	0	1	26	1	6	5	1	2
606	姚家	21	3	2	2	1	0	1	27	1	1	1	1	2
607	姚家	21	3	2	2	2	0	2	28	1	7	5	1	2
608	姚家	21	3	2	2	1	0	2	29	1	6	5	1	2
609	姚家	21	3	2	2	2	0	2	30	2	5	5	1	2
610	姚家	21	3	2	2	2	0	2	31	2	5	5	1	2
611	姚家	21	3	2	3	1	0	2	32	2	11	2	1	2
612	姚家	21	3	2	3	1	0	2	33	2	7	2	1	2

ID	ORG	ORGID	DISTRICT	province	rank	quotastat	GENDER	MARRI	AGE	agegrp	WORKYEAR	post2	FULLTIME	mngment
613	姚家	21	3	2	3	1	0	2	35	2	11	4	1	2
614	姚家	21	3	2	2	2	0	2	36	2	13	5	1	2
615	姚家	21	3	2	2	2	0	2	38	2	3	2	1	2
616	姚家	21	3	2	3	1	0	2	44	3	21	5	1	1
617	姚家	21	3	2	3	1	0	2	44	3	24	1	1	2
618	姚家	21	3	2	3	1	0	2	48	3	28	5	1	1
619	姚家	21	3	2	1	2	1	1	26	1	1	1	2	2
620	引渭路	52	5	3	2	1	0	2	24	1	1	5	1	2
621	引渭路	52	5	3	1	1	0	2	28	1	1	6	1	1
622	引渭路	52	5	3	2	1	0	2	34	2	11	1	2	2
623	引渭路	52	5	3	3	1	1	2	33	2	8	2	1	2
624	玉琳路	10	2	1	2	2	0	1	21	1	1	5	2	2
625	玉琳路	10	2	1	2	2	0	1	25	1	6	5	1	2
626	玉琳路	10	2	1	3	1	0	2	38	2	16	5	1	1
627	玉琳路	10	2	1	3	1	0	2	39	2	20	5	1	1
628	玉琳路	10	2	1	3	1	0	2	43	3	21	4	1	1
629	玉琳路	10	2	1	3	1	0	2	45	3	20	1	1	2
630	玉琳路	10	2	1	3	1	0	2	45	3	24	5	1	1
631	玉琳路	10	2	1	3	1	0	3	48	3	30	5	1	2
632	玉琳路	10	2	1	3	1	0	2	49	3	29	5	1	2
633	玉琳路	10	2	1	2	1	1	2	37	2	13	1	1	2
634	玉琳路	10	2	1	2	1	1	2	42	3	18	1	#NULL!	#NULL!
635	玉琳路	10	2	1	3	1	1	2	44	3	20	1	1	1
636	玉琳路	10	2	1	3	1	1	2	46	3	27	1	1	2
637	玉琳路	10	2	1	3	1	1	2	50	4	33	1	#NULL!	2
638	玉琳路	10	2	1	4	1	1	2	51	4	28	1	1	1
639	玉琳路	10	2	1	3	2	1	2	52	4	32	2	1	1
640	玉琳路	10	2	1	3	1	1	2	54	4	34	3	1	1
641	中山路	44	5	3	2	1	0	2	33	2	10	4	1	2
642	中山路	44	5	3	2	1	0	2	33	2	12	6	1	2
643	中山路	44	5	3	2	1	0	2	36	2	13	4	1	2
644	中山路	44	5	3	2	1	0	2	38	2	15	1	1	2
645	中山路	44	5	3	3	1	0	2	38	2	17	1	#NULL!	2
646	中山路	44	5	3	2	1	0	2	39	2	12	6	1	2

ID	ORG	ORGID	DISTRICT	province	rank	quotastat	GENDER	MARRI	AGE	agegrp	WORKYEAR	post2	FULLTIME	mngment
647	中山路	44	5	3	3	1	0	2	40	3	19	4	1	2
648	中山路	44	5	3	3	1	0	2	41	3	22	5	1	2
649	中山路	44	5	3	2	2	0	2	42	3	20	5	2	2
650	中山路	44	5	3	3	1	0	2	42	3	22	5	1	2
651	中山路	44	5	3	3	1	0	2	44	3	25	5	1	2
652	中山路	44	5	3	3	1	0	2	44	3	26	5	1	1
653	中山路	44	5	3	3	1	0	2	49	3	31	5	1	2
654	中山路	44	5	3	3	1	1	2	36	2	13	2	1	2
655	中山路	44	5	3	3	1	1	2	41	3	17	3	1	2
656	中山路	44	5	3	4	1	1	2	48	3	24	2	1	2

ID	MEDEDUCA	WESTMED	CTM	WCM	PREVENT	NURSING	MEDLAB	ORAL	PHARM	GPAE	REHAB	HEALTHMA	OTHERAE
1	2	N	N	N	N	N	N	N	N	N	N	Y	
2	2	N	N	N	N	N	N	N	Y	N	N	N	
3	2	N	N	N	N	N	N	N	N	N	N	Y	
4	2	N	N	N	N	N	N	N	Y	N	N	N	
5	2	N	N	N	N	N	Y	N	N	N	N	N	
6	3	Y	N	N	N	N	N	N	N	N	N	N	
7	2	N	Y	N	N	N	N	N	N	N	N	N	
8	2	N	Y	N	N	N	N	N	N	N	N	N	
9	4	N	Y	N	N	N	N	N	N	N	N	N	
10	2	Y	N	N	Y	Y	N	N	Y	Y	N	Y	
11	2	N	N	N	N	Y	N	N	N	N	N	N	
12	3	N	N	N	N	Y	N	N	N	N	N	N	
13	1	Y	N	N	Y	Y	N	N	Y	N	N	N	
14	1	N	N	N	N	N	N	N	Y	N	N	N	
15	3	N	Y	N	N	N	N	N	Y	N	N	N	
16	1	Y	N	N	N	N	N	N	N	N	N	N	
17	2	Y	N	N	N	N	N	N	N	N	N	N	
18	2												
19	2	N	Y	N	N	N	N	N	N	N	N	N	
20	2	Y	N	N	N	N	N	N	N	N	N	N	
21	1	N	N	N	N	Y	N	N	N	N	N	N	
22	1	N	N	N	N	Y	N	N	N	N	N	N	
23	2	Y	N	N	N	N	N	N	N	N	N	N	
24	2	N	N	Y	N	N	N	N	N	N	N	N	
25	2	N	N	N	N	N	Y	N	N	N	N	N	
26	2	Y	N	N	N	N	N	N	N	N	N	N	
27	2	Y	N	N	N	N	N	N	N	N	N	N	
28	2	Y	N	N	N	N	N	N	N	N	N	N	
29	1	N	N	N	N	Y	N	N	N	N	N	N	
30	1	N	N	N	N	Y	N	N	N	N	N	N	
31	2	N	N	N	N	Y	N	N	N	N	N	N	
32	3	N	N	N	Y	N	N	N	N	N	N	N	
33	3	Y	N	N	N	N	N	N	N	N	N	N	
34	3	N	N	N	N	Y	N	N	N	N	N	N	

ID	MEDEDUCA	WESTMED	CTM	WCM	PREVENT	NURSING	MEDLAB	ORAL	PHARM	GPAE	REHAB	HEALTHMA	OTHERAE
35	3	Y	N	N	N	N	N	N	N	N	N	N	
36	2	N	N	N	N	Y	N	N	N	N	N	N	
37	2	N	N	N	N	Y	N	N	N	N	N	N	
38	3	N	N	N	N	Y	N	N	N	N	N	N	
39	3	N	N	N	N	Y	N	N	N	N	N	N	
40	2	Y	N	N	N	N	N	N	N	N	N	N	
41	2	Y	N	N	N	N	N	N	N	Y	N	N	
42	3	Y	N	N	N	N	N	N	N	Y	N	N	
43	2	N	Y	N	N	N	N	N	N	N	N	N	
44	3	Y	N	N	N	N	N	N	N	N	N	N	
45	3	Y	N	N	N	N	N	N	N	N	N	N	
46	3	Y	N	N	N	N	N	N	N	N	N	N	
47	2	N	N	N	N	Y	N	N	N	Y	N	N	
48	1	N	N	N	N	Y	N	N	N	N	N	N	
49	1	Y	N	N	N	N	N	N	N	N	N	N	
50	2	N	N	N	N	Y	N	N	N	N	N	N	
51	3	Y	N	N	N	N	N	N	N	N	N	N	
52	1	N	N	N	N	Y	N	N	N	N	N	N	
53	2	N	N	N	N	N	N	N	Y	N	N	N	
54	2	N	N	N	N	Y	N	N	N	N	N	N	
55	2	N	N	N	N	Y	N	N	N	N	N	N	
56	1	N	N	N	N	Y	N	N	N	N	N	N	
57	2	Y	N	N	N	N	N	N	N	Y	N	N	
58	1	N	N	N	N	Y	N	N	N	N	N	N	
59	3	N	N	N	N	N	N	N	Y	N	N	N	
60	2	N	N	N	N	N	Y	N	N	N	N	N	
61	2	Y	N	N	N	N	N	N	N	N	N	N	
62	3	Y	N	N	N	N	N	N	N	N	N	N	
63	3	Y	N	N	N	N	N	N	N	Y	N	N	
64	3	N	Y	N	N	N	N	N	N	N	N	N	
65	1	Y	N	N	N	N	N	N	N	N	N	N	
66	3	N	N	N	N	Y	N	N	N	N	N	N	
67	1	N	N	N	N	Y	N	N	N	N	N	N	
68	1	N	N	N	N	Y	N	N	N	N	N	N	

ID	MEDEDUCA	WESTMED	CTM	WCM	PREVENT	NURSING	MEDLAB	ORAL	PHARM	GPAE	REHAB	HEALTHMA	OTHERAE
69	1	N	N	N	N	Y	N	N	N	N	N	N	
70	1	Y	N	N	N	N	N	N	N	N	N	N	
71	2												
72	2	N	N	N	N	N	N	N	Y	N	N	N	
73	3	Y	N	N	N	N	N	N	N	N	N	N	
74	1	N	N	N	N	Y	N	N	N	N	N	N	
75	2	N	N	N	N	Y	N	N	N	N	N	N	
76	2	N	Y	N	N	N	N	N	N	N	N	N	
77	2	Y	N	N	N	N	N	N	N	N	N	N	
78	1	N	N	N	N	Y	N	N	N	N	N	N	
79	1	Y	N	N	N	N	N	N	N	N	N	N	
80	2	Y	N	N	N	N	N	N	N	N	N	N	
81	2	Y	N	N	N	N	N	N	N	N	N	N	
82	2	N	N	N	N	N	Y	N	N	N	N	N	
83	1	N	N	N	N	Y	N	N	N	N	N	N	
84	2	N	N	N	N	Y	N	N	N	N	N	N	
85	3	N	N	N	N	Y	N	N	N	N	N	N	
86	2	N	N	N	N	N	N	N	Y	N	N	N	
87	3	N	Y	N	N	N	N	N	N	N	N	N	
88	2	N	N	Y	N	N	N	N	N	N	N	N	
89	2	N	N	N	N	Y	N	N	N	N	N	N	
90	1	N	N	N	N	Y	N	N	N	N	N	N	
91	3	N	N	Y	N	N	N	N	N	N	N	N	
92	2	N	Y	N	N	N	N	N	N	Y	Y	N	
93	1					Y							
94	2					Y							
95	3	Y											
96	2				Y								
97	2					Y							
98	2					Y							
99	2	Y											
100	4		Y										
101	4	Y								Y			
102	4	Y											

ID	MEDEDUCA	WESTMED	CTM	WCM	PREVENT	NURSING	MEDLAB	ORAL	PHARM	GPAE	REHAB	HEALTHMA	OTHERAE
103	2				Y	Y							
104	1					Y							
105	3			Y									
106	3	Y								Y			
107	1	N	N	N	N	N	N	N	N	N	Y	N	
108	2	N	N	N	N	Y	N	N	N	N	N	N	
109	3	N	N	N	N	N	Y	N	N	N	N	N	
110	5												
111	2	N	N	N	N	N	N	N	Y	N	N	N	
112	2	N	N	N	N	N	Y	N	N	N	N	N	
113	1	N	N	N	N	N	N	N	N	N	N	N	Y
114	2	N	N	N	N	N	N	N	Y	N	N	N	
115	2	N	N	N	N	Y	N	N	N	N	N	N	
116	2	N	N	N	N	Y	N	N	N	N	N	N	
117	1	N	N	N	N	N	N	N	Y	N	N	N	
118	1	N	N	Y	N	Y	N	N	N	N	N	N	
119	2	N	N	N	N	Y	N	N	N	N	N	N	
120	5	N	N	N	N	Y	N	N	N	N	N	N	
121	2	N	N	N	N	Y	N	N	N	N	N	N	
122	2	N	N	N	N	Y	N	N	N	N	N	N	
123	2	N	N	N	N	Y	N	N	N	N	N	N	
124	2	Y	N	N	N	N	N	N	N	N	N	N	
125	3	N	N	N	Y	N	N	N	N	N	N	N	
126	1	N	N	N	N	N	N	N	Y	N	N	N	
127	2	N	N	N	N	N	N	N	Y	N	N	N	
128	1	N	N	N	N	Y	N	N	N	N	N	N	
129	1	Y	N	N	N	N	N	N	Y	N	N	N	
130	1	N	N	N	N	N	N	N	Y	N	N	N	
131	2	N	Y	N	N	N	N	N	N	N	N	N	
132	2	N	N	N	N	Y	N	N	N	N	N	N	
133	2	N	N	N	N	Y	N	N	N	N	N	N	
134	2	Y	N	N	N	N	N	N	N	N	N	N	
135	3	N	Y	N	N	N	N	N	N	N	N	N	
136	4	N	N	N	Y	N	N	N	N	N	N	N	Y

ID	MEDEDUCA	WESTMED	CTM	WCM	PREVENT	NURSING	MEDLAB	ORAL	PHARM	GPAE	REHAB	HEALTHMA	OTHERAE
137	2	N	N	N	N	N	N	N	Y	N	N	N	N
138	2	N	N	N	N	N	N	N	N	N	N	N	Y
139	3	N	N	N	Y	N	N	N	N	N	N	N	N
140	3	N	Y	Y	N	N	N	N	N	Y	N	N	N
141	2	Y	N	N	N	N	N	N	N	N	N	N	N
142	2	Y	N	N	N	N	N	N	N	N	N	N	N
143	3	N	N	N	Y	N	N	N	N	N	N	N	N
144	2					Y							
145	2					Y							
146	1					Y							
147	2					Y							
148	2	Y								Y			
149	1								Y				
150	2	N	N	N	N	Y	N	N	N	N	N	N	N
151	1	N	N	N	N	Y	N	N	N	N	N	N	N
152	2	N	N	N	N	N	N	N	Y	N	N	N	N
153	1	N	N	N	N	N	Y	N	N	N	N	N	N
154	2	N	N	N	N	Y	N	N	N	N	N	N	N
155	1	Y	N	N	N	N	N	N	N	N	N	N	N
156	1	Y	N	N	N	N	N	N	N	N	N	N	
157	2												
158	2	N	Y	N	N	N	N	N	N	N	N	N	
159	2	Y	Y	Y	Y	N	N	N	N	Y	Y	N	
160	1	N	N	N	N	Y	N	N	N	N	N	N	
161	2												
162	3	N	Y	N	N	N	N	N	N	N	N	N	
163	2	Y	N	N	N	N	N	N	N	N	N	N	
164	3	N	Y	N	N	N	N	N	N	N	N	N	
165	1	N	N	N	N	Y	N	N	N	N	N	N	
166	1	Y	N	N	N	Y	N	N	N	N	N	N	
167	2	N	Y	N	N	N	N	N	N	N	N	N	
168	2	N	N	N	N	N	Y	N	N	N	N	N	
169	2	Y	N	Y	N	N	N	N	N	N	N	N	
170	2	N	N	N	N	Y	N	N	N	N	N	N	N

ID	MEDEDUCA	WESTMED	CTM	WCM	PREVENT	NURSING	MEDLAB	ORAL	PHARM	GPAE	REHAB	HEALTHMA	OTHERAE
171	2	N	Y	N	N	N	N	N	N	N	N	N	N
172	2	N	Y	Y	N	N	N	N	N	N	N	N	N
173	1	N	N	N	N	Y	N	N	N	N	N	N	
174	2	N	N	N	N	N	N	N	N	Y	N	N	
175	1	N	N	N	N	Y	N	N	N	N	N	N	
176	1	N	N	N	N	Y	N	N	N	N	N	N	
177	2	N	N	N	N	N	N	N	Y	N	N	N	
178	2	N	N	N	N	Y	N	N	N	N	N	N	
179	2	N	N	N	N	N	N	N	Y	N	N	N	
180	2	N	N	N	N	Y	N	N	N	N	N	N	
181	5	N	N	N	N	Y	N	N	N	N	N	N	
182	3	N	N	N	N	Y	N	N	N	N	N	N	
183	2	N	N	N	N	Y	N	N	N	N	N	N	
184	1	N	N	N	N	Y	N	N	N	N	N	N	
185	3	N	N	N	N	Y	N	N	N	N	N	N	
186	2	Y	N	N	N	N	N	N	N	N	N	N	
187	1	Y	N	N	N	N	Y	N	N	N	N	N	
188	3	N	N	Y	N	N	N	N	N	N	N	N	
189	3	Y	N	N	N	N	N	N	N	N	N	N	
190	5	N	N	N	N	N	N	N	N	N	N	N	Y
191	2	N	N	N	N	N	N	N	Y	N	N	N	N
192	2	N	N	N	N	N	N	N	N	N	Y	N	N
193	2	N	N	N	N	Y	N	N	N	N	N	N	N
194	3	Y	N	N	N	N	N	N	N	N	N	N	N
195	4	N	Y	N	N	N	N	N	N	N	N	N	N
196	2	N	N	N	N	Y	N	N	N	N	N	N	N
197	3	N	N	N	N	N	N	N	Y	N	N	N	N
198	2	N	N	N	N	Y	N	N	N	N	N	N	N
199	5												
200	2	N	Y	N	N	N	N	N	N	N	N	N	N
201	2	Y											
202	2					Y							
203	2					Y							
204	2					Y							

ID	MEDEDUCA	WESTMED	CTM	WCM	PREVENT	NURSING	MEDLAB	ORAL	PHARM	GPAE	REHAB	HEALTHMA	OTHERAE
205	3					Y							
206	2	Y											
207	2	Y											
208	2	Y											
209	2	N	N	N	N	Y	N	N	N	N	N	N	
210	2	Y	N	N	N	Y	N	N	Y	N	N	N	
211	2	N	N	N	N	N	Y	N	N	N	N	N	
212	3	Y	N	N	N	N	N	N	N	N	N	N	
213	3	N	N	Y	N	N	N	N	N	N	N	N	
214	2	N	N	N	N	Y	N	N	N	N	N	N	
215	3	N	N	N	N	N	Y	N	N	N	N	N	
216	3	N	N	N	N	N	N	N	N	Y	N	N	
217	5	Y	N	N	N	N	N	N	N	N	N	N	
218	3	N	N	N	N	Y	N	N	N	N	N	N	
219	3	N	N	N	N	Y	N	N	N	N	N	N	
220	5												
221	2	N	N	N	N	Y	N	N	N	N	N	N	
222	3	Y	N	N	N	N	N	N	N	N	N	N	
223	2	Y	N	N	N	N	N	N	N	N	N	N	
224	2	Y	N	N	N	N	N	N	N	N	N	N	
225	3	Y	N	N	N	N	N	N	N	N	N	N	
226	1	Y	N	N	N	N	N	N	N	N	N	N	
227	1	N	N	N	N	Y	N	N	N	N	N	N	N
228	1	N	N	N	N	Y	N	N	N	N	N	N	N
229	1	N	N	N	N	Y	N	N	N	N	N	N	N
230	5												
231	3	Y	N	N	N	N	N	N	N	N	N	N	N
232	2	N	N	N	N	N	Y	N	N	N	N	N	N
233	2	N	N	N	N	Y	N	N	N	N	N	N	N
234	1	N	N	N	N	Y	N	N	N	N	N	N	N
235	1												
236	3	N	N	Y	N	N	N	N	N	N	N	N	
237	2	N	N	N	N	N	N	N	Y	N	N	N	
238	3	N	N	Y	N	N	N	N	N	N	N	N	

ID	MEDEDUCA	WESTMED	CTM	WCM	PREVENT	NURSING	MEDLAB	ORAL	PHARM	GPAE	REHAB	HEALTHMA	OTHERAE
239	2	N	N	N	N	Y	N	N	N	N	N	N	
240	3	Y	N	N	N	N	N	N	N	N	N	N	
241	2	N	N	N	N	Y	N	N	N	N	N	N	
242	3	Y	N	N	N	N	N	N	N	N	N	N	
243	1	N	N	N	N	Y	N	N	N	N	N	N	
244	2	N	N	N	N	Y	N	N	N	N	N	N	
245	3	N	N	Y	N	N	N	N	N	N	N	N	
246	3	Y	N	N	N	N	N	N	N	Y	N	N	
247	1	N	N	N	N	N	N	N	N	N	N		
248	2	Y	N	N	N	N	N	N	N	N	N	N	
249	3								Y				
250	2	Y											
251	2	Y											
252	1	Y											
253	2								Y				
254	2	Y											
255	3	Y								Y			
256	2	N	N	N	N	Y	N	N	N	N	N	N	
257	1	N	N	N	N	N	N	N	Y	N	N	N	
258	2	N	N	N	N	Y	N	N	N	N	N	N	
259	2	N	N	N	N	Y	N	N	N	N	N	N	
260	3	N	Y	N	Y	N	N	N	N	N	N	N	
261	3	N	N	N	Y	N	N	N	N	N	N	N	
262	3	N	N	N	N	Y	N	N	N	N	N	N	
263	3	N	N	N	Y	N	N	N	N	N	N	N	
264	3	N	N	N	N	Y	N	N	N	N	N	N	
265	2	N	N	N	N	Y	N	N	N	N	N	N	
266	2	N	N	N	N	Y	N	N	N	N	N	N	
267	3	Y	N	N	N	N	N	N	N	N	N	N	
268	2	N	N	N	N	Y	N	N	N	N	N	N	
269	3	Y	N	N	N	N	N	N	N	N	N	N	
270	3	Y	N	N	N	N	N	N	N	N	N	N	
271	2	N	N	N	Y	N	N	N	N	N	N	N	
272	3	N	N	N	N	Y	N	N	N	N	N	N	

ID	MEDEDUCA	WESTMED	CTM	WCM	PREVENT	NURSING	MEDLAB	ORAL	PHARM	GPAE	REHAB	HEALTHMA	OTHERAE
273	2	N	N	N	N	Y	N	N	N	N	N	N	
274	3	Y	N	N	N	N	N	N	N	N	N	N	
275	3	Y	N	N	N	N	N	N	N	N	N	N	
276	3	Y	N	N	N	N	N	N	N	Y	N	N	
277	3	Y	N	N	N	N	N	N	N	N	N	N	
278	3	Y	N	N	N	N	N	N	N	N	N	N	
279	3	N	N	N	N	Y	N	N	N	N	N	N	
280	2	N	N	N	N	Y	N	N	N	N	N	N	
281	2	N	N	N	N	Y	N	N	N	N	N	N	
282	2	N	N	N	N	Y	N	N	N	N	N	N	
283	3	Y	N	N	N	N	N	N	N	N	N	N	
284	2	N	N	N	N	Y	N	N	N	N	N	N	
285	3	N	N	N	N	Y	N	N	N	N	N	N	
286	2	Y	N	N	N	N	N	N	N	N	N	N	
287	3	N	N	N	N	Y	N	N	N	N	N	N	
288	1	Y	N	N	N	N	N	N	N	N	N	N	
289	2	N	N	N	N	Y	N	N	N	N	N	N	
290	3	Y	N	N	N	N	N	N	N	Y	N	N	
291	2	N	N	N	N	Y	N	N	N	N	N	N	
292	2	N	N	N	N	Y	N	N	N	N	N	N	
293	3	N	N	N	N	Y	N	N	N	N	N	N	
294	3	Y	N	N	N	N	N	N	N	Y	N	N	
295	2	N	N	N	N	Y	N	N	N	N	N	N	
296	2	N	N	N	N	Y	N	N	N	N	N	N	
297	3	Y	N	N	N	N	N	N	N	N	N	N	
298	2	N	N	N	N	Y	N	N	N	N	N	N	
299	3	Y	N	N	N	N	N	N	N	Y	N	N	
300	3	Y	N	N	N	N	N	N	N	N	N	N	
301	2	Y	N	N	N	N	N	N	N	N	N	N	
302	3	N	N	N	N	N	N	N	N	Y	N	N	
303	3	Y	N	N	N	N	N	N	N	Y	N	N	
304	3	Y	N	N	N	N	N	N	N	N	N	N	
305	3	Y	N	N	N	N	N	N	N	Y	N	N	
306	3	Y	N	N	N	N	N	N	N	N	N	N	

ID	MEDEDUCA	WESTMED	CTM	WCM	PREVENT	NURSING	MEDLAB	ORAL	PHARM	GPAE	REHAB	HEALTHMA	OTHERAE
307	3	Y	N	N	N	N	N	N	N	Y	N	N	
308	2	Y	N	N	N	N	N	N	N	N	N	N	
309	2	N	N	N	Y	N	N	N	N	N	N	N	
310	2	N	N	N	N	Y	N	N	N	N	N	N	
311	3	Y	N	N	N	N	N	N	N	N	N	N	
312	2	N	N	N	N	Y	N	N	N	N	N	N	
313	2	N	N	N	Y	N	N	N	N	N	N	N	
314	3	Y	N	N	N	N	N	N	N	Y	N	N	
315	2	N	N	N	N	Y	N	N	N	N	N	N	
316	3	N	N	N	Y	N	N	N	N	N	N	N	
317	3	Y	N	N	N	N	N	N	N	N	N	N	
318	2	N	N	N	N	Y	N	N	N	N	N	N	
319	3	N	N	N	Y	N	N	N	N	N	N	N	
320	2	N	N	N	N	Y	N	N	N	N	N	N	
321	3	N	N	N	Y	N	N	N	N	N	N	N	
322	3	N	N	N	N	Y	N	N	N	N	N	N	
323	3	N	N	N	N	Y	N	N	N	N	N	N	
324	3	Y	N	N	N	N	N	N	N	N	N	N	
325	2	N	N	N	N	Y	N	N	N	N	N	N	
326	1	N	N	N	Y	N	N	N	N	N	N	N	
327	3	Y	N	N	N	N	N	N	N	N	N	N	
328	2	Y	N	N	N	N	N	N	N	N	N	N	
329	3	Y	N	N	N	N	N	N	N	Y	N	N	
330	3	Y	N	N	N	N	N	N	N	N	N	N	
331	3	N	N	N	N	N	N	N	N	Y	N	N	
332	3	N	N	N	Y	N	N	N	N	N	N	N	
333	3	N	N	N	Y	N	N	N	N	N	N	N	
334	3	Y	N	N	N	N	N	N	N	N	N	N	
335	2	N	N	N	N	Y	N	N	N	N	N	N	N
336	2	Y	N	Y	N	N	N	N	N	N	N	N	N
337	2	N	N	N	N	Y	N	N	N	N	N	N	N
338	2	N	N	N	N	N	N	Y	N	N	N	N	N
339	2	N	N	Y	N	N	N	N	N	N	N	N	N
340	3	Y											

ID	MEDEDUCA	WESTMED	CTM	WCM	PREVENT	NURSING	MEDLAB	ORAL	PHARM	GPAE	REHAB	HEALTHMA	OTHERAE
341	5												
342	3					Y							
343	3								Y				
344	3	Y											
345	3				Y								
346	3						Y						
347	2					Y							
348	3					Y							
349	3	Y											
350	2					Y							
351	1					Y							
352	3	Y											
353	2									Y			
354	2	N	N	N	N	Y	N	N	N	N	N	N	N
355	5	N	N	N	N	N	N	N	Y	N	N	N	N
356	3	N	N	N	N	Y	N	N	N	N	N	N	N
357	1	N	N	N	N	Y	N	N	N	N	N	N	N
358	1	N	N	N	N	N	N	N	Y	N	N	N	N
359	2	N	N	N	N	N	N	N	Y	N	N	N	N
360	2	N	N	N	N	Y	N	N	N	N	N	N	
361	2	N	N	N	N	Y	N	N	N	N	N	N	
362	2	N	N	N	N	Y	N	N	N	N	N	N	
363	2	N	N	N	N	Y	N	N	N	N	N	N	
364	2	N	N	N	N	Y	N	N	N	N	N	N	
365	3	Y	N	N	N	N	N	N	N	N	N	N	
366	2	N	Y	N	N	N	N	N	N	N	N	N	
367	2	Y	N	N	N	N	N	N	N	N	N	N	
368	2	N	N	N	N	N	N	Y	N	N	N	N	
369	2	N	N	N	N	N	N	Y	N	N	N	N	
370	2	N	N	N	N	Y	N	N	N	N	N	N	N
371	2	N	N	N	N	Y	N	N	N	N	N	N	N
372	1	N	N	N	N	Y	N	N	N	N	N	N	N
373	2	Y	N	N	N	N	N	N	N	N	N	N	N
374	5												

ID	MEDEDUCA	WESTMED	CTM	WCM	PREVENT	NURSING	MEDLAB	ORAL	PHARM	GPAE	REHAB	HEALTHMA	OTHERAE
375	3	N	Y	Y	N	N	N	N	N	N	N	N	N
376	5												
377	2	Y	N	Y	N	N	N	N	N	N	N	N	N
378	4	N	Y	N	N	N	N	N	N	N	N	N	Y
379	3	N	Y	N	N	N	N	N	N	N	N	N	N
380	2	N	N	N	N	Y	N	N	N	N	N	N	N
381	3	N	N	N	N	Y	N	N	N	N	N	N	N
382	1	N	N	N	N	N	Y	N	N	N	N	N	N
383	3	N	N	N	N	N	N	N	Y	N	N	N	N
384	2	N	N	N	N	Y	N	N	N	N	N	N	
385	1	N	N	N	N	Y	N	N	N	N	N	N	
386	2	N	N	N	N	Y	N	N	N	N	N	N	
387	1	N	N	N	N	Y	N	N	N	N	N	N	
388	2	N	N	N	N	N	N	N	N	Y	N	N	
389	2	N	Y	N	N	N	N	N	N	N	N	N	
390	3	Y	N	N	N	N	N	N	N	N	N	N	
391	3	Y	Y	N	N	N	N	N	N	N	N	N	
392	2		N	N	N	N	Y	N	N	N	N	N	
393	1	N	N	N	N	Y	N	N	N	N	N	N	
394	2	N	N	N	N	Y	N	N	N	N	N	N	
395	2	N	N	N	N	Y	N	N	N	N	N	N	
396	2	N	N	N	N	Y	N	N	N	N	N	N	
397	3	N	N	N	N	N	N	N	Y	N	N	N	
398	3	N	N	N	N	Y	N	N	N	N	N	N	
399	2	N	N	N	N	Y	N	N	N	N	N	N	
400	2	N	N	N	N	N	N	N	Y	N	N	N	
401	2	N	N	N	N	Y	N	N	N	N	N	N	
402	3	N	N	Y	N	N	N	N	N	N	N	N	
403	2	N	N	N	N	Y	N	N	N	N	N	N	
404	2	N	N	N	N	Y	N	N	N	Y	N	N	
405	2	Y	N	N	N	N	N	N	N	Y	N	N	
406	2	N	N	N	N	Y	N	N	N	N	N	N	
407	2	N	N	N	N	N	Y	N	N	N	N	N	
408	2	N	N	N	N	Y	N	N	N	N	N	N	

ID	MEDEDUCA	WESTMED	CTM	WCM	PREVENT	NURSING	MEDLAB	ORAL	PHARM	GPAE	REHAB	HEALTHMA	OTHERAE
409	2	Y	N	N	N	N	N	N	N	N	N	N	
410	1	N	N	N	N	Y	N	N	N	N	N	N	
411	3	N	N	Y	N	N	N	N	N	N	N	N	
412	3	N	N	Y	N	N	N	N	N	N	N	N	
413	3	Y	N	Y	N	N	N	N	N	N	N	N	
414	2	Y	N	N	N	N	N	N	N	N	N	N	
415	3	Y	N	N	N	N	N	N	N	Y	N	N	
416	2					Y							
417	1					Y							
418	2												Y
419	1					Y							
420	2					Y							
421	1		Y							Y			
422	1					Y							
423	1					Y							
424	2					Y							
425	3	Y											
426	2	Y											
427	2								Y				
428	3		Y										
429	1					Y							
430	2							Y					
431	2	Y											
432	2	N	N	N	N	Y	N	N	N	N	N	N	
433	2	N	N	N	N	Y	N	N	N	N	N	N	
434	3	Y	Y	N	N	N	N	N	N	Y	N	N	
435	2					Y							
436	5												
437	2						Y						
438	3								Y				
439	3					Y							
440	2					Y							
441	2					Y							
442	3	Y											

ID	MEDEDUCA	WESTMED	CTM	WCM	PREVENT	NURSING	MEDLAB	ORAL	PHARM	GPAE	REHAB	HEALTHMA	OTHERAE
443	2	Y											
444	3	N	N	N	N	Y	N	N	N	N	N	N	N
445	3	N	N	N	N	N	N	N	Y	N	N	N	N
446	2	N	Y	N	N	N	N	N	N	N	N	N	N
447	3	N	N	N	N	Y	N	N	N	N	N	N	N
448	3	N	N	N	N	N	N	N	Y	N	N	N	N
449	4	N	N	N	Y	N	N	N	N	N	N	N	N
450	3	N	Y	Y	N	N	N	N	N	N	N	N	N
451	2	N	N	N	N	Y	N	N	N	N	N	N	N
452	5	N	N	N	N	N	N	N	Y	N	N	N	N
453	2	N	N	N	N	N	Y	N	N	N	N	N	N
454	4	Y	N	N	N	N	N	N	N	N	N	N	N
455	1	N	N	N	N	Y	N	N	N	N	N	N	
456	2	N	N	N	N	Y	N	N	N	N	N	N	
457	2	N	N	N	N	Y	N	N	N	N	N	N	
458	2	N	N	N	N	N	N	N	N	Y	N	N	
459	5	N	N	N	N	N	N	N	N	N	N	Y	
460	2	Y	Y	N	N	N	N	N	N	Y	N	N	
461	1	N	N	N	N	Y	N	N	N	N	N	N	
462	1	N	N	N	N	N	N	N	Y	N	N	N	
463	5	N	N	N	N	Y	N	N	N	N	N	N	
464	1	N	N	N	N	Y	N	N	N	N	N	N	
465	1	N	Y	N	N	N	N	N	N	N	N	N	
466	2	N	Y	N	N	N	N	N	N	N	N	N	N
467	2	N	N	N	N	Y	N	N	N	N	N	N	N
468	2	N	N	N	N	N	N	N	Y	N	N	N	N
469	3	Y	N	N	N	Y	N	N	N	N	N	N	N
470	1	N	N	N	N	Y	N	N	N	N	N	N	N
471	3	Y	N	Y	N	N	N	N	N	N	N	N	N
472	3	N	N	N	N	Y	N	N	N	N	N	N	
473	2	N	N	N	N	N	N	N	N	N	Y	N	
474	2	Y	N	N	N	N	N	N	N	N	N	N	
475	2	N	Y	Y	N	N	N	N	N	N	N	N	
476	2	Y	N	N	N	Y	N	N	N	N	N	N	

ID	MEDEDUCA	WESTMED	CTM	WCM	PREVENT	NURSING	MEDLAB	ORAL	PHARM	GPAE	REHAB	HEALTHMA	OTHERAE
477	3	N	N	N	N	N	N	N	N	N	Y	N	
478	2	N	N	N	N	Y	N	N	N	N	N	N	
479	2	N	N	N	N	Y	N	N	N	N	N	N	
480	2	N	Y	N	N	N	N	N	N	N	N	N	
481	2	N	N	N	N	N	Y	N	N	N	N	N	
482	3	N	Y	N	N	N	N	N	N	N	N	N	
483	2	N	N	N	N	Y	N	N	N	N	N	N	
484	2	Y	N	N	N	N	N	N	N	N	N	N	
485	2	N	N	N	N	Y	N	N	N	N	N	N	
486	1	N	N	N	N	Y	N	N	N	N	N	N	
487	2	N	N	N	N	Y	N	N	N	N	N	N	
488	2	Y	Y	N	N	N	N	N	N	N	N	N	
489	2	Y	N	N	N	N	N	N	N	N	N	N	
490	1	N	N	N	N	N	Y	N	N	N	N	N	
491	1	Y	N	N	N	N	N	N	N	N	N	N	
492	1	N	N	N	N	Y	N	N	N	N	N	N	
493	1	Y	N	N	N	N	N	N	N	N	N	N	
494	1	N	Y	N	N	N	N	N	N	N	N	N	
495	3	N	Y	N	N	N	N	N	N	N	N	N	
496	3	N	N	Y	N	N	N	N	N	N	N	N	
497	3	N	N	N	N	N	N	N	Y	N	N	N	N
498	2	Y	N	N	N	N	N	N	N	N	N	N	N
499	2	N	N	N	N	Y	N	N	N	N	N	N	N
500	3	N	N	N	N	Y	N	N	N	N	N	N	N
501	3	N	N	N	N	Y	N	N	N	N	N	N	N
502	2	N	N	N	N	N	Y	N	N	N	N	N	N
503	3	Y	N	N	N	N	N	N	N	N	N	N	N
504	1	N	N	N	N	Y	N	N	N	N	N	N	
505	2	N	N	N	N	N	N	N	N	N	Y	N	
506	2	N	N	N	N	Y	N	N	N	N	N	N	
507	2	N	Y	N	N	N	N	N	N	N	N	N	
508	2												
509	2	N	N	N	N	Y	N	N	N	N	N	N	
510	2	Y	N	N	N	N	N	N	N	Y	N	N	

ID	MEDEDUCA	WESTMED	CTM	WCM	PREVENT	NURSING	MEDLAB	ORAL	PHARM	GPAE	REHAB	HEALTHMA	OTHERAE
511	2	N	N	N	N	N	Y	N	N	N	N	N	
512	2	N	N	N	N	Y	N	N	N	Y	N	N	
513	3	N	Y	N	N	N	N	N	N	N	N	N	
514	1	N	N	N	N	Y	N	N	N	N	N	N	
515	2	N	N	N	N	Y	N	N	N	N	N	N	
516	5	N	N	N	N	N	N	N	N	N	N	N	Y
517	1	Y	N	N	N	N	N	N	N	N	N	N	
518	5												
519	2	N	N	Y	N	N	N	N	N	N	Y	N	
520	1	N	N	Y	N	N	N	N	N	N	N	N	
521	2	N	N	N	N	N	Y	N	N	N	N	N	
522	5												
523	1	N	N	N	N	N	N	N	Y	N	N	N	
524	2	N	N	N	N	Y	N	N	N	N	N	N	
525	3	N	N	N	N	Y	N	N	N	N	N	N	
526	1	N	N	N	Y	N	N	N	N	N	N	N	
527	3	N	N	N	N	N	Y	N	N	N	N	N	
528	1	N	N	N	N	Y	N	N	N	N	N	N	
529	3	N	Y	N	N	N	N	N	N	N	N	N	
530	3	N	N	N	N	Y	N	N	Y	N	N	N	
531	2	N	N	N	N	N	N	N	Y	N	N	N	
532	2	Y	N	N	N	N	N	N	N	N	N	N	
533	2	N	N	N	N	Y	N	N	N	N	N	N	
534	3	N	N	N	N	Y	N	N	N	N	N	N	
535	2	N	N	N	N	N	N	N	N	Y	N	N	
536	1	Y	N	N	N	N	N	N	N	N	N	N	
537	3	Y	N	Y	N	N	N	N	N	N	N	N	
538	3	N	N	N	N	Y	N	N	N	N	N	N	
539	2	Y	N	N	N	N	N	N	N	Y	N	N	
540	5	N	N	N	N	N	N	N	N	N	N	N	
541	1	N	Y	N	N	N	N	N	N	N	N	N	
542	2	N	N	Y	N	N	N	N	N	N	N	N	
543	3	N	N	N	N	Y	N	N	N	N	N	N	
544	2	N	N	N	N	N	Y	N	N	N	N	N	

ID	MEDEDUCA	WESTMED	CTM	WCM	PREVENT	NURSING	MEDLAB	ORAL	PHARM	GPAE	REHAB	HEALTHMA	OTHERAE
545	2	Y	N	N	N	N	N	N	N	N	N	N	
546	2	Y	N	N	N	N	N	N	N	N	N	Y	
547	2	Y	N	N	N	N	N	N	N	N	N	N	
548	1	N	Y	N	N	N	N	N	N	N	N	N	
549	2	Y	N	N	N	N	N	N	N	N	N	N	
550	2	N	Y	N	N	N	N	N	N	N	N	N	
551	1	N	Y	N	N	N	N	N	N	N	N	N	
552	1												
553	1	N	Y	N	N	N	N	N	N	N	N	N	
554	3	Y											
555	2					Y							
556	2					Y							
557	1				Y	Y							
558	3		Y										
559	2	N	N	N	N	Y	N	N	N	N	N	N	
560	1	N	N	N	N	N	N	N	N	Y	N	N	
561	2	N	N	Y	N	N	N	N	N	N	N	N	
562	2	N	N	N	N	N	N	N	N	N	N	Y	
563	2	N	N	N	N	Y	N	N	N	N	N	N	
564	2	N	N	N	N	Y	N	N	N	N	N	N	
565	1												
566	2	N	N	N	N	Y	N	N	N	N	N	N	
567	2	Y	N	N	N	N	N	N	N	N	N	N	
568	2	N	N	N	N	Y	N	N	N	N	N	N	
569	3	Y	N	N	N	N	N	N	N	N	N	N	
570	2	N	N	N	N	Y	N	N	N	N	N	Y	
571	2	N	N	N	N	N	N	N	N	N	Y	N	
572	3	Y	N	N	N	Y	Y	N	N	N	N	N	
573	1	N	N	N	Y	Y	N	N	N	N	N	N	
574	3	Y	N	N	N	N	N	N	N	N	N	N	
575	2	N	Y	N	N	N	N	N	N	N	Y	N	
576	3	Y	N	N	N	N	N	N	N	N	N	N	
577	3	Y	N	N	N	N	N	N	N	N	N	N	
578	1	Y	N	N	N	N	N	N	N	N	N	N	

ID	MEDEDUCA	WESTMED	CTM	WCM	PREVENT	NURSING	MEDLAB	ORAL	PHARM	GPAE	REHAB	HEALTHMA	OTHERAE
579	2	N	N	N	Y	N	N	N	N	N	N	N	
580	2								Y				
581	3								Y				
582	3	Y											
583	3	Y		Y									
584	3				Y								
585	3							Y					
586	2					Y							
587	2					Y							
588	2					Y							
589	3					Y							
590	2						Y						
591	3	Y											
592	5												
593	2					Y							
594	1									Y			
595	2					Y							
596	3			Y									
597	1								Y				
598	2					Y							
599	2			Y									
600	2					Y							
601	2					Y							
602	2	Y											
603	3					Y							
604	3	Y											
605	2					Y							
606	3	Y											
607	3					Y							
608	3					Y							
609	2					Y							
610	2					Y							
611	1					Y							
612	3	Y								Y			

ID	MEDEDUCA	WESTMED	CTM	WCM	PREVENT	NURSING	MEDLAB	ORAL	PHARM	GPAE	REHAB	HEALTHMA	OTHERAE
613	3	Y											
614	1	Y											
615	2		Y							Y			
616	3					Y							
617	3	Y								Y			
618	3					Y							
619	3	Y											
620	1	N	N	N	N	Y	N	N	N	N	N	N	
621	5												
622	2	Y	N	N	N	N	N	N	N	N	N	N	
623	3	N	Y	N	N	N	N	N	N	N	N	N	
624	2	N	N	N	N	Y	N	N	N	N	N	N	
625	1	N	N	N	N	Y	N	N	N	N	N	N	
626	3												
627	2	N	N	N	N	Y	N	N	N	N	N	N	
628	1												
629	3	Y	N	N	N	N	N	N	N	N	N	N	
630	1	N	N	N	N	Y	N	N	N	N	N	N	
631	1	N	N	N	N	Y	N	N	N	N	N	N	
632	1	N	N	N	N	Y	N	N	N	N	N	N	
633	1	Y	N	N	N	N	N	N	N	N	N	N	
634	3	Y	N	N	N	N	N	N	N	N	N	N	
635	2	Y	N	N	N	N	N	N	N	N	N	N	
636	2	Y	N	N	N	N	N	Y	N	N	N	N	
637	2	N	Y	N	N	N	N	N	N	Y	N	N	
638	3	Y	N	N	N	N	N	N	N	N	N	N	
639	2	Y	N	Y	N	N	N	N	N	Y	N	N	
640	2	N	Y	Y	N	N	N	N	N	N	Y	N	
641	2	Y	N	N	N	N	N	N	N	Y	N	N	
642	2	N	N	N	N	N	Y	N	N	N	N	N	
643	3	N	Y	N	N	N	N	N	N	Y	N	N	
644	3	Y	N	N	N	N	N	N	N	N	N	N	
645	2	Y	N	N	N	N	N	N	N	N	N	N	
646	2	Y	N	N	N	N	Y	N	N	N	N	N	

ID	MEDEDUCA	WESTMED	CTM	WCM	PREVENT	NURSING	MEDLAB	ORAL	PHARM	GPAE	REHAB	HEALTHMA	OTHERAE
647	3	Y	N	N	N	N	N	N	N	Y	N	N	
648	1	N	N	N	N	Y	N	N	N	N	N	N	
649	2	N	N	N	N	Y	N	N	N	N	N	N	
650	2	N	N	N	N	Y	N	N	N	N	N	N	
651	2	N	N	N	N	Y	N	N	N	N	N	N	
652	2	N	N	N	N	Y	N	N	N	N	N	N	
653	1	N	N	N	N	Y	N	N	N	Y	N	N	
654	3	Y	N	Y	N	N	N	N	N	N	N	N	
655	3	N	Y	N	N	N	N	N	N	N	N	N	
656	3	Y	N	N	N	N	N	N	N	N	N	N	

ID	genjs_1	LEAVE_1	INCM_1	BENEFIT_1	LRN_1	CAREER_1	PEERRELA_	RESPECTS_	SECURE_1	PARTIRES_	HONOR_1	INCOME5	BENEFIT5
1	3	4	3	3	3	3	5	3	4	4	3	4.5	4.5
2	3	2	3	4	4	3	4	3	4	2	3	3.5	3.5
3	3	2	3	3	4	3	4	4	3	3	3	4	3
4	4	3	3	3	4	3	5	4	4	4	3	5	4.5
5	4	3	3	4	4	4	5	5	4	4	4	1.5	1.5
6	3	2	2	2	4	3	4	4	3	1	5	2.5	3.5
7	4	3	3	3	3	3	4	5	4	3	4	4	2.5
8	5	1	4	4	4	5	5	5	4	5	5	5	4.5
9	4	3	1	2	3	4	4	4	4	4	4	3.5	4
10	3	3	2	2	2	1	4	4	3	2	3	5	5
11	4	2	3	3	3	3	5	4	4	2	3	5	5
12	4	2	4	4	4	4	5	4	4	4	5	4	4
13	4	2	2	3	3	3	4	4	4	3	3	4.5	4.5
14	3	2	3	4	4	3	4	3	4	2	3	2.5	2
15	3	2	2	1	5	1	4	4	3	1	5	5	5
16	3	2	2	3	3	2	2	4	3	2	3	4	4
17	4	3	1	3	4	3	4	4	4	4	4	4	3
18	3	2	3	3	4	3	4	4	3	3	3	3.5	3
19	2	3	2	3	2	3	4	4	4	4	3	2	2
20	4	2	2	3	3	3	4	5	3	4	3	5	5
21	3	3	3	2	3	2	5	3	3	2	2	1	1
22	3	3	3	1	3	2	5	3	3	2	2	2	1
23	4	4	4	4	4	4	4	4	4	4	4	3	3
24	4	4	4	4	4	4	4	4	4	4	4	4	2.5
25	3	3	2	2	2	2	4	4	4	3	3	5	5
26	3	2	2	2	4	3	4	4	4	2	3	3	4.5
27	3	4	2	3	3	2	4	3	3	4	3	5	5
28	3	3	3	2	3	2	4	3	2	3	3	5	5
29	3	2	3	3	3	2	4	4	5	3	4	3	3
30	3	3	2	3	3	3	4	2	4	1	3	5	5
31	3	4	4	3	3	3	3	3	4	4	3	4.5	4
32	4	2	3	3	3	3	4	3	3	4	3	3	3
33	4	1	1	3	3	3	4	4	4	3	3	4	4.5
34	4	2	3	3	4	3	4	4	4	3	4	5	5

ID	genjs_1	LEAVE_1	INCM_1	BENEFIT_1	LRN_1	CAREER_1	PEERRELA_	RESPECTS_	SECURE_1	PARTIRES_	HONOR_1	INCOME5	BENEFIT5
35	4	2	2	3	3	3	4	4	3	4	4	5	5
36	3	3	3	2	3	3	4	3	3	4	3	5	5
37	3	3	3	3	3	3	3	3	4	4	3	3	2.5
38	4	3	3	3	5	4	5	4	4	4	4	3.5	3.5
39	3	2	3	3	3	3	4	3	4	4	3	4.5	5
40	4	3	3	4	3	3	4	4	3	4	3	4	4
41	4	2	3	4	4	3	4	4	4	3	3	5	5
42	3	3	3	3	3	4	4	4	4	4	4	5	5
43	4	2	3	4	4	4	5	4	4	4	4	5	5
44	4	3	2	3	3	3	4	4	4	3	3	3	2.5
45	3	4	1	1	1	1	5	1	2	1	3	4	2.5
46	4	2	3	3	3	3	4	4	3	4	3	4.5	4.5
47	3	3	3	3	3	2	4	3	3	3	3	5	5
48	4	1	3	1	4	3	5	5	4	1	3	5	5
49	5	1	4	5	4	3	4	4	3	4	3	4	5
50	5	2	3	5	4	3	5	4	5	4	4	5	5
51	4	3	3	3	3	3	4	4	3	3	4	5	4.5
52	3	3	3	3	2	3	4	3	4	3	3	5	5
53	4	3	3	3	4	3	4	4	4	2	4	5	4.5
54	4	2	3	3	4	3	4	4	4	4	3	3.5	2.5
55	4	3	3	4	4	3	5	5	4	5	4	5	5
56	4	2	3	3	3	3	3	4	4	4	3	3.5	3.5
57	4	2	3	3	3	3	4	4	4	3	3	4.5	4.5
58	4	4	3	2	4	3	5	4	4	5	3	3	3.5
59	3	2	2	3	3	3	4	3	4	3	3	2.5	1.5
60	2	2	2	3	2	3	4	4	5	2	2	5	4
61	3	2	3	3	3	3	4	4	4	4	4	5	5
62	4	3	3	3	3	3	5	3	3	4	4	5	4.5
63	4	3	3	3	3	3	4	4	4	4	4	4	5
64	4	2	3	3	5	4	4	4	4	4	3	2.5	2.5
65	3	3	3	3	3	3	4	3	3	2	3	0.5	1
66	4	3	3	4	4	3	5	4	4	4	4	4.5	4.5
67	5	1	3	4	4	4	5	5	4	5	5	4	3.5
68	4	2	4	3	3	2	5	5	5	2	3	3	3.5

ID	genjs_1	LEAVE_1	INCM_1	BENEFIT_1	LRN_1	CAREER_1	PEERRELA_	RESPECTS_	SECURE_1	PARTIRES_	HONOR_1	INCOME5	BENEFIT5
69	3	2	2	2	4	2	4	5	3	3	4	4	5
70	4	1	3	2	2	2	5	5	3	4	2	3.5	3.5
71	4	4	3	3	4	3	4	4	3	4	3	3	3.5
72	3	3	3	4	3	3	4	3	3	4	3	5	4.5
73	3	2	1	3	2	1	4	3	3	3	3	2.5	2.5
74	3	2	2	3	3	3	4	3	4	2	3	3.5	2
75	3	1	2	3	2	3	4	3	4	2	3	3.5	2
76	4	3	2	3	2	3	4	3	4	3	3	3	2.5
77	4	1	1	3	4	3	5	5	4	2	4	3	1.5
78	4	2	2	2	3	3	4	3	4	4	4	3.5	2.5
79	3	2	3	3	3	3	5	5	4	3	3	5	4
80	3	3	3	3	2	3	5	2	3	3	3	1	0.5
81	5	1	3	4	5	3	5	4	4	5	5	3	2
82	4	2	3	4	4	4	5	4	4	5	4	3	3
83	3	3	3	3	3	2	5	3	4	2	3	5	5
84	3	5	3	3	3	2	5	3	3	4	3	4	4
85	5	3	4	4	5	4	5	3	5	5	4	5	4
86	3	2	3	4	3	4	5	3	4	3	4	4	4
87	3	3	3	2	3	3	4	4	4	3	3	4	4
88	3	2	3	2	3	2	4	4	4	2	3	4	4
89	4	2	2	3	3	3	4	4	4	2	3	2.5	3.5
90	3	3	3	3	3	3	4	4	3	1	1	4	4
91	3	3	2	3	3	2	4	4	4	2	3	5	4.5
92	3	4	3	3	4	2	4	4	4	4	3	4	5
93	3	3	2	3	4	3	4	3	3	2	3	4.5	3.5
94	3	2	1	2	4	3	4	3	2	3	3	5	0.5
95	4	2	2	4	4	2	5	5	3	2	4	3.5	4.5
96	4	1	3	4	4	3	4	4	4	4	3	3.5	1.5
97	4	3	2	2	3	3	5	3	3	3	3	4	4
98	3	3	2	3	3	3	4	3	3	2	3	5	5
99	4	2	3	3	4	3	4	4	4	4	4	5	4.5
100	3	5	2	3	3	1	4	4	1	1	3	5	5
101	3	4	3	2	4	3	5	3	2	2	3	5	5
102	4	2	3	2	2	3	5	4	4	4	4	4.5	4.5

ID	genjs_1	LEAVE_1	INCM_1	BENEFIT_1	LRN_1	CAREER_1	PEERRELA_	RESPECTS_	SECURE_1	PARTIRES_	HONOR_1	INCOME5	BENEFIT5
103	4	2	2	3	3	2	4	4	4	4	3	5	5
104	4	2	3	3	4	3	4	4	4	4	3	2.5	3
105	3	3	2	3	3	2	4	4	2	2	3	3	3
106	4	2	3	4	4	4	4	4	4	3	4	5	4
107	4	2	3	4	4	4	5	3	4	4	4	4.5	4.5
108	3	3	3	3	3	3	5	3	4	3	3	4	4
109	3	3	3	2	3	3	4	4	4	3	3	4	4
110	3	3	1	1	3	2	5	3	4	4	3	5	5
111	3	2	3	3	5	2	4	4	4	4	3	4.5	4
112	4	3	3	4	4	4	4	4	4	5	4	4.5	2
113	4	3	2	3	5	2	4	5	4	3	4	5	4.5
114	4	1	2	3	4	4	5	4	4	4	4	4.5	5
115	3	3	2	3	3	2	5	4	4	4	3	5	5
116	3	3	2	3	4	2	4	3	4	2	3	5	4.5
117	3	2	2	4	5	3	5	4	4	3	4	4	3.5
118	3	2	2	3	3	1	4	3	3	2	3	3.5	1.5
119	3	3	2	3	4	2	4	3	4	2	3	5	4.5
120	3	3	2	3	4	2	5	4	4	4	3	5	4.5
121	2	3	2	3	3	3	4	3	2	3	3	3.5	2
122	3	3	2	3	3	2	5	3	3	3	3	5	4
123	3	3	2	3	4	2	5	4	4	4	3	4	4.5
124	3	4	3	3	3	2	4	4	3	5	3	1.5	1.5
125	5	2	3	4	4	3	4	4	2	4	5	4.5	4.5
126	4	1	3	4	3	3	4	4	4	4	3	3.5	3.5
127	3	3	3	3	3	3	5	3	4	4	3	4.5	5
128	4	4	3	4	4	4	4	4	4	4	4	4.5	4
129	3	3	3	3	5	3	4	4	2	4	4	3.5	4.5
130	4	2	3	4	4	5	5	5	4	5	4	3	3
131	3	2	2	3	4	3	4	4	2	4	3	5	4
132	4	2	3	2	4	2	5	4	4	4	3	5	5
133	4	2	3	2	4	2	5	4	4	4	3	5	5
134	4	2	3	2	4	2	5	4	4	4	3	4.5	5
135	4	3	3	4	4	2	4	4	4	5	3	5	3.5
136	3	4	2	3	3	2	4	4	4	3	3	5	4.5

ID	genjs_1	LEAVE_1	INCM_1	BENEFIT_1	LRN_1	CAREER_1	PEERRELA_	RESPECTS_	SECURE_1	PARTIRES_	HONOR_1	INCOME5	BENEFIT5
137	4	4	3	4	3	3	4	4	4	4	3	5	4.5
138	3	3	3	2	2	2	5	3	4	2	3	5	5
139	4	2	2	3	3	3	4	4	4	4	4	5	4.5
140	4	2	3	2	3	1	4	4	4	4	3	5	5
141	2	4	3	2	2	2	4	4	4	1	2	1	0.5
142	3	2	3	3	3	3	4	4	3	3	3	1	1
143	4	3	2	4	2	4	5	4	4	4	4	5	5
144	3	3	2	2	3	1	5	4	3	2	3	4.5	4
145	4	2	2	3	5	3	5	5	4	4	4	4	4.5
146	4	3	2	3	3	3	5	3	4	4	3	4.5	4.5
147	3	3	2	2	3	1	5	4	2	2	3	5	4
148	5	2	2	5	4	4	5	5	5	5	4	4	4
149	4	1	3	3	3	3	5	5	4	4	3	4	2.5
150	3	5	3	3	4	4	5	5	4	4	3	4	3.5
151	3	3	2	3	4	3	5	3	4	2	4	4	4
152	4	2	2	4	3	3	5	5	3	4	4	4	4
153	3	5	2	4	4	3	5	4	5	5	3	3	2.5
154	3	3	3	4	3	3	4	4	4	4	3	5	5
155	3	2	3	2	2	1	4	4	4	2	2	2.5	3.5
156	3	2	4	3	3	3	5	3	5	4	2	5	2.5
157	3	2	3	4	4	1	4	4	4	4	4	2.5	2.5
158	4	2	3	4	4	4	4	4	4	4	4	4	4
159	4	4	1	4	4	3	4	5	5	5	4	2.5	4
160	4	3	3	4	5	4	5	5	4	4	3	4.5	3.5
161	4	2	3	4	4	4	4	4	4	4	4	3	4
162	3	3	3	2	4	3	4	4	4	2	3	4.5	3.5
163	4	2	3	4	4	4	4	4	4	4	4	1.5	0.5
164	3	2	3	2	2	2	4	4	4	2	3	3	3
165	3	2	3	2	2	1	3	3	1	2	2	3	0.5
166	3	3	3	3	3	3	4	4	2	3	3	4	1
167	3	2	1	3	3	2	4	4	3	1	3	3.5	2.5
168	3	4	3	3	3	3	4	4	4	4	3	3.5	3.5
169	4	2	3	4	3	3	4	3	4	4	3	4	4
170	4	2	3	3	3	3	5	3	4	4	3	4.5	2.5

ID	genjs_1	LEAVE_1	INCM_1	BENEFIT_1	LRN_1	CAREER_1	PEERRELA_	RESPECTS_	SECURE_1	PARTIRES_	HONOR_1	INCOME5	BENEFIT5
171	4	1	3	4	4	3	5	4	4	4	4	5	5
172	3	3	2	1	3	3	4	4	4	1	1	2	3
173	3	2	5	4	4	4	5	4	3	5	4	2.5	3.5
174	3	3	3	2	4	3	5	4	3	3	4	4.5	4
175	4	3	4	4	5	3	4	4	3	4	4	4	4
176	1	4	3	1	3	1	3	1	1	1	1	4.5	4.5
177	2	5	1	2	2	2	5	3	2	2	2	4.5	3.5
178	3	3	2	2	3	2	5	4	3	2	2	2	0.5
179	2	4	3	2	3	2	4	4	2	2	3	2.5	1.5
180	3	3	3	2	3	4	1	4	3	3	3	4	4
181	4	3	3	4	5	3	4	4	4	4	4	4	4.5
182	3	3	3	3	4	2	4	4	3	4	3	4	4.5
183	1	3	5	4	4	4	5	4	4	1	4	3.5	4
184	3	2	3	3	3	3	4	4	2	2	3	3.5	3.5
185	3	3	3	3	3	3	3	3	2	2	3	5	5
186	4	3	3	3	3	3	3	3	4	4	3	4.5	4.5
187	2	4	3	1	2	1	5	5	2	2	2	1	0.5
188	3	3	3	3	3	3	4	4	3	3	3	4.5	4
189	4	2	3	3	3	3	4	3	3	3	3	2	3
190	4	3	3	2	2	1	4	4	3	4	3	4	1
191	3	4	1	2	4	1	5	3	4	2	2	3.5	4.5
192	3	3	2	3	3	2	5	4	4	3	3	2.5	2.5
193	4	2	3	3	4	4	4	4	4	4	4	4.5	4
194	4	3	2	3	1	2	4	4	4	2	3	5	4
195	2	3	1	1	2	1	4	4	3	1	3	5	5
196	4	2	3	3	3	2	4	4	4	2	3	4	4.5
197	3	4	2	3	4	1	5	5	4	3	2	4	1.5
198	2	3	2	2	2	1	4	3	4	2	2	5	3.5
199	3	1	3	3	3	3	5	4	4	1	3	2	3
200	3	4	1	2	2	4	5	5	4	2	3	5	5
201	3	2	2	2	3	3	5	4	4	3	3	4.5	5
202	4	1	4	4	4	4	4	4	4	5	4	4	4
203	4	3	2	4	3	3	4	3	3	3	4	3	3
204	3	3	3	4	3	3	5	3	3	3	3	2	1

ID	genjs_1	LEAVE_1	INCM_1	BENEFIT_1	LRN_1	CAREER_1	PEERRELA_	RESPECTS_	SECURE_1	PARTIRES_	HONOR_1	INCOME5	BENEFIT5
205	4	3	3	3	4	5	4	4	4	4	4	2.5	1
206	4	1	3	4	4	3	5	4	5	4	4	2.5	2.5
207	4	3	3	3	4	3	4	4	4	5	4	4.5	4
208	3	3	3	1	1	1	5	3	2	3	1	5	4.5
209	4	2	3	3	3	3	4	3	3	4	3	3.5	4
210	3	3	1	3	4	1	4	5	4	4	2	4.5	5
211	4	2	1	3	5	3	5	5	4	4	4	3	4
212	3	2	3	2	2	2	4	4	2	3	2	2.5	0.5
213	4	1	3	4	3	4	5	5	2	4	5	2.5	4
214	3	1	5	4	3	3	3	4	4	4	4	3	2.5
215	4	3	3	4	4	3	4	4	4	4	4	4	4
216	4	3	2	4	3	3	4	4	4	4	4	2.5	1
217	4	1	3	4	3	3	5	4	4	3	3	3.5	2.5
218	3	4	3	3	3	3	4	3	4	4	3	4	3
219	2	4	3	1	2	1	5	5	4	1	2	5	4
220	4	1	2	5	3	3	5	4	4	4	4	5	3.5
221	3	2	3	3	3	3	4	3	4	3	3	5	4.5
222	3	2	2	2	2	3	5	4	4	3	3	5	5
223	2	4	3	1	2	1	4	4	4	1	2	4	4.5
224	3	3	3	1	3	1	4	4	4	1	2	4	4
225	4	3	1	3	4	3	4	3	4	4	3	5	5
226	3	1	1	2	2	2	4	3	2	3	2	5	5
227	3	3	4	3	3	1	4	3	3	3	3	0.5	1.5
228	3	3	2	1	3	1	5	4	4	2	3	5	4
229	4	2	2	3	4	2	5	4	4	2	3	4.5	4.5
230	4	2	2	3	3	3	4	3	4	4	4	4	4
231	3	2	2	3	1	3	4	4	3	4	3	5	4
232	4	2	2	2	4	3	5	4	4	4	3	4	3.5
233	3	2	2	3	2	2	5	4	3	4	3	4.5	3.5
234	3	2	2	2	3	3	4	4	2	2	3	4.5	5
235	3	4	1	3	5	1	5	4	2	2	1	5	5
236	5	1	3	5	5	4	5	4	4	5	5	3.5	1
237	2	3	1	2	3	1	5	4	3	2	2	5	5
238	5	2	3	4	4	4	5	4	4	5	4	4.5	4.5

ID	genjs_1	LEAVE_1	INCM_1	BENEFIT_1	LRN_1	CAREER_1	PEERRELA_	RESPECTS_	SECURE_1	PARTIRES_	HONOR_1	INCOME5	BENEFIT5
239	3	4	2	1	3	3	4	4	2	4	2	3	2.5
240	3	3	1	2	3	1	5	3	3	1	1	3	3.5
241	2	4	3	2	2	1	4	4	3	1	2	0.5	0.5
242	3	3	3	3	2	1	5	4	2	2	3	3.5	2.5
243	4	4	1	2	3	1	5	4	3	2	2	5	5
244	1	4	2	1	3	1	4	3	3	2	2	4	4
245	3	3	3	3	3	3	5	4	3	2	3	5	5
246	3	3	4	3	3	2	4	3	3	3	3	5	5
247	4	4	3	3	3	2	4	4	4	3	2	5	5
248	3	4	2	2	3	2	4	3	3	4	3	5	5
249	4	2	3	3	4	3	5	4	4	4	4	3	3
250	4	2	3	3	3	3	3	3	4	4	3	4	4
251	3	2	3	3	4	1	5	5	3	2	3	4	4
252	3	2	3	3	4	1	5	5	3	2	3	4	4
253	4	2	5	1	4	3	4	4	4	4	4	3.5	3.5
254	4	2	3	3	3	3	3	4	4	4	3	3	3.5
255	4	3	3	2	3	3	4	4	3	2	2	4.5	3
256	5	2	3	3	4	4	4	3	4	4	3	5	5
257	3	2	3	3	3	3	4	4	4	3	3	4	4
258	3	3	3	5	4	4	3	3	4	4	3	5	5
259	5	1	3	5	4	4	4	5	4	4	4	4.5	4
260	4	2	3	4	4	4	5	2	4	4	4	4.5	4
261	2	2	2	3	3	3	3	3	4	4	3	5	5
262	3	3	3	3	3	3	4	2	3	3	3	4	4
263	3	2	2	4	4	3	5	4	4	4	4	5	4.5
264	5	1	2	5	4	4	4	5	4	4	4	5	5
265	3	3	3	3	3	3	4	2	4	2	3	5	4.5
266	4	2	3	3	4	4	4	4	4	4	4	4.5	4
267	3	2	1	3	3	3	5	4	4	2	4	5	5
268	4	2	3	3	3	3	4	3	4	3	3	5	3.5
269	3	3	1	3	4	2	4	4	4	2	3	5	5
270	3	3	1	3	4	2	4	4	4	2	3	3.5	4
271	3	3	3	4	3	3	4	4	4	2	3	5	5
272	3	1	2	3	3	3	4	4	3	3	3	2.5	2.5

ID	genjs_1	LEAVE_1	INCM_1	BENEFIT_1	LRN_1	CAREER_1	PEERRELA_	RESPECTS_	SECURE_1	PARTIRES_	HONOR_1	INCOME5	BENEFIT5
273	4	2	3	3	4	3	5	4	4	4	2	4	4
274	5	1	3	4	4	4	5	4	4	5	4	4.5	5
275	4	2	2	3	3	3	4	4	4	4	4	4.5	5
276	4	1	3	4	4	3	4	4	4	4	3	2.5	4
277	3	2	2	3	4	3	4	3	5	2	3	5	5
278	3	2	2	3	3	3	4	4	5	2	3	5	5
279	3	1	2	3	3	2	4	4	2	3	3	4.5	4.5
280	4	2	3	4	4	4	4	3	4	4	4	4.5	4
281	5	1	2	5	4	4	4	5	4	4	4	5	4.5
282	3	3	3	2	3	1	5	4	4	2	3	5	5
283	4	1	2	5	3	3	5	3	4	4	4	5	5
284	3	2	3	2	3	1	5	3	4	2	3	4	4
285	4	1	3	3	3	2	5	4	4	4	4	3	3
286	5	2	3	4	5	5	5	5	5	5	5	4	4
287	4	2	3	3	3	3	5	3	4	4	3	3	3
288	5	1	3	4	5	5	5	5	5	5	5	4	4
289	3	2	4	3	3	3	4	2	4	2	3	2.5	2.5
290	4	3	3	4	4	3	4	3	4	5	4	2.5	4
291	3	3	3	3	2	2	4	2	4	3	3	5	3.5
292	4	2	3	3	3	3	5	4	4	4	3	4	4
293	3	2	3	4	4	2	5	4	4	5	4	3.5	2
294	4	2	2	3	3	3	4	4	4	4	3	3	3.5
295	4	1	3	4	3	1	5	3	4	3	3	4	4
296	5	2	3	4	4	4	5	3	5	5	5	3.5	2.5
297	3	3	1	4	3	3	5	4	4	3	3	3	3
298	3	3	3	3	3	3	4	2	3	3	3	4	4
299	4	3	2	3	3	3	4	4	4	4	3	5	5
300	3	2	3	3	3	2	4	3	4	2	3	3.5	3.5
301	5	1	2	5	4	4	4	4	4	5	4	5	4
302	4	2	2	3	4	3	5	3	3	4	4	4.5	4.5
303	4	2	2	4	4	4	4	4	4	4	4	4.5	4.5
304	3	2	2	3	3	3	4	3	3	4	4	5	5
305	4	2	2	4	4	4	4	4	4	5	4	5	5
306	4	2	2	3	3	3	5	4	4	3	3	4.5	4.5

ID	genjs_1	LEAVE_1	INCM_1	BENEFIT_1	LRN_1	CAREER_1	PEERRELA_	RESPECTS_	SECURE_1	PARTIRES_	HONOR_1	INCOME5	BENEFIT5
307	5	1	1	4	4	5	5	5	5	4	5	4	4
308	4	2	1	4	3	2	4	4	4	4	4	1.5	2.5
309	3	2	3	3	3	3	3	3	4	2	3	4.5	2
310	4	2	3	4	5	3	1	3	5	4	4	5	4.5
311	4	2	2	4	4	4	4	3	4	4	4	5	5
312	5	2	2	3	3	3	5	4	4	5	4	5	3.5
313	4	3	3	4	3	3	4	4	4	4	4	4.5	5
314	4	1	3	3	4	3	5	4	4	3	3	5	3.5
315	4	1	3	3	3	3	5	4	4	4	3	4	4
316	4	2	2	3	3	2	4	3	4	4	3	5	5
317	3	3	3	3	3	3	4	4	4	4	3	4	4
318	4	2	3	3	3	3	4	4	3	2	3	5	4
319	4	2	3	4	3	3	4	4	5	2	3	5	4
320	4	2	3	4	4	3	5	3	5	4	4	5	5
321	4	2	3	4	3	4	4	4	4	4	4	4	3.5
322	3	2	2	2	3	3	4	3	4	2	3	4	4
323	4	2	3	4	4	3	4	4	4	4	3	5	4
324	3	2	4	3	2	1	4	2	4	3	2	5	3.5
325	3	1	4	3	3	3	3	3	4	3	3	5	4
326	4	2	3	4	4	3	4	4	4	4	4	4	4
327	5	1	3	4	4	4	4	4	5	5	4	5	5
328	3	2	3	3	4	3	4	4	4	3	4	4.5	5
329	4	2	3	3	3	3	4	4	4	4	3	2.5	2
330	4	3	2	4	3	3	3	4	4	3	3	5	5
331	4	3	2	2	3	3	5	3	4	4	3	5	4.5
332	3	3	3	3	3	3	5	3	4	5	4	1	1
333	3	3	3	3	3	3	5	3	4	5	4	4.5	4.5
334	5	1	4	4	4	4	5	4	4	4	4	4	4
335	5	2	4	3	5	3	5	5	5	5	4	4.5	2.5
336	3	3	3	3	3	3	4	4	4	3	3	5	5
337	4	2	4	4	4	3	4	4	4	4	3	2.5	2.5
338	4	1	4	4	4	4	5	5	5	4	4	5	4
339	4	1	3	3	3	3	4	4	4	4	4	5	5
340	4	3	3	4	4	3	5	2	4	3	3	4.5	5

ID	genjs_1	LEAVE_1	INCM_1	BENEFIT_1	LRN_1	CAREER_1	PEERRELA_	RESPECTS_	SECURE_1	PARTIRES_	HONOR_1	INCOME5	BENEFIT5
341	3	3	2	3	1	2	4	3	4	3	3	5	5
342	4	3	3	4	4	3	4	4	4	3	4	4	4
343	3	3	2	4	2	2	4	4	4	4	3	5	5
344	3	4	3	4	4	3	4	4	4	4	3	2.5	2.5
345	3	3	4	2	2	3	4	4	4	3	4	4.5	4.5
346	3	2	4	3	3	2	4	4	3	3	3	4	4
347	3	3	3	3	4	3	4	3	4	3	3	5	5
348	4	2	3	4	4	4	5	5	4	4	4	5	4
349	4	2	3	4	4	4	5	5	4	4	3	5	5
350	3	2	3	2	3	3	3	3	3	2	3	3.5	0.5
351	3	2	3	3	3	2	4	3	2	3	4	1	1
352	4	2	3	3	4	4	4	4	4	3	3	2.5	3
353	3	2	3	2	4	2	4	4	4	2	3	3.5	3
354	4	3	2	4	4	2	4	3	2	5	4	4	4
355	5	1	3	3	5	4	5	5	4	4	4	4	3.5
356	3	2	3	2	2	2	3	3	4	2	2	4	4
357	4	2	3	3	4	3	4	4	4	3	3	1.5	2
358	3	1	3	3	3	3	4	4	4	2	3	4.5	4
359	3	2	2	2	3	2	4	5	4	3	2	5	5
360	3	3	2	2	3	2	3	4	3	2	3	4.5	4
361	4	4	1	2	3	2	4	4	3	2	2	5	5
362	3	3	5	1	3	2	3	4	2	2	3	5	4
363	2	4	2	3	3	1	4	3	3	3	3	5	5
364	4	2	3	3	4	4	4	4	4	5	5	5	5
365	4	2	2	2	2	3	4	4	4	4	3	5	5
366	4	2	1	4	5	4	4	4	5	4	5	5	4.5
367	4	1	1	4	4	4	4	5	4	4	4	5	4
368	4	3	3	3	3	4	4	4	4	3	4	5	5
369	3	2	3	3	2	3	4	4	2	3	3	2.5	5
370	4	2	4	4	4	4	5	4	4	5	4	1.5	2.5
371	4	2	4	4	4	3	5	5	4	4	4	5	5
372	4	2	4	4	4	4	5	4	4	5	5	4.5	4
373	3	3	3	3	4	3	4	4	3	4	3	2	1.5
374	3	5	1	4	5	3	5	5	4	1	4	5	5

ID	genjs_1	LEAVE_1	INCM_1	BENEFIT_1	LRN_1	CAREER_1	PEERRELA_	RESPECTS_	SECURE_1	PARTIRES_	HONOR_1	INCOME5	BENEFIT5
375	4	2	4	4	4	4	4	4	4	5	4	5	4
376	4	2	4	4	4	4	5	4	4	5	5	4.5	4
377	4	4	2	3	3	4	5	5	4	3	3	3.5	3
378	4	5	1	3	4	4	4	4	4	4	4	5	4
379	3	2	3	3	3	2	3	4	3	1	2	5	5
380	5	1	3	4	3	3	5	5	5	4	3	5	5
381	2	4	3	2	4	2	3	4	5	5	4	5	2
382	4	3	2	2	3	3	3	4	4	4	3	4	5
383	3	2	1	2	3	3	3	3	3	4	3	5	3.5
384	4	2	2	3	5	3	5	4	3	3	4	4.5	4
385	3	2	3	3	3	3	3	5	3	2	3	2.5	2
386	4	3	1	3	4	3	5	5	4	4	4	4	4
387	3	2	2	3	2	2	4	4	3	1	3	5	4
388	3	2	3	3	5	3	5	5	3	2	3	2.5	3
389	3	2	2	3	3	3	4	4	3	4	4	2.5	2.5
390	5	2	3	4	5	4	4	4	4	3	4	4.5	4.5
391	3	4	3	3	3	3	4	4	3	4	4	4	4.5
392	3	3	4	3	2	3	5	4	3	4	2	4.5	3
393	3	3	3	3	3	4	4	4	3	3	3	3.5	3.5
394	3	3	4	2	3	3	4	4	3	3	3	0.5	2
395	4	3	3	3	4	4	5	4	4	4	3	2.5	2
396	4	2	3	3	3	3	3	3	4	3	3	4	4
397	4	2	3	4	3	3	4	3	4	3	3	2.5	5
398	3	4	3	3	3	3	4	3	4	4	4	4.5	4.5
399	3	3	3	3	4	3	3	3	4	2	2	5	5
400	4	3	3	3	3	2	4	4	4	4	3	3.5	3.5
401	4	1	3	4	3	2	5	5	4	2	3	5	5
402	4	3	3	4	4	4	5	4	4	3	5	5	4.5
403	3	2	4	2	3	2	4	4	4	4	4	2.5	1.5
404	4	2	2	3	3	3	4	3	4	4	3	5	5
405	4	3	3	3	3	3	5	3	4	3	3	2	2.5
406	3	2	4	3	2	4	4	3	4	3	3	5	5
407	3	4	3	2	4	2	4	1	4	3	4	5	4.5
408	4	2	4	3	3	3	5	3	4	4	3	3	2

ID	genjs_1	LEAVE_1	INCM_1	BENEFIT_1	LRN_1	CAREER_1	PEERRELA_	RESPECTS_	SECURE_1	PARTIRES_	HONOR_1	INCOME5	BENEFIT5
409	3	3	3	3	3	3	4	4	4	3	3	4	4.5
410	4	1	3	4	3	2	5	5	4	2	3	5	5
411	4	2	2	4	4	3	5	4	5	4	4	4.5	4.5
412	2	3	2	3	3	3	5	4	3	4	3	5	5
413	5	2	4	4	4	3	5	5	4	4	4	3.5	3.5
414	3	4	3	3	4	3	4	4	3	3	3	5	5
415	3	3	2	3	4	3	4	3	4	4	3	5	5
416	4	1	5	4	4	4	5	4	4	4	4	4.5	5
417	5	1	1	4	3	4	5	4	4	5	4	5	5
418	5	1	2	4	4	4	4	4	4	4	4	4.5	4.5
419	5	1	3	5	5	5	5	5	4	5	5	4.5	4.5
420	4	1	3	3	4	3	5	4	4	4	4	2.5	2.5
421	3	4	3	3	2	2	3	4	2	4	3	5	4.5
422	4	2	3	3	4	2	4	4	4	4	4	4.5	4
423	4	1	3	4	2	3	5	5	4	4	4	4	4
424	3	3	3	4	3	3	3	3	3	3	3	4	4
425	2	3	2	2	3	1	5	4	3	2	2	5	5
426	4	2	3	3	3	3	4	4	5	4	3	3	1.5
427	4	2	3	4	4	4	5	4	4	5	4	4	5
428	4	2	3	4	4	4	4	4	4	4	4	4	4
429	5	1	3	4	4	5	4	4	4	4	4	5	5
430	5	2	3	3	3	3	5	4	5	4	4	4	4
431	4	1	3	4	4	4	4	4	4	4	4	3.5	3
432	4	2	3	3	3	3	4	4	4	2	3	4.5	4
433	3	1	2	4	4	1	4	4	3	4	3	1.5	2.5
434	4	3	3	4	4	3	4	4	4	4	3	3	3.5
435	4	2	2	4	5	3	5	5	4	4	4	5	5
436	4	2	2	3	4	4	4	3	4	2	4	3	3.5
437	4	3	3	4	4	3	4	4	4	3	3	4.5	4.5
438	4	2	2	3	4	5	5	4	4	4	3	4	4
439	4	2	2	4	4	3	5	5	2	4	4	5	5
440	4	2	2	4	4	4	4	4	4	4	4	3	2.5
441	4	3	4	3	3	2	4	4	3	2	3	3.5	3.5
442	4	3	1	3	4	3	4	4	3	4	4	4.5	4.5

ID	genjs_1	LEAVE_1	INCM_1	BENEFIT_1	LRN_1	CAREER_1	PEERRELA_	RESPECTS_	SECURE_1	PARTIRES_	HONOR_1	INCOME5	BENEFIT5
443	3	3	2	3	4	3	4	4	2	2	3	5	5
444	4	1	4	3	3	3	5	3	4	4	4	4	3
445	4	3	4	3	3	3	5	4	4	2	3	3.5	2.5
446	4	2	3	3	3	3	5	4	3	4	4	3	3.5
447	3	2	3	3	3	3	4	4	4	4	3	4	4.5
448	3	1	3	3	3	3	4	3	1	4	4	5	5
449	4	5	3	2	3	2	5	4	4	2	2	5	4.5
450	4	3	3	3	3	3	4	3	3	4	4	4	4.5
451	4	1	2	2	3	1	5	4	4	4	3	2.5	1
452	3	3	3	1	1	1	4	3	3	2	2	3.5	2.5
453	3	2	3	2	3	3	4	4	4	4	3	3	1.5
454	4	2	3	3	3	3	4	4	4	4	3	5	5
455	4	3	2	4	4	3	5	3	3	4	3	3	3.5
456	3	3	3	3	4	3	5	3	4	3	3	2.5	1
457	4	3	2	3	3	4	5	5	4	4	4	3	3
458	3	2	2	3	3	3	4	4	4	4	4	5	2.5
459	4	2	3	3	4	2	4	4	4	4	3	5	5
460	3	4	3	2	4	3	4	3	3	3	3	2	0.5
461	4	2	2	2	3	3	4	3	3	5	3	3	1.5
462	3	3	3	1	4	2	5	5	3	2	3	5	4
463	4	2	3	3	4	3	1	5	4	1	3	2.5	2.5
464	4	2	2	3	4	3	5	5	4	3	4	2.5	2.5
465	4	2	3	3	3	3	5	5	4	4	5	5	4.5
466	3	3	1	3	3	3	5	4	3	3	3	5	4
467	4	3	3	3	4	4	4	4	4	4	4	3.5	3.5
468	3	2	3	3	4	4	5	4	4	4	3	5	5
469	4	2	3	3	3	4	4	4	4	4	4	3	3
470	3	2	3	3	3	3	3	4	3	4	3	4	3.5
471	3	3	3	3	3	3	4	4	3	1	3	4.5	2.5
472	4	3	3	3	3	2	3	4	3	4	3	3.5	4
473	4	2	2	3	4	4	5	5	4	3	4	2.5	5
474	4	3	3	3	2	3	4	4	4	4	3	4.5	2.5
475	3	4	3	3	3	1	3	5	3	3	3	4.5	5
476	4	3	3	2	1	2	4	4	4	2	3	4	4

ID	genjs_1	LEAVE_1	INCM_1	BENEFIT_1	LRN_1	CAREER_1	PEERRELA_	RESPECTS_	SECURE_1	PARTIRES_	HONOR_1	INCOME5	BENEFIT5
477	3	4	3	1	3	3	5	4	3	3	3	2.5	1.5
478	5	1	3	3	4	3	4	4	4	4	3	5	4
479	3	2	3	3	3	3	4	4	3	2	3	4.5	4.5
480	4	1	2	2	5	4	5	5	2	5	4	3.5	5
481	4	3	2	5	3	3	4	4	2	2	3	5	5
482	5	1	2	3	4	3	4	4	4	4	3	4.5	4
483	5	4	1	1	3	1	5	4	3	3	2	5	4
484	3	3	3	2	3	3	4	4	3	3	3	1.5	0.5
485	5	4	1	1	3	2	4	4	3	4	2	3	3.5
486	4	2	2	3	3	2	4	3	2	4	4	5	5
487	4	4	3	2	2	1	5	4	3	2	3	4.5	3
488	3	4	1	2	4	1	4	4	3	3	2	0.5	0.5
489	3	2	2	2	3	3	4	4	4	3	2	5	5
490	4	3	2	3	3	3	4	4	3	2	3	5	5
491	3	2	2	2	3	3	4	4	4	1	2	5	5
492	5	4	1	3	4	3	3	4	5	4	3	5	5
493	4	3	3	3	2	1	4	4	4	4	3	3.5	3
494	4	2	4	4	4	3	3	3	3	4	4	4	2
495	3	4	2	1	2	1	4	1	1	4	3	5	5
496	4	2	4	3	3	4	4	4	4	4	4	3	3
497	3	2	3	4	3	3	3	3	3	2	3	1.5	2.5
498	4	3	3	4	4	4	5	4	4	4	4	4	3.5
499	5	3	2	3	3	4	5	5	4	4	4	3.5	4
500	4	2	2	4	4	4	4	4	4	2	4	4	4
501	3	2	1	2	3	1	4	4	4	3	1	5	5
502	4	3	3	3	3	4	5	4	4	4	4	5	5
503	3	3	1	1	3	2	5	5	2	3	3	3	4.5
504	3	3	3	3	3	2	4	3	4	3	3	5	4.5
505	4	2	1	3	4	2	5	4	3	4	4	4.5	5
506	4	4	1	2	3	1	4	4	4	2	3	5	5
507	4	2	3	3	3	3	4	3	3	4	4	5	5
508	3	3	3	3	3	3	3	3	2	1	4	2.5	2.5
509	3	3	2	2	3	2	4	4	4	2	3	5	5
510	3	3	2	2	2	2	4	4	3	4	3	5	5

ID	genjs_1	LEAVE_1	INCM_1	BENEFIT_1	LRN_1	CAREER_1	PEERRELA_	RESPECTS_	SECURE_1	PARTIRES_	HONOR_1	INCOME5	BENEFIT5
511	3	1	1	3	2	2	4	4	4	2	2	2.5	2.5
512	3	3	2	3	4	2	4	3	4	4	3	5	4
513	2	3	1	2	2	2	3	3	3	3	2	5	5
514	4	1	2	4	3	4	4	4	4	4	4	5	4.5
515	2	2	2	3	3	1	5	5	3	4	3	5	5
516	4	2	1	3	4	1	5	3	4	4	4	3.5	4
517	5	1	4	4	4	3	5	4	5	4	3	3	3
518	4	2	3	4	3	1	3	4	4	4	3	3.5	4
519	4	3	3	3	3	3	4	3	3	4	3	2.5	4
520	4	2	3	4	4	3	5	4	3	4	4	4	4.5
521	2	4	3	3	3	2	4	3	4	3	3	2.5	3
522	5	1	3	4	3	3	4	4	5	5	4	3.5	3
523	3	3	3	3	3	3	3	4	3	2	3	3	3
524	4	2	4	5	5	4	4	4	4	4	4	5	5
525	4	2	3	4	4	4	4	4	4	4	3	5	5
526	4	2	3	4	4	3	3	3	3	4	4	3.5	3.5
527	4	3	3	3	3	2	4	3	4	2	4	3.5	4
528	4	4	2	4	3	3	5	4	4	5	4	3	4
529	3	2	3	3	3	3	5	4	4	4	3	2.5	1.5
530	4	2	3	4	4	4	5	4	4	4	3	5	5
531	4	2	2	3	4	3	5	4	4	4	4	5	5
532	5	1	3	4	5	4	5	4	5	5	4	5	5
533	4	4	2	5	4	3	4	4	4	5	3	5	5
534	3	2	2	3	4	3	5	4	4	3	3	1.5	2.5
535	3	3	2	4	3	1	4	4	4	2	3	5	5
536	3	2	3	3	3	1	4	3	2	2	3	2.5	2.5
537	4	3	3	4	4	2	4	3	4	3	2	2.5	5
538	4	4	3	4	3	3	5	4	4	5	4	5	4.5
539	5	1	4	4	5	4	5	5	4	5	5	4.5	4.5
540	3	3	3	4	3	1	4	4	3	4	3	2.5	2.5
541	4	3	2	3	3	3	3	3	4	4	3	2.5	2.5
542	4	4	2	4	3	3	5	4	4	5	4	5	5
543	4	4	2	4	3	3	4	4	4	5	4	3.5	4
544	5	1	4	5	4	5	5	4	5	5	5	5	5

ID	genjs_1	LEAVE_1	INCM_1	BENEFIT_1	LRN_1	CAREER_1	PEERRELA_	RESPECTS_	SECURE_1	PARTIRES_	HONOR_1	INCOME5	BENEFIT5
545	5	1	4	4	4	3	5	4	4	5	4	5	5
546	3	3	3	3	3	1	5	3	4	3	2	3.5	3
547	4	2	3	3	4	3	4	4	4	4	3	2.5	2.5
548	3	3	4	4	4	2	4	4	3	2	3	4	4.5
549	5	3	3	3	4	4	5	4	4	4	5	5	4
550	3	3	3	3	3	3	3	3	4	4	3	3	3
551	4	3	2	3	3	3	3	3	4	4	3	4.5	4.5
552	3	2	3	4	3	3	4	4	4	3	4	2.5	2.5
553	4	2	3	3	3	3	4	4	4	4	3	3	3.5
554	4	2	3	3	3	3	4	3	2	4	3	4	4
555	4	2	3	3	4	3	4	3	3	3	4	0.5	0.5
556	4	2	3	4	4	3	5	3	4	4	3	2.5	2.5
557	4	1	2	4	4	4	4	4	3	4	4	2.5	2.5
558	3	4	3	3	3	1	5	5	2	1	1	0.5	0.5
559	2	4	3	2	3	1	4	3	1	3	3	1.5	1.5
560	3	4	1	1	3	3	5	5	3	4	3	1.5	0.5
561	4	2	2	3	4	3	4	4	3	4	2	1	0.5
562	3	3	4	4	3	3	5	4	4	2	3	4.5	4.5
563	3	3	1	4	3	3	4	3	4	3	3	4.5	4
564	3	3	3	3	3	3	4	3	4	4	3	3.5	4
565	4	2	3	4	4	3	5	5	3	2	4	4	4
566	3	4	3	3	4	4	4	5	3	4	4	3.5	3
567	4	2	3	4	4	3	4	4	3	4	3	4	4
568	2	5	3	2	1	3	3	3	3	3	2	5	5
569	3	3	3	4	3	3	4	4	4	4	3	5	5
570	4	4	2	5	4	3	4	4	4	3	4	4	3.5
571	3	3	3	3	3	3	4	3	4	3	3	5	5
572	5	2	3	5	4	3	5	5	4	5	4	2.5	4
573	3	1	3	3	3	3	3	3	3	3	3	1.5	2.5
574	4	2	1	3	3	4	5	4	3	4	2	5	2.5
575	3	3	2	3	3	3	4	4	3	2	3	5	5
576	4	2	3	3	4	3	4	4	4	5	4	5	5
577	4	2	3	5	4	4	5	4	5	4	5	4	4
578	3	3	3	3	4	3	4	4	4	4	3	2.5	2.5

ID	genjs_1	LEAVE_1	INCM_1	BENEFIT_1	LRN_1	CAREER_1	PEERRELA_	RESPECTS_	SECURE_1	PARTIRES_	HONOR_1	INCOME5	BENEFIT5
579	4	2	2	4	4	3	5	5	4	4	4	4.5	5
580	3	4	3	2	3	2	5	4	3	4	3	5	5
581	2	3	4	2	2	3	5	4	5	4	5	5	5
582	3	3	3	3	2	3	5	3	5	4	4	5	5
583	3	4	3	3	2	2	4	3	4	2	2	3	3
584	4	2	4	3	4	3	4	4	4	4	3	5	3
585	4	2	3	2	3	3	5	4	5	3	4	4.5	3.5
586	5	4	3	3	3	1	3	3	2	3	3	5	3
587	4	2	3	3	3	3	5	4	5	3	3	2	1.5
588	4	3	3	3	4	3	4	4	4	3	4	5	4.5
589	4	2	3	3	3	2	5	2	4	2	3	5	4.5
590	4	2	3	3	3	3	5	3	1	3	3	3.5	3
591	5	1	3	2	3	3	5	4	5	4	4	4.5	3
592	5	1	3	4	3	1	4	3	2	4	3	5	5
593	4	2	1	1	2	3	4	4	4	2	3	3.5	3.5
594	3	2	3	3	3	3	5	4	4	4	3	3.5	3
595	3	2	1	1	1	1	4	4	4	1	3	2.5	3.5
596	2	4	2	2	3	3	5	4	3	4	3	1	0.5
597	4	2	3	1	3	3	5	3	4	5	4	5	4.5
598	5	3	3	4	5	3	5	5	4	5	4	4	4
599	3	2	2	1	2	1	4	4	2	2	2	4.5	4.5
600	1	4	1	1	3	3	5	1	1	2	5	4.5	0.5
601	4	2	3	4	4	4	4	3	4	4	4	4	4
602	2	5	1	1	2	2	5	4	4	1	1	4.5	4.5
603	3	3	3	2	3	1	5	4	5	3	4	4	3.5
604	4	2	2	3	4	2	4	3	3	4	3	4.5	4.5
605	1	4	1	1	4	2	5	3	1	2	4	3	1
606	5	1	3	2	4	3	5	4	5	4	3	4.5	3
607	3	3	3	2	2	1	5	4	4	1	4	5	5
608	3	2	4	3	4	3	4	4	4	2	3	5	5
609	4	3	2	1	2	1	4	4	2	1	2	2.5	2.5
610	4	2	2	3	4	2	4	3	3	4	3	4.5	4.5
611	3	3	3	3	3	3	4	4	4	3	3	4.5	2.5
612	3	3	3	3	3	3	4	3	4	3	3	5	3.5

ID	genjs_1	LEAVE_1	INCM_1	BENEFIT_1	LRN_1	CAREER_1	PEERRELA_	RESPECTS_	SECURE_1	PARTIRES_	HONOR_1	INCOME5	BENEFIT5
613	4	3	3	3	5	3	5	4	4	2	4	4.5	5
614	4	3	3	3	3	1	4	4	2	2	3	2	1
615	3	2	4	3	4	2	4	4	2	2	3	5	5
616	4	2	2	3	3	3	5	5	4	3	3	3.5	2.5
617	3	1	3	3	3	2	3	4	3	2	3	2	2
618	3	2	3	3	3	4	4	4	4	4	3	4	4
619	3	3	3	3	3	3	4	4	4	3	3	2.5	2.5
620	5	2	2	4	3	3	5	5	4	4	4	4.5	4.5
621	4	3	4	4	5	3	5	4	4	4	5	4.5	4
622	3	4	3	2	3	2	4	4	2	3	3	3.5	1
623	3	4	3	2	1	1	4	3	4	2	2	5	5
624	3	3	1	2	3	3	4	4	1	4	2	4.5	3.5
625	4	4	3	3	4	3	5	3	4	2	3	0.5	1.5
626	3	4	3	4	3	3	4	4	4	5	3	3	3.5
627	4	3	3	3	3	3	4	4	4	2	3	5	5
628	4	4	3	2	4	3	4	4	4	4	2	4.5	4.5
629	3	3	2	2	3	1	4	3	3	4	3	4	4
630	4	4	4	4	3	3	4	4	4	5	3	4	5
631	3	3	3	2	2	3	3	5	4	3	3	4.5	5
632	4	3	3	2	4	3	5	4	4	4	2	2.5	5
633	4	2	4	4	4	3	4	4	4	4	3	4	2.5
634	2	2	3	4	3	3	4	4	4	3	4	4.5	4.5
635	3	2	3	1	2	2	4	3	4	3	2	5	4
636	3	2	2	1	3	3	5	4	4	3	3	0.5	0.5
637	4	3	2	4	4	3	4	4	4	4	4	3.5	3.5
638	3	2	4	2	3	2	3	4	4	5	3	3	2.5
639	2	3	3	2	3	2	4	3	4	2	3	2.5	2.5
640	5	2	2	2	2	1	4	4	4	5	3	5	5
641	4	1	3	3	3	3	4	3	3	4	4	2.5	1
642	2	3	4	2	4	2	4	4	5	2	3	5	5
643	3	3	3	2	3	2	4	4	3	2	3	4	3
644	3	2	3	2	3	2	3	4	4	3	3	4.5	5
645	3	3	3	3	3	2	4	3	3	3	3	4	1
646	3	3	3	3	3	3	4	3	4	3	3	4	3.5

ID	genjs_1	LEAVE_1	INCM_1	BENEFIT_1	LRN_1	CAREER_1	PEERRELA_	RESPECTS_	SECURE_1	PARTIRES_	HONOR_1	INCOME5	BENEFIT5
647	4	1	4	3	4	3	5	4	4	3	3	2.5	3
648	3	3	3	2	3	2	4	4	3	3	3	2.5	2.5
649	4	2	3	3	3	3	4	4	4	4	4	2.5	2.5
650	3	2	3	2	4	3	4	3	4	3	3	5	4
651	3	2	3	2	5	1	4	4	2	2	1	5	5
652	3	3	3	3	3	2	4	3	4	4	3	5	5
653	4	1	3	3	3	3	4	3	3	4	4	2.5	1.5
654	3	3	2	3	3	3	4	4	4	3	3	2.5	2.5
655	4	1	3	2	3	2	4	4	5	4	4	4.5	3.5
656	2	2	4	2	2	2	3	3	4	1	2	5	5

ID	LEARN5	CAREER5	PEERRELAT	RESPECT5	SECURE5	PARTICI5	HORNOR5	CAREERfit	INCOMEfit	LEARNfit	BENEFITfi	PARTfit	RESPECTfi
1	4.5	4	2.5	4	3.5	2.5	2.5	-1	-1.5	-1.5	-1.5	1.5	-1
2	4	3.5	2	3.5	2.5	3	2	-0.5	-0.5	0	0.5	-1	-0.5
3	3.5	4.5	4.5	5	1.5	3	2.5	-1.5	-1	0.5	0	0	-1
4	3.5	4.5	2	2	0.5	4.5	2	-1.5	-2	0.5	-1.5	-0.5	2
5	3.5	4.5	5	5	4	3	5	-0.5	1.5	0.5	2.5	1	0
6	4	2.5	2.5	2.5	2.5	2.5	3.5	0.5	-0.5	0	-1.5	-1.5	1.5
7	4	4	5	5	4	4	4	-1	-1	-1	0.5	-1	0
8	5	5	5	4.5	4.5	4.5	4.5	0	-1	-1	-0.5	0.5	0.5
9	4	4.5	5	3	1.5	3	3	-0.5	-2.5	-1	-2	1	1
10	5	4	4	2.5	2	4	5	-3	-3	-3	-3	-2	1.5
11	5	4.5	5	4	4	4	4	-1.5	-2	-2	-2	-2	0
12	3	3	5	5	5	2	4.5	1	0	1	0	2	-1
13	5	4	5	4	4.5	4.5	5	-1	-2.5	-2	-1.5	-1.5	0
14	2.5	3.5	3	3	3	2.5	2	-0.5	0.5	1.5	2	-0.5	0
15	4	2.5	2.5	2.5	2.5	2.5	3	-1.5	-3	1	-4	-1.5	1.5
16	4	4	5	5	5	3.5	5	-2	-2	-1	-1	-1.5	-1
17	3	2.5	5	5	2.5	4.5	4	0.5	-3	1	0	-0.5	-1
18	4	4	4	5	1.5	2.5	2.5	-1	-0.5	0	0	0.5	-1
19	2.5	3	3	4	3.5	2.5	3.5	0	0	-0.5	1	1.5	0
20	4	4.5	4	4.5	4	4	2.5	-1.5	-3	-1	-2	0	0.5
21	1	1	2.5	2.5	2	2.5	1	1	2	2	1	-0.5	0.5
22	2.5	2	2.5	2.5	3	2.5	1	0	1	0.5	0	-0.5	0.5
23	1.5	2.5	4.5	3.5	3.5	3	3	1.5	1	2.5	1	1	0.5
24	4.5	4.5	4.5	4.5	4	4.5	2.5	-0.5	0	-0.5	1.5	-0.5	-0.5
25	4	4	5	4.5	5	4.5	4	-2	-3	-2	-3	-1.5	-0.5
26	5	5	4.5	3	3	3.5	4	-2	-1	-1	-2.5	-1.5	1
27	4	5	4	4	4	3	4	-3	-3	-1	-2	1	-1
28	4.5	5	5	4.5	4	4	4	-3	-2	-1.5	-3	-1	-1.5
29	3.5	0.5	4	4.5	5	4	5	1.5	0	-0.5	0	-1	-0.5
30	3.5	4	4.5	5	5	5	5	-1	-3	-0.5	-2	-4	-3
31	4.5	4.5	4.5	4	5	4.5	5	-1.5	-0.5	-1.5	-1	-0.5	-1
32	2.5	2	3.5	3.5	3.5	2.5	3	1	0	0.5	0	1.5	-0.5
33	4.5	4.5	3.5	4	3	4	2	-1.5	-3	-1.5	-1.5	-1	0
34	4	3.5	5	3.5	3.5	3.5	3.5	-0.5	-2	0	-2	-0.5	0.5

ID	LEARN5	CAREER5	PEERRELAT	RESPECT5	SECURE5	PARTICI5	HORNOR5	CAREERfit	INCOMEfit	LEARNfit	BENEFITfi	PARTfit	RESPECTfi
35	4	4.5	5	5	5	4.5	4.5	-1.5	-3	-1	-2	-0.5	-1
36	3	1.5	3	3	2.5	2.5	1.5	1.5	-2	0	-3	1.5	0
37	3.5	4.5	4.5	3.5	4	4	3.5	-1.5	0	-0.5	0.5	0	-0.5
38	4.5	4.5	4.5	4.5	4.5	5	4.5	-0.5	-0.5	0.5	-0.5	-1	-0.5
39	3.5	3	4	5	5	4	3.5	0	-1.5	-0.5	-2	0	-2
40	3	3	4.5	5	4.5	2.5	5	0	-1	0	0	1.5	-1
41	3.5	3.5	4	4	4	3.5	4	-0.5	-2	0.5	-1	-0.5	0
42	4	3.5	3.5	5	5	5	5	0.5	-2	-1	-2	-1	-1
43	5	5	5	5	4	4	5	-1	-2	-1	-1	0	-1
44	2.5	3	3.5	4.5	3	1	4	0	-1	0.5	0.5	2	-0.5
45	3.5	4	5	5	4	4	4	-3	-3	-2.5	-1.5	-3	-4
46	4.5	4.5	4.5	4.5	4.5	4.5	4.5	-1.5	-1.5	-1.5	-1.5	-0.5	-0.5
47	3.5	2.5	2	3	4	2	2	-0.5	-2	-0.5	-2	1	0
48	3	3.5	3.5	2.5	2	3	3	-0.5	-2	1	-4	-2	2.5
49	4	2.5	5	4	4	3	3	0.5	0	0	0	1	0
50	3.5	5	2.5	4	3.5	3.5	4	-2	-2	0.5	0	0.5	0
51	4	4	2.5	3	4.5	3	1.5	-1	-2	-1	-1.5	0	1
52	5	4	4.5	3.5	3.5	4	5	-1	-2	-3	-2	-1	-0.5
53	4	4.5	3.5	4	3	3	3	-1.5	-2	0	-1.5	-1	0
54	3	2	5	5	4.5	3	3.5	1	-0.5	1	0.5	1	-1
55	4	4	5	5	5	4	4.5	-1	-2	0	-1	1	0
56	3	3.5	5	5	4	4.5	3.5	-0.5	-0.5	0	-0.5	-0.5	-1
57	4.5	2.5	3.5	3.5	4	4	4.5	0.5	-1.5	-1.5	-1.5	-1	0.5
58	2.5	4.5	5	5	3.5	3	4	-1.5	0	1.5	-1.5	2	-1
59	1.5	2.5	2.5	2.5	1.5	1.5	1.5	0.5	-0.5	1.5	1.5	1.5	0.5
60	3.5	3.5	3.5	3	2	3	2.5	-0.5	-3	-1.5	-1	-1	1
61	3.5	4.5	3.5	3	2.5	1.5	3	-1.5	-2	-0.5	-2	2.5	1
62	5	5	4.5	5	4.5	4	4.5	-2	-2	-2	-1.5	0	-2
63	4.5	5	4	5	4.5	4	4	-2	-1	-1.5	-2	0	-1
64	2.5	1.5	4.5	4.5	4.5	2.5	2.5	2.5	0.5	2.5	0.5	1.5	-0.5
65	1	1	2.5	3	3	2.5	2.5	2	2.5	2	2	-0.5	0
66	5	4	4.5	5	5	5	5	-1	-1.5	-1	-0.5	-1	-1
67	4.5	5	5	4.5	5	5	4	-1	-1	-0.5	0.5	0	0.5
68	3	4	5	5	5	3	4	-2	1	0	-0.5	-1	0

ID	LEARN5	CAREER5	PEERRELAT	RESPECT5	SECURE5	PARTICI5	HORNOR5	CAREERfit	INCOMEfit	LEARNfit	BENEFITfi	PARTfit	RESPECTfi
69	4.5	4	4	4	5	4.5	4.5	-2	-2	-0.5	-3	-1.5	1
70	4	3.5	0.5	5	3.5	4	3.5	-1.5	-0.5	-2	-1.5	0	0
71	3.5	5	5	4.5	4	4	3.5	-2	0	0.5	-0.5	0	-0.5
72	4	4	3.5	4	2.5	3.5	4	-1	-2	-1	-0.5	0.5	-1
73	2	3.5	4	4	3	3	2.5	-2.5	-1.5	0	0.5	0	-1
74	2.5	3.5	4	4	3	2.5	2.5	-0.5	-1.5	0.5	1	-0.5	-1
75	2.5	4	4.5	4	3.5	2.5	2.5	-1	-1.5	-0.5	1	-0.5	-1
76	2.5	3	3.5	3.5	4	3	2.5	0	-1	-0.5	0.5	0	-0.5
77	3.5	3.5	5	5	4	3.5	5	-0.5	-2	0.5	1.5	-1.5	0
78	3	3.5	4	4	3.5	2.5	2.5	-0.5	-1.5	0	-0.5	1.5	-1
79	4	4.5	3.5	4	3.5	3	3.5	-1.5	-2	-1	-1	0	1
80	0.5	3.5	5	2.5	2.5	2.5	3.5	-0.5	2	1.5	2.5	0.5	-0.5
81	4	5	5	5	3.5	2.5	4.5	-2	0	1	2	2.5	-1
82	3	4	4	4	4	3.5	4	0	0	1	1	1.5	0
83	4	5	4	4	5	3.5	3.5	-3	-2	-1	-2	-1.5	-1
84	4.5	2.5	4	4	4	4	2.5	-0.5	-1	-1.5	-1	0	-1
85	4	5	5	5	3.5	4	1.5	-1	-1	1	0	1	-2
86	4	4.5	5	4.5	5	4	4	-0.5	-1	-1	0	-1	-1.5
87	3	2.5	4	4	2.5	2	3.5	0.5	-1	0	-2	1	0
88	5	2.5	2.5	2.5	2.5	2.5	2.5	-0.5	-1	-2	-2	-0.5	1.5
89	2.5	2.5	4	4	4	4	2.5	0.5	-0.5	0.5	-0.5	-2	0
90	4.5	3	4	4	4	4	4	0	-1	-1.5	-1	-3	0
91	4.5	4.5	4	4	3	3	4	-2.5	-3	-1.5	-1.5	-1	0
92	5	5	3.5	5	4	4	4	-3	-1	-1	-2	0	-1
93	4	5	2.5	2.5	2.5	3.5	3.5	-2	-2.5	0	-0.5	-1.5	0.5
94	4	4.5	1.5	2.5	1.5	1.5	2.5	-1.5	-4	0	1.5	1.5	0.5
95	3.5	3	5	5	5	4	3	-1	-1.5	0.5	-0.5	-2	0
96	2	2.5	5	5	5	4	4	0.5	-0.5	2	2.5	0	-1
97	4	2.5	5	3.5	4	4	4	0.5	-2	-1	-2	-1	-0.5
98	5	4.5	4.5	4.5	5	5	5	-1.5	-3	-2	-2	-3	-1.5
99	4.5	4	2	3.5	3	2.5	1.5	-1	-2	-0.5	-1.5	1.5	0.5
100	4.5	4.5	4.5	4	4	4	3.5	-3.5	-3	-1.5	-2	-3	0
101	5	5	5	5	2.5	4.5	5	-2	-2	-1	-3	-2.5	-2
102	5	5	4.5	4	3.5	3.5	4	-2	-1.5	-3	-2.5	0.5	0

ID	LEARN5	CAREER5	PEERRELAT	RESPECT5	SECURE5	PARTICI5	HORNOR5	CAREERfit	INCOMEfit	LEARNfit	BENEFITfi	PARTfit	RESPECTfi
103	3.5	4	5	5	3.5	3.5	4	-2	-3	-0.5	-2	0.5	-1
104	3.5	3	5	4.5	4	4	4.5	0	0.5	0.5	0	0	-0.5
105	2.5	3	3	3	3	3	3	-1	-1	0.5	0	-1	1
106	5	5	4	4	4	4	4	-1	-2	-1	0	-1	0
107	4.5	4	4	3	3	3.5	2.5	0	-1.5	-0.5	-0.5	0.5	0
108	4	3	4	3	2.5	2.5	3.5	0	-1	-1	-1	0.5	0
109	3	5	4	4.5	3	4	4.5	-2	-1	0	-2	-1	-0.5
110	4	4	5	3.5	3.5	4.5	3.5	-2	-4	-1	-4	-0.5	-0.5
111	4	3	4	3	4	3.5	3	-1	-1.5	1	-1	0.5	1
112	2	4.5	5	4.5	3	5	5	-0.5	-1.5	2	2	0	-0.5
113	4	3.5	3	4	4	4	3.5	-1.5	-3	1	-1.5	-1	1
114	4	4.5	3	1.5	2.5	1	2.5	-0.5	-2.5	0	-2	3	2.5
115	5	5	5	5	5	4	5	-3	-3	-2	-2	0	-1
116	4	3	2.5	4	3.5	1.5	1.5	-1	-3	0	-1.5	0.5	-1
117	3.5	4	4.5	4.5	3.5	4.5	3.5	-1	-2	1.5	0.5	-1.5	-0.5
118	2.5	2.5	4	5	4	4	5	-1.5	-1.5	0.5	1.5	-2	-2
119	4	2.5	2.5	4	3.5	1.5	1.5	-0.5	-3	0	-1.5	0.5	-1
120	4	4.5	4	3.5	2.5	1.5	2.5	-2.5	-3	0	-1.5	2.5	0.5
121	3	3	5	4	5	5	5	0	-1.5	0	1	-2	-1
122	3.5	4	3	3	3.5	3	3	-2	-3	-0.5	-1	0	0
123	4	4.5	4	3.5	3	2	3	-2.5	-2	0	-1.5	2	0.5
124	1.5	3	2.5	1.5	2.5	2.5	2.5	-1	1.5	1.5	1.5	2.5	2.5
125	4	4	2.5	3	3	3.5	5	-1	-1.5	0	-0.5	0.5	1
126	3.5	5	3.5	3.5	3.5	1.5	1.5	-2	-0.5	-0.5	0.5	2.5	0.5
127	4	4	5	5	2.5	3	3	-1	-1.5	-1	-2	1	-2
128	4.5	2.5	4	4.5	2.5	4	4.5	1.5	-1.5	-0.5	0	0	-0.5
129	4	2.5	2.5	1.5	1.5	2.5	3	0.5	-0.5	1	-1.5	1.5	2.5
130	3	3	3	2.5	3	2.5	2.5	2	0	1	1	2.5	2.5
131	4	4.5	4.5	4	4	4	4	-1.5	-3	0	-1	0	0
132	4	4	3.5	4	5	3.5	5	-2	-2	0	-3	0.5	0
133	3.5	4	4.5	4.5	5	3.5	5	-2	-2	0.5	-3	0.5	-0.5
134	3.5	4	4.5	4.5	4.5	3.5	5	-2	-1.5	0.5	-3	0.5	-0.5
135	3.5	4.5	4	4	5	2.5	3	-2.5	-2	0.5	0.5	2.5	0
136	4.5	4.5	4.5	4.5	4.5	4	4.5	-2.5	-3	-1.5	-1.5	-1	-0.5

ID	LEARN5	CAREER5	PEERRELAT	RESPECT5	SECURE5	PARTICI5	HORNOR5	CAREERfit	INCOMEfit	LEARNfit	BENEFITfi	PARTfit	RESPECTfi
137	3.5	4.5	3	3.5	3.5	1	1.5	-1.5	-2	-0.5	-0.5	3	0.5
138	5	5	4.5	4.5	5	5	4.5	-3	-2	-3	-3	-3	-1.5
139	3.5	5	3	1.5	1	0.5	0.5	-2	-3	-0.5	-1.5	3.5	2.5
140	3.5	5	3	3.5	4	2.5	2.5	-4	-2	-0.5	-3	1.5	0.5
141	0.5	1.5	4.5	3.5	4	2.5	2.5	0.5	2	1.5	1.5	-1.5	0.5
142	1	1.5	4	4	2.5	2.5	3	1.5	2	2	2	0.5	0
143	5	5	5	5	5	4	5	-1	-3	-3	-1	0	-1
144	3.5	4	5	5	5	3.5	4	-3	-2.5	-0.5	-2	-1.5	-1
145	5	5	5	5	5	5	3	-2	-2	0	-1.5	-1	0
146	5	3.5	5	4.5	5	5	3	-0.5	-2.5	-2	-1.5	-1	-1.5
147	4.5	4.5	3	4.5	3	2.5	1	-3.5	-3	-1.5	-2	-0.5	-0.5
148	4	5	5	5	5	5	4	-1	-2	0	1	0	0
149	2.5	2.5	5	3.5	5	2.5	4	0.5	-1	0.5	0.5	1.5	1.5
150	4.5	4.5	3.5	3.5	3	2	2.5	-0.5	-1	-0.5	-0.5	2	1.5
151	3	3.5	5	4	4.5	4	3.5	-0.5	-2	1	-1	-2	-1
152	3	3.5	5	4	4.5	4	3.5	-0.5	-2	0	0	0	1
153	5	2.5	5	5	2.5	2.5	2.5	0.5	-1	-1	1.5	2.5	-1
154	4	4	5	4	4	4	4	-1	-2	-1	-1	0	0
155	1	2	4	4	4.5	3	4.5	-1	0.5	1	-1.5	-1	0
156	5	4	2.5	5	2.5	2.5	2.5	-1	-1	-2	0.5	1.5	-2
157	3.5	3	4.5	3	2.5	3.5	2.5	-2	0.5	0.5	1.5	0.5	1
158	5	5	5	4	4	4	5	-1	-1	-1	0	0	0
159	4	4	4	2.5	1.5	1.5	1.5	-1	-1.5	0	0	3.5	2.5
160	5	4.5	2.5	2.5	0.5	2	1.5	-0.5	-1.5	0	0.5	2	2.5
161	4	4	4.5	4.5	4.5	4.5	4	0	0	0	0	-0.5	-0.5
162	4	4	4	4	3.5	3.5	3.5	-1	-1.5	0	-1.5	-1.5	0
163	1	2.5	1	2.5	1	0.5	0.5	1.5	1.5	3	3.5	3.5	1.5
164	1.5	2	4.5	4	4.5	4	4	0	0	0.5	-1	-2	0
165	3	2.5	2.5	2.5	2.5	1.5	1.5	-1.5	0	-1	1.5	0.5	0.5
166	2.5	3	2.5	2.5	1.5	2.5	2.5	0	-1	0.5	2	0.5	1.5
167	3	4	5	4.5	4	4	3	-2	-2.5	0	0.5	-3	-0.5
168	4	3.5	4	5	3.5	3.5	2.5	-0.5	-0.5	-1	-0.5	0.5	-1
169	3.5	4	2.5	4	3	3	3	-1	-1	-0.5	0	1	-1
170	3	4	4	3.5	3.5	3	2.5	-1	-1.5	0	0.5	1	-0.5

ID	LEARN5	CAREER5	PEERRELAT	RESPECT5	SECURE5	PARTICI5	HORNOR5	CAREERfit	INCOMEfit	LEARNfit	BENEFITfi	PARTfit	RESPECTfi
171	4	3.5	4.5	4	4	2	3.5	-0.5	-2	0	-1	2	0
172	2.5	1.5	4.5	4	3.5	3.5	4	1.5	0	0.5	-2	-2.5	0
173	4.5	4	3.5	3.5	1.5	2.5	1.5	0	2.5	-0.5	0.5	2.5	0.5
174	4.5	5	2.5	3.5	3	2.5	3	-2	-1.5	-0.5	-2	0.5	0.5
175	5	5	3.5	3.5	1.5	3.5	4	-2	0	0	0	0.5	0.5
176	4.5	4	3	2.5	3	2.5	1.5	-3	-1.5	-1.5	-3.5	-1.5	-1.5
177	5	4	2	3	1	3.5	2.5	-2	-3.5	-3	-1.5	-1.5	0
178	2.5	2.5	4	4.5	4	2	2	-0.5	0	0.5	1.5	0	-0.5
179	2.5	2.5	4.5	4.5	4	4.5	3	-0.5	0.5	0.5	0.5	-2.5	-0.5
180	3.5	4.5	4	3.5	2.5	2.5	3	-0.5	-1	-0.5	-2	0.5	0.5
181	5	4	4.5	5	4.5	4	4.5	-1	-1	0	-0.5	0	-1
182	4	3.5	3.5	3.5	4	3.5	3.5	-1.5	-1	0	-1.5	0.5	0.5
183	3.5	2.5	5	5	5	5	4	1.5	1.5	0.5	0	-4	-1
184	3.5	4	5	5	4	3.5	3.5	-1	-0.5	-0.5	-0.5	-1.5	-1
185	3.5	4	3.5	5	4.5	4	3.5	-1	-2	-0.5	-2	-2	-2
186	4.5	4.5	4	3.5	4.5	4.5	4.5	-1.5	-1.5	-1.5	-1.5	-0.5	-0.5
187	0.5	1	4	5	4	1.5	2.5	0	2	1.5	0.5	0.5	0
188	4	4.5	4	4	3	3.5	4	-1.5	-1.5	-1	-1	-0.5	0
189	2.5	2	4	1	2	3	3.5	1	1	0.5	0	0	2
190	2	3.5	5	4	5	2.5	3.5	-2.5	-1	0	1	1.5	0
191	4	3	5	5	5	4.5	5	-2	-2.5	0	-2.5	-2.5	-2
192	2	2	5	3.5	4	3.5	5	0	-0.5	1	0.5	-0.5	0.5
193	2.5	4	2.5	4	4	4	4	0	-1.5	1.5	-1	0	0
194	5	4.5	4.5	4.5	2.5	2.5	3.5	-2.5	-3	-4	-1	-0.5	-0.5
195	3	4.5	4.5	2.5	2	2.5	2.5	-3.5	-4	-1	-4	-1.5	1.5
196	4	2.5	4	3.5	4.5	2	1	-0.5	-1	-1	-1.5	0	0.5
197	3.5	3	5	4	5	5	5	-2	-2	0.5	1.5	-2	1
198	3.5	5	2.5	3.5	2	3.5	2.5	-4	-3	-1.5	-1.5	-1.5	-0.5
199	1	0.5	4	5	5	4	5	2.5	1	2	0	-3	-1
200	5	5	4	4.5	3.5	2	2.5	-1	-4	-3	-3	0	0.5
201	5	4.5	3.5	4	3.5	3.5	4	-1.5	-2.5	-2	-3	-0.5	0
202	3.5	3	4.5	4	4.5	4.5	4	1	0	0.5	0	0.5	0
203	3.5	4	4.5	4.5	4.5	2.5	2.5	-1	-1	-0.5	1	0.5	-1.5
204	1	1	4.5	1	1.5	1	4	2	1	2	3	2	2

ID	LEARN5	CAREER5	PEERRELAT	RESPECT5	SECURE5	PARTICI5	HORNOR5	CAREERfit	INCOMEfit	LEARNfit	BENEFITfi	PARTfit	RESPECTfi
205	2.5	4	2.5	2.5	2.5	2.5	1	1	0.5	1.5	2	1.5	1.5
206	1	1.5	5	3.5	4.5	2.5	4	1.5	0.5	3	1.5	1.5	0.5
207	4.5	4.5	3.5	2.5	1.5	1.5	2.5	-1.5	-1.5	-0.5	-1	3.5	1.5
208	4	4.5	3.5	3	2.5	0.5	1.5	-3.5	-2	-3	-3.5	2.5	0
209	3	3.5	3	2.5	2	2	3.5	-0.5	-0.5	0	-1	2	0.5
210	5	5	5	4	5	3.5	5	-4	-3.5	-1	-2	0.5	1
211	4.5	5	4	4	3	3	3	-2	-2	0.5	-1	1	1
212	0.5	1	2.5	4	2.5	1	0.5	1	0.5	1.5	1.5	2	0
213	5	5	5	5	5	2.5	5	-1	0.5	-2	0	1.5	0
214	4	4.5	5	5	4	2.5	5	-1.5	2	-1	1.5	1.5	-1
215	5	4	2.5	4	4	4	4	-1	-1	-1	0	0	0
216	3.5	3	2	3.5	1.5	2	1.5	0	-0.5	-0.5	3	2	0.5
217	2.5	1	4.5	4.5	4	4	4	2	-0.5	0.5	1.5	-1	-0.5
218	4	4.5	2	1.5	0.5	0.5	3.5	-1.5	-1	-1	0	3.5	1.5
219	4	5	5	5	4	3.5	2.5	-4	-2	-2	-3	-2.5	0
220	3.5	5	4.5	3	4	3	4	-2	-3	-0.5	1.5	1	1
221	4.5	3.5	4	5	3.5	4	4	-0.5	-2	-1.5	-1.5	-1	-2
222	5	5	5	2.5	4	2.5	2.5	-2	-3	-3	-3	0.5	1.5
223	4	2.5	5	4	2.5	1.5	4	-1.5	-1	-2	-3.5	-0.5	0
224	4	2.5	5	4	3.5	1.5	4	-1.5	-1	-1	-3	-0.5	0
225	4	5	4.5	4.5	3.5	4	4	-2	-4	0	-2	0	-1.5
226	5	5	5	5	5	5	5	-3	-4	-3	-3	-2	-2
227	1.5	0.5	4	3	2.5	2.5	2	0.5	3.5	1.5	1.5	0.5	0
228	3.5	4.5	2.5	2.5	0.5	1	0.5	-3.5	-3	-0.5	-3	1	1.5
229	4.5	4.5	4	4	3.5	3	3	-2.5	-2.5	-0.5	-1.5	-1	0
230	4.5	4.5	4.5	4	3.5	4.5	4	-1.5	-2	-1.5	-1	-0.5	-1
231	4.5	5	4	3.5	3.5	2.5	3	-2	-3	-3.5	-1	1.5	0.5
232	4.5	5	4	1.5	4	2.5	0.5	-2	-2	-0.5	-1.5	1.5	2.5
233	2.5	5	2.5	2.5	1.5	2	0.5	-3	-2.5	-0.5	-0.5	2	1.5
234	3	2.5	1	3	1.5	3	2	0.5	-2.5	0	-3	-1	1
235	5	2.5	4.5	4	5	4	5	-1.5	-4	0	-2	-2	0
236	3	4	5	4.5	4	3	3	0	-0.5	2	4	2	-0.5
237	4.5	4.5	4	3.5	5	2	5	-3.5	-4	-1.5	-3	0	0.5
238	4.5	4.5	4.5	4.5	4.5	4.5	4.5	-0.5	-1.5	-0.5	-0.5	0.5	-0.5

ID	LEARN5	CAREER5	PEERRELAT	RESPECT5	SECURE5	PARTICI5	HORNOR5	CAREERfit	INCOMEfit	LEARNfit	BENEFITfi	PARTfit	RESPECTfi
239	2.5	3	4	4	2.5	2	2.5	0	-1	0.5	-1.5	2	0
240	4	3	4	4.5	5	5	4	-2	-2	-1	-1.5	-4	-1.5
241	1.5	2.5	3.5	3.5	3.5	1.5	2	-1.5	2.5	0.5	1.5	-0.5	0.5
242	5	2.5	2.5	3.5	2	2.5	2	-1.5	-0.5	-3	0.5	-0.5	0.5
243	4	4.5	3	2.5	2.5	2	2.5	-3.5	-4	-1	-3	0	1.5
244	4	5	5	4	4	4	2.5	-4	-2	-1	-3	-2	-1
245	4.5	5	5	5	5	4	5	-2	-2	-1.5	-2	-2	-1
246	4.5	5	4	4	4	4	4.5	-3	-1	-1.5	-2	-1	-1
247	4	4.5	4	5	4	3	2.5	-2.5	-2	-1	-2	0	-1
248	4	4.5	4	3.5	3.5	3	4.5	-2.5	-3	-1	-3	1	-0.5
249	3	3	3.5	3.5	3	3	3	0	0	1	0	1	0.5
250	4	4	2.5	3	4	4	4	-1	-1	-1	-1	0	0
251	3	3	4.5	5	4.5	2.5	5	-2	-1	1	-1	-0.5	0
252	4	2.5	4	4	4	4	4	-1.5	-1	0	-1	-2	1
253	2.5	2.5	4.5	4.5	3.5	4	4	0.5	1.5	1.5	-2.5	0	-0.5
254	3.5	3	4.5	4	4.5	4	5	0	0	-0.5	-0.5	0	0
255	4	4.5	3	4	3	2.5	2.5	-1.5	-1.5	-1	-1	-0.5	0
256	5	4.5	3	2.5	1	1.5	3.5	-0.5	-2	-1	-2	2.5	0.5
257	5	5	3.5	4	2	3.5	3	-2	-1	-2	-1	-0.5	0
258	3.5	2.5	0.5	0.5	1	0.5	0.5	1.5	-2	0.5	0	3.5	2.5
259	4.5	4	3.5	4	1.5	3	2.5	0	-1.5	-0.5	1	1	1
260	4	5	4	1.5	1	2.5	3	-1	-1.5	0	0	1.5	0.5
261	5	4.5	4.5	4.5	5	5	5	-1.5	-3	-2	-2	-1	-1.5
262	3	4	3	4	4	4	4	-1	-1	0	-1	-1	-2
263	3.5	4.5	2.5	2.5	1.5	2.5	2	-1.5	-3	0.5	-0.5	1.5	1.5
264	3.5	4	4	4.5	1.5	1.5	0.5	0	-3	0.5	0	2.5	0.5
265	2.5	4	1.5	2.5	3.5	3.5	2.5	-1	-2	0.5	-1.5	-1.5	-0.5
266	4.5	4	4	3	4	4	4	0	-1.5	-0.5	-1	0	1
267	4	5	5	5	4	4	5	-2	-4	-1	-2	-2	-1
268	4	5	3	4	4	2	1.5	-2	-2	-1	-0.5	1	-1
269	4	5	5	4.5	5	5	4	-3	-4	0	-2	-3	-0.5
270	2	2.5	5	4	5	2.5	4	-0.5	-2.5	2	-1	-0.5	0
271	4	3.5	3	4	4.5	4	5	-0.5	-2	-1	-1	-2	0
272	2.5	3	4	3	3.5	3.5	2.5	0	-0.5	0.5	0.5	-0.5	1

ID	LEARN5	CAREER5	PEERRELAT	RESPECT5	SECURE5	PARTICI5	HORNOR5	CAREERfit	INCOMEfit	LEARNfit	BENEFITfi	PARTfit	RESPECTfi
273	5	3	5	4	5	4	2.5	0	-1	-1	-1	0	0
274	4	5	3	3.5	3	2	3.5	-1	-1.5	0	-1	3	0.5
275	5	4.5	4.5	4.5	5	4.5	4.5	-1.5	-2.5	-2	-2	-0.5	-0.5
276	4	2.5	4	4	4	4	4	0.5	0.5	0	0	0	0
277	3.5	2.5	2.5	2.5	2.5	5	3	0.5	-3	0.5	-2	-3	0.5
278	4	2.5	2.5	2.5	2.5	5	3.5	0.5	-3	-1	-2	-3	1.5
279	4.5	5	4	4.5	4.5	4.5	4.5	-3	-2.5	-1.5	-1.5	-1.5	-0.5
280	4	4	4	3	4	4	4	0	-1.5	0	0	0	0
281	3.5	4.5	3.5	2.5	2	0.5	0.5	-0.5	-3	0.5	0.5	3.5	2.5
282	3.5	4	4	4.5	4	3	3.5	-3	-2	-0.5	-3	-1	-0.5
283	4	4	4	4	4	4	5	-1	-3	-1	0	0	-1
284	3	4	4.5	2.5	4.5	0.5	2	-3	-1	0	-2	1.5	0.5
285	3	4	4.5	4	4	3.5	3.5	-2	0	0	0	0.5	0
286	3	4	4	4.5	5	3	3.5	1	-1	2	0	2	0.5
287	2.5	2.5	5	3.5	4.5	4	3	0.5	0	0.5	0	0	-0.5
288	2	4	5	4.5	5	1.5	3.5	1	-1	3	0	3.5	0.5
289	3.5	4	4	3	3.5	3.5	2.5	-1	1.5	-0.5	0.5	-1.5	-1
290	4	4	4	4	3.5	4	4	-1	0.5	0	0	1	-1
291	4	3.5	4	3	3.5	4	4	-1.5	-2	-2	-0.5	-1	-1
292	3	3	5	3.5	4.5	4	3.5	0	-1	0	-1	0	0.5
293	2.5	3	4	4	5	3.5	4.5	-1	-0.5	1.5	2	1.5	0
294	2.5	3.5	4	3.5	4	3.5	3	-0.5	-1	0.5	-0.5	0.5	0.5
295	3	3	4.5	5	4.5	2.5	5	-2	-1	0	0	0.5	-2
296	2	2.5	4	5	5	5	5	1.5	-0.5	2	1.5	0	-2
297	3	2	5	4	4.5	4	5	1	-2	0	1	-1	0
298	3	4	3	4	4	4	4	-1	-1	0	-1	-1	-2
299	4	4.5	3.5	3	4	4	4	-1.5	-3	-1	-2	0	1
300	3.5	2.5	2.5	2.5	2.5	1.5	2.5	-0.5	-0.5	-0.5	-0.5	0.5	0.5
301	5	5	3.5	4	3	1.5	4	-1	-3	-1	1	3.5	0
302	4.5	4	4	4	4.5	4.5	4.5	-1	-2.5	-0.5	-1.5	-0.5	-1
303	4.5	4.5	4.5	4.5	4.5	4.5	4.5	-0.5	-2.5	-0.5	-0.5	-0.5	-0.5
304	3	5	3	4.5	4	2	3	-2	-3	0	-2	2	-1.5
305	5	4	4	4	3.5	3.5	3	0	-3	-1	-1	1.5	0
306	4.5	4.5	4.5	4.5	4.5	4.5	4.5	-1.5	-2.5	-1.5	-1.5	-1.5	-0.5

ID	LEARN5	CAREER5	PEERRELAT	RESPECT5	SECURE5	PARTICI5	HORNOR5	CAREERfit	INCOMEfit	LEARNfit	BENEFITfi	PARTfit	RESPECTfi
307	4	4	4	4	4	3	4	1	-3	0	0	1	1
308	2.5	2.5	4.5	4	3.5	3	3	-0.5	-0.5	0.5	1.5	1	0
309	4.5	4.5	4.5	4.5	4.5	4.5	4.5	-1.5	-1.5	-1.5	1	-2.5	-1.5
310	4.5	5	5	4	5	5	5	-2	-2	0.5	-0.5	-1	-1
311	4.5	5	3.5	4.5	1.5	2.5	3	-1	-3	-0.5	-1	1.5	-1.5
312	4	4.5	3	3	3	3	3.5	-1.5	-3	-1	-0.5	2	1
313	4.5	5	3	3.5	3.5	3	2.5	-2	-1.5	-1.5	-1	1	0.5
314	5	2.5	4	5	5	4	5	0.5	-2	-1	-0.5	-1	-1
315	4	4	5	4	4.5	4	4	-1	-1	-1	-1	0	0
316	4	3	5	4	5	3	3	-1	-3	-1	-2	1	-1
317	3.5	2.5	3	3.5	4	3.5	3.5	0.5	-1	-0.5	-1	0.5	0.5
318	3.5	4	5	4	4	4	4	-1	-2	-0.5	-1	-2	0
319	3.5	4	5	4	4	4	4	-1	-2	-0.5	0	-2	0
320	5	5	5	5	5	4.5	5	-2	-2	-1	-1	-0.5	-2
321	4	5	3.5	2.5	2	2.5	3.5	-1	-1	-1	0.5	1.5	1.5
322	4.5	5	5	4	4	4	3.5	-2	-2	-1.5	-2	-2	-1
323	4	4	3	3	2.5	2.5	4	-1	-2	0	0	1.5	1
324	4	4.5	3.5	4	2.5	1.5	2	-3.5	-1	-2	-0.5	1.5	-2
325	3.5	4	3	2.5	3.5	2	2.5	-1	-1	-0.5	-1	1	0.5
326	4	4	5	4	4	4	4	-1	-1	0	0	0	0
327	4	4	4	4	5	4	5	0	-2	0	-1	1	0
328	3.5	3.5	4	4	4	3.5	3.5	-0.5	-1.5	0.5	-2	-0.5	0
329	3.5	3	3.5	4.5	4	3.5	3.5	0	0.5	-0.5	1	0.5	-0.5
330	4	4	4.5	4.5	5	4	3	-1	-3	-1	-1	-1	-0.5
331	4.5	4.5	4	4	3	3.5	3	-1.5	-3	-1.5	-2.5	0.5	-1
332	1.5	2.5	5	1.5	3.5	4	3.5	0.5	2	1.5	2	1	1.5
333	4.5	3	2.5	2.5	3	1	1.5	0	-1.5	-1.5	-1.5	4	0.5
334	4	4	4	4	4	4	4	0	0	0	0	0	0
335	3	3.5	5	4.5	5	5	4	-0.5	-0.5	2	0.5	0	0.5
336	5	5	5	5	4	4	4	-2	-2	-2	-2	-1	-1
337	3	3	4.5	4.5	4.5	3.5	3	0	1.5	1	1.5	0.5	-0.5
338	4.5	4.5	3	3.5	2.5	2.5	3	-0.5	-1	-0.5	0	1.5	1.5
339	5	4	5	5	5	4	4	-1	-2	-2	-2	0	-1
340	4	5	4	4.5	4	3.5	3	-2	-1.5	0	-1	-0.5	-2.5

ID	LEARN5	CAREER5	PEERRELAT	RESPECT5	SECURE5	PARTICI5	HORNOR5	CAREERfit	INCOMEfit	LEARNfit	BENEFITfi	PARTfit	RESPECTfi
341	5	5	5	5	4	4.5	4.5	-3	-3	-4	-2	-1.5	-2
342	4	4	4	4	4	2.5	4	-1	-1	0	0	0.5	0
343	5	5	4.5	4.5	4.5	4.5	4.5	-3	-3	-3	-1	-0.5	-0.5
344	2.5	2.5	2.5	4	5	2.5	2.5	0.5	0.5	1.5	1.5	1.5	0
345	4.5	4.5	4	4	4.5	4.5	4	-1.5	-0.5	-2.5	-2.5	-1.5	0
346	4	4	4	3	4	4	3	-2	0	-1	-1	-1	1
347	4.5	5	3.5	4.5	5	5	1.5	-2	-2	-0.5	-2	-2	-1.5
348	4.5	4	5	5	5	5	5	0	-2	-0.5	0	-1	0
349	4.5	4	5	5	5	5	4.5	0	-2	-0.5	-1	-1	0
350	3	2.5	3.5	4	2.5	1	1	0.5	-0.5	0	1.5	1	-1
351	1	2.5	2.5	2	2.5	2	0.5	-0.5	2	2	2	1	1
352	1	1	4	4.5	5	4	5	3	0.5	3	0	-1	-0.5
353	2	2.5	4.5	4	4.5	3.5	4.5	-0.5	-0.5	2	-1	-1.5	0
354	4.5	5	2.5	2.5	2	1	2.5	-3	-2	-0.5	0	4	0.5
355	4	5	1.5	1	0.5	2.5	2	-1	-1	1	-0.5	1.5	4
356	4	5	4	4	4.5	5	5	-3	-1	-2	-2	-3	-1
357	4.5	3.5	4	4	4	3.5	2.5	-0.5	1.5	-0.5	1	-0.5	0
358	4	5	2.5	3	3.5	2.5	1.5	-2	-1.5	-1	-1	-0.5	1
359	5	5	5	4	5	5	2.5	-3	-3	-2	-3	-2	1
360	4.5	5	3.5	4	4.5	3.5	3	-3	-2.5	-1.5	-2	-1.5	0
361	5	5	5	5	3.5	4	5	-3	-4	-2	-3	-2	-1
362	4	4	2.5	2.5	1.5	3	2	-2	0	-1	-3	-1	1.5
363	4	4.5	3.5	2.5	2	3.5	3.5	-3.5	-3	-1	-2	-0.5	0.5
364	4	4.5	4.5	4	4.5	4	4	-0.5	-2	0	-2	1	0
365	4	4	2.5	4	3.5	3	3.5	-1	-3	-2	-3	1	0
366	4	5	2	4	3	2.5	2.5	-1	-4	1	-0.5	1.5	0
367	4.5	5	4	4	2	2.5	3.5	-1	-4	-0.5	0	1.5	1
368	5	4	2.5	3.5	3	3.5	3	0	-2	-2	-2	-0.5	0.5
369	5	2.5	2	4	4	2	1.5	0.5	0.5	-3	-2	1	0
370	5	2.5	5	2.5	2.5	2.5	4	1.5	2.5	-1	1.5	2.5	1.5
371	4	5	4	4	5	3.5	4	-2	-1	0	-1	0.5	1
372	4	4	4.5	5	4.5	3	3.5	0	-0.5	0	0	2	-1
373	2.5	3	4	3.5	2.5	3	1.5	0	1	1.5	1.5	1	0.5
374	4	5	5	5	3.5	4.5	4	-2	-4	1	-1	-3.5	0

ID	LEARN5	CAREER5	PEERRELAT	RESPECT5	SECURE5	PARTICI5	HORNOR5	CAREERfit	INCOMEfit	LEARNfit	BENEFITfi	PARTfit	RESPECTfi
375	4	4	4	5	4	4	4	0	-1	0	0	1	-1
376	3.5	4	4.5	5	4.5	3	3.5	0	-0.5	0.5	0	2	-1
377	2.5	1.5	4	4	3.5	3	3	2.5	-1.5	0.5	0	0	1
378	4.5	5	3.5	4	3.5	3	3	-1	-4	-0.5	-1	1	0
379	4	5	4	4	3.5	2.5	4	-3	-2	-1	-2	-1.5	0
380	5	2.5	5	5	5	2.5	2.5	0.5	-2	-2	-1	1.5	0
381	2	5	4	4	2.5	3.5	2.5	-3	-2	2	0	1.5	0
382	3.5	5	3	2.5	2.5	1.5	2.5	-2	-2	-0.5	-3	2.5	1.5
383	3.5	4.5	2.5	4	3	4	3	-1.5	-4	-0.5	-1.5	0	-1
384	5	4.5	2.5	3.5	1.5	3	3	-1.5	-2.5	0	-1	0	0.5
385	4	4.5	5	5	5	2.5	3.5	-1.5	0.5	-1	1	-0.5	0
386	3.5	3	5	4.5	4.5	4.5	4	0	-3	0.5	-1	-0.5	0.5
387	3.5	4.5	4	4	3	0.5	2.5	-2.5	-3	-1.5	-1	0.5	0
388	4	4.5	5	5	5	4.5	3	-1.5	0.5	1	0	-2.5	0
389	2.5	3	2	3	1.5	2.5	2.5	0	-0.5	0.5	0.5	1.5	1
390	4.5	5	5	4.5	4.5	4.5	4.5	-1	-1.5	0.5	-0.5	-1.5	-0.5
391	5	4.5	4	4.5	4.5	4	4	-1.5	-1	-2	-1.5	0	-0.5
392	5	4	3	3.5	3	1	2.5	-1	-0.5	-3	0	3	0.5
393	3	3.5	4.5	4	5	3.5	5	0.5	-0.5	0	-0.5	-0.5	0
394	1.5	1	3	5	2.5	2.5	2.5	2	3.5	1.5	0	0.5	-1
395	2.5	3	4	2.5	2	2	3	1	0.5	1.5	1	2	1.5
396	3	3	4	4	4	4	4	0	-1	0	-1	-1	-1
397	4.5	4	5	4	5	2	4.5	-1	0.5	-1.5	-1	1	-1
398	4	5	5	5	2.5	3	3.5	-2	-1.5	-1	-1.5	1	-2
399	3	3	3.5	3.5	3	3.5	3.5	0	-2	1	-2	-1.5	-0.5
400	3.5	4	5	4.5	5	5	3	-2	-0.5	-0.5	-0.5	-1	-0.5
401	4	4	5	5	3.5	4	5	-2	-2	-1	-1	-2	0
402	3.5	4	1	1.5	1	1.5	2.5	0	-2	0.5	-0.5	1.5	2.5
403	2.5	1	4	3	4.5	4	1.5	1	1.5	0.5	0.5	0	1
404	4	4.5	4	4	5	4	4	-1.5	-3	-1	-2	0	-1
405	3	3.5	5	2.5	3.5	3.5	3.5	-0.5	1	0	0.5	-0.5	0.5
406	5	4.5	5	4.5	5	4.5	4.5	-0.5	-1	-3	-2	-1.5	-1.5
407	3.5	4	1.5	2.5	1.5	0.5	1.5	-2	-2	0.5	-2.5	2.5	-1.5
408	4	4.5	4.5	5	5	1	5	-1.5	1	-1	1	3	-2

ID	LEARN5	CAREER5	PEERRELAT	RESPECT5	SECURE5	PARTICI5	HORNOR5	CAREERfit	INCOMEfit	LEARNfit	BENEFITfi	PARTfit	RESPECTfi
409	4.5	4.5	4.5	4.5	5	4.5	4.5	-1.5	-1	-1.5	-1.5	-1.5	-0.5
410	5	4	5	5	5	4	5	-2	-2	-2	-1	-2	0
411	4.5	4.5	4.5	4.5	4.5	4.5	4	-1.5	-2.5	-0.5	-0.5	-0.5	-0.5
412	5	5	5	5	5	5	5	-2	-3	-2	-2	-1	-1
413	4	4	4	4	4	4	4	-1	0.5	0	0.5	0	1
414	5	3	5	4.5	5	5	5	0	-2	-1	-2	-2	-0.5
415	4	4.5	4.5	4.5	4	4	4	-1.5	-3	0	-2	0	-1.5
416	5	5	5	4.5	5	5	5	-1	0.5	-1	-1	-1	-0.5
417	4	4	5	5	2	3	3	0	-4	-1	-1	2	-1
418	4.5	4.5	5	4.5	4.5	4.5	4.5	-0.5	-2.5	-0.5	-0.5	-0.5	-0.5
419	4	5	4	4.5	3.5	5	2.5	0	-1.5	1	0.5	0	0.5
420	2.5	2.5	2.5	2.5	2.5	2.5	2.5	0.5	0.5	1.5	0.5	1.5	1.5
421	5	4.5	4.5	4.5	4	4	4	-2.5	-2	-3	-1.5	0	-0.5
422	4	4	5	5	4.5	4	4	-2	-1.5	0	-1	0	-1
423	4	3.5	5	4.5	4.5	4	4	-0.5	-1	-2	0	0	0.5
424	4	4	4	4	4	4	4	-1	-1	-1	0	-1	-1
425	4	3.5	3.5	3.5	4	4	5	-2.5	-3	-1	-3	-2	0.5
426	2.5	3	5	4	4.5	2.5	3.5	0	0	0.5	1.5	1.5	0
427	4.5	4.5	5	4	4.5	5	5	-0.5	-1	-0.5	-1	0	0
428	5	4	2.5	4	4	4	4	0	-1	-1	0	0	0
429	5	5	4	3	2.5	2.5	3.5	0	-2	-1	-1	1.5	1
430	3.5	2.5	5	5	5	3.5	5	0.5	-1	-0.5	-1	0.5	-1
431	2.5	2.5	5	5	4	4	4	1.5	-0.5	1.5	1	0	-1
432	3.5	4	4.5	4.5	4.5	3.5	3.5	-1	-1.5	-0.5	-1	-1.5	-0.5
433	1.5	0.5	4	5	1.5	2.5	3.5	0.5	0.5	2.5	1.5	1.5	-1
434	3.5	3.5	4	4	4	3.5	3	-0.5	0	0.5	0.5	0.5	0
435	4.5	5	3	4	3	3.5	1.5	-2	-3	0.5	-1	0.5	1
436	3.5	3.5	2.5	1	2	1	2.5	0.5	-1	0.5	-0.5	1	2
437	4	4.5	4.5	4	4	4	4	-1.5	-1.5	0	-0.5	-1	0
438	4	4	4	4	4	4	4	1	-2	0	-1	0	0
439	3.5	4.5	4.5	4.5	5	4	4	-1.5	-3	0.5	-1	0	0.5
440	4	5	4.5	3.5	3.5	2.5	4	-1	-1	0	1.5	1.5	0.5
441	2	3	4	4	3.5	3	4	-1	0.5	1	-0.5	-1	0
442	4.5	5	4.5	4.5	4	4	4.5	-2	-3.5	-0.5	-1.5	0	-0.5

ID	LEARN5	CAREER5	PEERRELAT	RESPECT5	SECURE5	PARTICI5	HORNOR5	CAREERfit	INCOMEfit	LEARNfit	BENEFITfi	PARTfit	RESPECTfi
443	4	4	4	5	5	4	4	-1	-3	0	-2	-2	-1
444	5	5	1.5	1.5	0.5	0.5	0.5	-2	0	-2	0	3.5	1.5
445	2.5	4	4	4	4	3.5	2.5	-1	0.5	0.5	0.5	-1.5	0
446	4	4	5	5	5	3.5	3.5	-1	0	-1	-0.5	0.5	-1
447	4	4.5	3.5	3	3	2	2.5	-1.5	-1	-1	-1.5	2	1
448	5	5	5	5	5	4	4	-2	-2	-2	-2	0	-2
449	4	2.5	5	2.5	3.5	2.5	2.5	-0.5	-2	-1	-2.5	-0.5	1.5
450	4	4	4	3.5	4.5	4	4.5	-1	-1	-1	-1.5	0	-0.5
451	3	2	3.5	2	4	2.5	4	-1	-0.5	0	1	1.5	2
452	2.5	3.5	4	4	4	3.5	2.5	-2.5	-0.5	-1.5	-1.5	-1.5	-1
453	2.5	2.5	5	4.5	5	4	4.5	0.5	0	0.5	0.5	0	-0.5
454	4	5	5	4	5	4	4	-2	-2	-1	-2	0	0
455	4	4	5	5	5	3	3.5	-1	-1	0	0.5	1	-2
456	1.5	1	4	1	1.5	2.5	3	2	0.5	2.5	2	0.5	2
457	3	4	3	3.5	4	3.5	3	0	-1	0	0	0.5	1.5
458	4	1.5	4	5	5	3.5	2.5	1.5	-3	-1	0.5	0.5	-1
459	4	4.5	4.5	5	4	4	5	-2.5	-2	0	-2	0	-1
460	1	2.5	2.5	2.5	3	2.5	0.5	0.5	1	3	1.5	0.5	0.5
461	2.5	2.5	3	3	2.5	2.5	2	0.5	-1	0.5	0.5	2.5	0
462	5	5	5	5	4	4	4	-3	-2	-1	-3	-2	0
463	2.5	3	4	3.5	4	3	3.5	0	0.5	1.5	0.5	-2	1.5
464	3.5	4.5	4.5	4.5	3.5	4	2.5	-1.5	-0.5	0.5	0.5	-1	0.5
465	4.5	4	4	5	4.5	4.5	5	-1	-2	-1.5	-1.5	-0.5	0
466	4.5	5	4	4.5	3	3	3	-2	-4	-1.5	-1	0	-0.5
467	3	4	4	3	4	3.5	3.5	0	-0.5	1	-0.5	0.5	1
468	4	5	4	2.5	3.5	3	3	-1	-2	0	-2	1	1.5
469	3	3	3	3	4	3	3	1	0	0	0	1	1
470	3.5	1.5	4.5	4.5	4	5	2.5	1.5	-1	-0.5	-0.5	-1	-0.5
471	1.5	2.5	4	4.5	4	3.5	4.5	0.5	-1.5	1.5	0.5	-2.5	-0.5
472	4.5	4	2	2.5	4	2.5	3	-2	-0.5	-1.5	-1	1.5	1.5
473	5	5	5	4	5	2.5	2.5	-1	-0.5	-1	-2	0.5	1
474	4.5	4.5	4.5	4.5	4.5	4	4	-1.5	-1.5	-2.5	0.5	0	-0.5
475	3.5	4.5	4	3.5	4.5	4.5	4	-3.5	-1.5	-0.5	-2	-1.5	1.5
476	5	4	5	5	4	4	5	-2	-1	-4	-2	-2	-1

ID	LEARN5	CAREER5	PEERRELAT	RESPECT5	SECURE5	PARTICI5	HORNOR5	CAREERfit	INCOMEfit	LEARNfit	BENEFITfi	PARTfit	RESPECTfi
477	1.5	2.5	4.5	3.5	1.5	2.5	3.5	0.5	0.5	1.5	-0.5	0.5	0.5
478	3.5	4.5	4	4	5	3.5	3	-1.5	-2	0.5	-1	0.5	0
479	4.5	4	4	4.5	4	4	4.5	-1	-1.5	-1.5	-1.5	-2	-0.5
480	3.5	4.5	1	2	1	1.5	0.5	-0.5	-1.5	1.5	-3	3.5	3
481	4	4	3	3	3	1	3.5	-1	-3	-1	0	1	1
482	3	4.5	2.5	3	0.5	0.5	1	-1.5	-2.5	1	-1	3.5	1
483	3.5	4.5	3.5	3	3	2.5	2	-3.5	-4	-0.5	-3	0.5	1
484	2.5	2.5	5	4.5	3	2.5	2.5	0.5	1.5	0.5	1.5	0.5	-0.5
485	2.5	3	4.5	4	4.5	4.5	4	-1	-2	0.5	-2.5	-0.5	0
486	5	5	4.5	4.5	5	5	4.5	-3	-3	-2	-2	-1	-1.5
487	4	4	5	2.5	4.5	4	4	-3	-1.5	-2	-1	-2	1.5
488	1	0.5	3.5	4	2.5	1	1	0.5	0.5	3	1.5	2	0
489	3.5	5	3	2.5	4	2.5	5	-2	-3	-0.5	-3	0.5	1.5
490	5	5	3	4	3	1.5	2.5	-2	-3	-2	-2	0.5	0
491	4.5	2.5	5	4	4.5	5	4.5	0.5	-3	-1.5	-3	-4	0
492	5	4	5	3	2	5	2.5	-1	-4	-1	-2	-1	1
493	1.5	1.5	4	4	5	3.5	3.5	-0.5	-0.5	0.5	0	0.5	0
494	3.5	2.5	3	1.5	1	5	3	0.5	0	0.5	2	-1	1.5
495	4	5	4	4	5	5	4	-4	-3	-2	-4	-1	-3
496	1.5	2.5	5	4	2	2.5	4	1.5	1	1.5	0	1.5	0
497	1.5	2.5	3	3	1.5	1.5	1.5	0.5	1.5	1.5	1.5	0.5	0
498	5	5	5	5	5	5	5	-1	-1	-1	0.5	-1	-1
499	4	4	4	4	4	4	4	0	-1.5	-1	-1	0	1
500	4	4	4	4	4	3.5	3	0	-2	0	0	-1.5	0
501	3	4	2.5	3.5	2.5	4.5	3.5	-3	-4	0	-3	-1.5	0.5
502	4.5	5	4	4	4	5	1.5	-1	-2	-1.5	-2	-1	0
503	4	1	4	5	5	2	3.5	1	-2	-1	-3.5	1	0
504	3.5	3.5	2.5	2	3.5	2.5	2	-1.5	-2	-0.5	-1.5	0.5	1
505	5	5	4	4	3	4	3.5	-3	-3.5	-1	-2	0	0
506	3.5	3.5	2.5	2.5	1.5	0.5	0.5	-2.5	-4	-0.5	-3	1.5	1.5
507	4.5	4.5	4	3	3.5	2.5	3	-1.5	-2	-1.5	-2	1.5	0
508	2	1	1	1.5	3.5	4.5	2.5	2	0.5	1	0.5	-3.5	1.5
509	3	3.5	3.5	3.5	3.5	3	3.5	-1.5	-3	0	-3	-1	0.5
510	5	5	5	5	5	4.5	3.5	-3	-3	-3	-3	-0.5	-1

ID	LEARN5	CAREER5	PEERRELAT	RESPECT5	SECURE5	PARTICI5	HORNOR5	CAREERfit	INCOMEfit	LEARNfit	BENEFITfi	PARTfit	RESPECTfi
511	2.5	4	4	4	5	4.5	2.5	-2	-1.5	-0.5	0.5	-2.5	0
512	4	4.5	4.5	4	4	3.5	3	-2.5	-3	0	-1	0.5	-1
513	5	5	4	4	5	4	5	-3	-4	-3	-3	-1	-1
514	5	5	3	2.5	3	1.5	3.5	-1	-3	-2	-0.5	2.5	1.5
515	5	4.5	4.5	5	4.5	4.5	4.5	-3.5	-3	-2	-2	-0.5	0
516	1	0.5	4	5	5	3.5	3.5	0.5	-2.5	3	-1	0.5	-2
517	3.5	2.5	4	4	4.5	4	4	0.5	1	0.5	1	0	0
518	4	3	4.5	3.5	4.5	3	2.5	-2	-0.5	-1	0	1	0.5
519	3.5	4	5	4.5	4	3	2.5	-1	0.5	-0.5	-1	1	-1.5
520	4	4	4.5	4	4	3.5	3.5	-1	-1	0	-0.5	0.5	0
521	4	3	5	4.5	4	4	4.5	-1	0.5	-1	0	-1	-1.5
522	2.5	3.5	5	5	3	5	5	-0.5	-0.5	0.5	1	0	-1
523	3	3	3	3	3	3	3	0	0	0	0	-1	1
524	5	4	5	5	5	5	5	0	-1	0	0	-1	-1
525	3.5	4.5	4	3	3.5	1.5	3.5	-0.5	-2	0.5	-1	2.5	1
526	3.5	3.5	3.5	3.5	3.5	3.5	3.5	-0.5	-0.5	0.5	0.5	0.5	-0.5
527	4	4	4	4	4	4	3	-2	-0.5	-1	-1	-2	-1
528	2.5	3.5	4.5	3	4	3.5	3	-0.5	-1	0.5	0	1.5	1
529	1.5	3	5	5	5	1	3.5	0	0.5	1.5	1.5	3	-1
530	5	4.5	5	4	5	4.5	4	-0.5	-2	-1	-1	-0.5	0
531	5	4	5	4	5	5	5	-1	-3	-1	-2	-1	0
532	4.5	5	5	5	5	4	4	-1	-2	0.5	-1	1	-1
533	5	5	5	5	5	5	5	-2	-3	-1	0	0	-1
534	2.5	0.5	5	5	2.5	2.5	2.5	2.5	0.5	1.5	0.5	0.5	-1
535	4.5	4.5	5	5	4.5	4.5	4	-3.5	-3	-1.5	-1	-2.5	-1
536	2.5	2.5	2.5	2.5	2.5	2.5	2.5	-1.5	0.5	0.5	0.5	-0.5	0.5
537	4	4	3.5	5	4	2.5	2.5	-2	0.5	0	-1	0.5	-2
538	3	4.5	2.5	1.5	0.5	1	1.5	-1.5	-2	0	-0.5	4	2.5
539	5	4.5	5	4.5	5	5	5	-0.5	-0.5	0	-0.5	0	0.5
540	3	2	3	3.5	4	4	2	-1	0.5	0	1.5	0	0.5
541	2.5	1.5	2	2.5	2.5	2	2.5	1.5	-0.5	0.5	0.5	2	0.5
542	5	5	5	5	5	5	5	-2	-3	-2	-1	0	-1
543	3.5	3.5	3.5	3	3.5	3.5	3	-0.5	-1.5	-0.5	0	1.5	1
544	5	5	5	4.5	4.5	2.5	5	0	-1	-1	0	2.5	-0.5

ID	LEARN5	CAREER5	PEERRELAT	RESPECT5	SECURE5	PARTICI5	HORNOR5	CAREERfit	INCOMEfit	LEARNfit	BENEFITfi	PARTfit	RESPECTfi
545	5	5	5	5	5	5	5	-2	-1	-1	-1	0	-1
546	2.5	2	4	5	5	3.5	5	-1	-0.5	0.5	0	-0.5	-2
547	1.5	1	2	3	2.5	1.5	1	2	0.5	2.5	0.5	2.5	1
548	4.5	5	5	5	4.5	4.5	4.5	-3	0	-0.5	-0.5	-2.5	-1
549	3.5	4.5	2.5	2	3	1	3.5	-0.5	-2	0.5	-1	3	2
550	3.5	2.5	3	3	3.5	3	3	0.5	0	-0.5	0	1	0
551	4.5	5	5	5	4.5	4.5	4.5	-2	-2.5	-1.5	-1.5	-0.5	-2
552	2	2	5	5	5	3	4	1	0.5	1	1.5	0	-1
553	3.5	3	5	4.5	5	4.5	4	0	0	-0.5	-0.5	-0.5	-0.5
554	4.5	4	4	4	4	4	4	-1	-1	-1.5	-1	0	-1
555	3.5	0.5	4.5	4.5	4.5	4	4	2.5	2.5	0.5	2.5	-1	-1.5
556	2.5	2.5	2.5	2.5	2.5	2.5	2.5	0.5	0.5	1.5	1.5	1.5	0.5
557	4	4	2.5	2.5	2.5	4	4	0	-0.5	0	1.5	0	1.5
558	4.5	0.5	4.5	4.5	4.5	0.5	3.5	0.5	2.5	-1.5	2.5	0.5	0.5
559	2	1	4.5	3	3	3.5	4	0	1.5	1	0.5	-0.5	0
560	2.5	1.5	5	4.5	3.5	4	3.5	1.5	-0.5	0.5	0.5	0	0.5
561	1	1	4	2.5	1.5	1	3	2	1	3	2.5	3	1.5
562	5	4	4	3.5	3.5	3	4.5	-1	-0.5	-2	-0.5	-1	0.5
563	5	5	3.5	3	2.5	3	1.5	-2	-3.5	-2	0	0	0
564	3	4	5	3.5	4	3.5	3.5	-1	-0.5	0	-1	0.5	-0.5
565	5	4	5	5	2.5	2.5	4	-1	-1	-1	0	-0.5	0
566	3.5	2.5	4	5	3.5	3.5	3	1.5	-0.5	0.5	0	0.5	0
567	3.5	2	4	4	4.5	3.5	2.5	1	-1	0.5	0	0.5	0
568	4.5	5	4	4	4.5	3.5	4	-2	-2	-3.5	-3	-0.5	-1
569	4.5	4	4.5	4.5	4.5	4	4	-1	-2	-1.5	-1	0	-0.5
570	5	5	3.5	4.5	5	4.5	4	-2	-2	-1	1.5	-1.5	-0.5
571	4	5	4	2.5	3	2	2	-2	-2	-1	-2	1	0.5
572	2.5	2.5	4	4	4.5	4.5	3	0.5	0.5	1.5	1	0.5	1
573	2	2	2.5	1.5	2	2	2.5	1	1.5	1	0.5	1	1.5
574	5	4.5	5	4.5	5	4	3	-0.5	-4	-2	0.5	0	-0.5
575	5	5	5	5	5	3	4	-2	-3	-2	-2	-1	-1
576	5	5	4	4.5	5	4	4.5	-2	-2	-1	-2	1	-0.5
577	5	4.5	3	2	2.5	2	3.5	-0.5	-1	-1	1	2	2
578	3	3.5	4	4	4	4	2.5	-0.5	0.5	1	0.5	0	0

ID	LEARN5	CAREER5	PEERRELAT	RESPECT5	SECURE5	PARTICI5	HORNOR5	CAREERfit	INCOMEfit	LEARNfit	BENEFITfi	PARTfit	RESPECTfi
579	4.5	4.5	4	3	2.5	2	2	-1.5	-2.5	-0.5	-1	2	2
580	5	4.5	5	3.5	2.5	4.5	3.5	-2.5	-2	-2	-3	-0.5	0.5
581	4	1	1.5	1.5	1	3	1.5	2	-1	-2	-3	1	2.5
582	5	4	4.5	5	4	4	3.5	-1	-2	-3	-2	0	-2
583	3.5	3	3.5	2.5	3	3.5	3	-1	0	-1.5	0	-1.5	0.5
584	3.5	4	2.5	2	2	0.5	2	-1	-1	0.5	0	3.5	2
585	5	5	3	3	4.5	2.5	3	-2	-1.5	-2	-1.5	0.5	1
586	3.5	4.5	3.5	3	3	2	3.5	-3.5	-2	-0.5	0	1	0
587	3	3	4.5	4.5	5	4	5	0	1	0	1.5	-1	-0.5
588	4.5	4.5	3	3.5	2	1	1.5	-1.5	-2	-0.5	-1.5	2	0.5
589	4.5	4.5	5	5	4.5	4.5	4.5	-2.5	-2	-1.5	-1.5	-2.5	-3
590	3	2	4	4.5	5	4	5	1	-0.5	0	0	-1	-1.5
591	4	5	3.5	4	3	1.5	3	-2	-1.5	-1	-1	2.5	0
592	5	4	4	4.5	5	5	4.5	-3	-2	-2	-1	-1	-1.5
593	2	3	5	4	4.5	3	4	0	-2.5	0	-2.5	-1	0
594	2	2.5	4	4	3	3	3.5	0.5	-0.5	1	0	1	0
595	1.5	2	4.5	5	4.5	4.5	4	-1	-1.5	-0.5	-2.5	-3.5	-1
596	1	2	5	4.5	3	1.5	3.5	1	1	2	1.5	2.5	-0.5
597	3.5	2.5	2.5	3	3	2.5	3.5	0.5	-2	-0.5	-3.5	2.5	0
598	5	4.5	5	4	5	3.5	4	-1.5	-1	0	0	1.5	1
599	4.5	4	4	4	4.5	4	4	-3	-2.5	-2.5	-3.5	-2	0
600	2.5	4.5	4.5	4.5	4.5	4.5	4.5	-1.5	-3.5	0.5	0.5	-2.5	-3.5
601	5	4.5	5	4.5	5	5	4	-0.5	-1	-1	0	-1	-1.5
602	3.5	4	3	3	2.5	1	3.5	-2	-3.5	-1.5	-3.5	0	1
603	3.5	5	1.5	1.5	1	3	1	-4	-1	-0.5	-1.5	0	2.5
604	4.5	4.5	4.5	5	4.5	4.5	4.5	-2.5	-2.5	-0.5	-1.5	-0.5	-2
605	2	4.5	4.5	4.5	4	4.5	3.5	-2.5	-2	2	0	-2.5	-1.5
606	3	5	4	3.5	3	3.5	3.5	-2	-1.5	1	-1	0.5	0.5
607	4	4	3	2.5	3	3	3.5	-3	-2	-2	-3	-2	1.5
608	3.5	3.5	3.5	3.5	3	4	4	-0.5	-1	0.5	-2	-2	0.5
609	3	3.5	2.5	3	1.5	1	1.5	-2.5	-0.5	-1	-1.5	0	1
610	4.5	4.5	4.5	5	4.5	4.5	4.5	-2.5	-2.5	-0.5	-1.5	-0.5	-2
611	4	2.5	3	3	2.5	2.5	2.5	0.5	-1.5	-1	0.5	0.5	1
612	3.5	4	3	3.5	2.5	1.5	3.5	-1	-2	-0.5	-0.5	1.5	-0.5

ID	LEARN5	CAREER5	PEERRELAT	RESPECT5	SECURE5	PARTICI5	HORNOR5	CAREERfit	INCOMEfit	LEARNfit	BENEFITfi	PARTfit	RESPECTfi
613	5	4	3.5	3	3.5	1	1	-1	-1.5	0	-2	1	1
614	3	1.5	4	3.5	3.5	3	1.5	-0.5	1	0	2	-1	0.5
615	3.5	2	3.5	3.5	3	4	4	0	-1	0.5	-2	-2	0.5
616	2.5	3	4.5	3.5	3	2.5	2.5	0	-1.5	0.5	0.5	0.5	1.5
617	4	2	4	4	4	2	2	0	1	-1	1	0	0
618	5	5	5	4.5	5	4	4	-1	-1	-2	-1	0	-0.5
619	2.5	2.5	4	3	2.5	2.5	2.5	0.5	0.5	0.5	0.5	0.5	1
620	4.5	3.5	5	4.5	5	4	4.5	-0.5	-2.5	-1.5	-0.5	0	0.5
621	4.5	4.5	5	5	5	4.5	4.5	-1.5	-0.5	0.5	0	-0.5	-1
622	4	4	4	5	2.5	3.5	3	-2	-0.5	-1	1	-0.5	-1
623	5	4	4.5	4.5	4	3.5	5	-3	-2	-4	-3	-1.5	-1.5
624	5	4.5	2.5	3	2	3	2	-1.5	-3.5	-2	-1.5	1	1
625	2.5	4	4	4	4.5	4	3.5	-1	2.5	1.5	1.5	-2	-1
626	3	2.5	5	5	4	5	5	0.5	0	0	0.5	0	-1
627	4	5	4	4	5	4	4	-2	-2	-1	-2	-2	0
628	3.5	4	4	3.5	3.5	4	4.5	-1	-1.5	0.5	-2.5	0	0.5
629	5	4	4	4	4	4	2.5	-3	-2	-2	-2	0	-1
630	4	5	4	4	5	4	5	-2	0	-1	-1	1	0
631	4	3	3	4	4	4	5	0	-1.5	-2	-3	-1	1
632	3.5	4	4	4	4	3	4	-1	0.5	0.5	-3	1	0
633	2.5	3.5	3.5	3.5	4	2.5	4	-0.5	0	1.5	1.5	1.5	0.5
634	4.5	4.5	4	4.5	4.5	4.5	4.5	-1.5	-1.5	-1.5	-0.5	-1.5	-0.5
635	5	5	5	5	5	5	4	-3	-2	-3	-3	-2	-2
636	1	4	2.5	4.5	3	1.5	2.5	-1	1.5	2	0.5	1.5	-0.5
637	3.5	4.5	4.5	4.5	4.5	3.5	4	-1.5	-1.5	0.5	0.5	0.5	-0.5
638	3	2.5	5	4	3	3	2.5	-0.5	1	0	-0.5	2	0
639	2	2.5	4.5	4.5	4	4	4.5	-0.5	0.5	1	-0.5	-2	-1.5
640	4	5	4.5	4	3.5	4	4	-4	-3	-2	-3	1	0
641	3	4	4.5	2	1	4	2.5	-1	0.5	0	2	0	1
642	5	5	5	5	3	3	4	-3	-1	-1	-3	-1	-1
643	4	4	2	3	4	4	3.5	-2	-1	-1	-1	-2	1
644	5	5	3	2.5	3.5	3.5	5	-3	-1.5	-2	-3	-0.5	1.5
645	1.5	2	3	2.5	4	3	3	0	-1	1.5	2	0	0.5
646	4	1.5	2	3	4	4	3	1.5	-1	-1	-0.5	-1	0

ID	LEARN5	CAREER5	PEERRELAT	RESPECT5	SECURE5	PARTICI5	HORNOR5	CAREERfit	INCOMEfit	LEARNfit	BENEFITfi	PARTfit	RESPECTfi
647	2.5	1.5	3	4	4	3	1	1.5	1.5	1.5	0	0	0
648	3	2.5	4	3	1.5	2.5	2.5	-0.5	0.5	0	-0.5	0.5	1
649	2.5	3	3	2.5	3	3	3.5	0	0.5	0.5	0.5	1	1.5
650	4	2.5	3	3.5	4	2.5	1	0.5	-2	0	-2	0.5	-0.5
651	5	5	5	5	5	4	5	-4	-2	0	-3	-2	-1
652	5	1.5	5	5	5	5	5	0.5	-2	-2	-2	-1	-2
653	1.5	4	3.5	1.5	1.5	3.5	2.5	-1	0.5	1.5	1.5	0.5	1.5
654	2.5	2	3	3	3	2.5	3	1	-0.5	0.5	0.5	0.5	1
655	3.5	5	3.5	4	4	3.5	3	-3	-1.5	-0.5	-1.5	0.5	0
656	3.5	4	2.5	4.5	4	4	2.5	-2	-1	-1.5	-3	-3	-1.5

ID	STABLEfit	RELATIONf	HORNORfit	POFIT	relatedfi	achvmntfitscore	
1	0.5	2.5	0.5	-0.16667	0.8	-1.375	
2	1.5	2	1	0.277778	0.6	-0.125	
3	1.5	-0.5	0.5	-0.16667	0.1	-0.5	
4	3.5	3	1	0.5	1.8	-1.125	
5	0	0	-1	0.444444	0	1	
6	0.5	1.5	1.5	0.222222	0.7	-0.375	
7	0	-1	0	-0.5	-0.4	-0.625	
8	-0.5	0	0.5	-0.16667	0.2	-0.625	
9	2.5	-1	1	-0.16667	0.9	-1.5	
10	1	0	-2	-1.5	-0.3	-3	
11	0	0	-1	-1.16667	-0.6	-1.875	
12	-1	0	0.5	0.277778	0.1	0.5	
13	-0.5	-1	-2	-1.33333	-1	-1.75	
14	1	1	1	0.666667	0.5	0.875	
15	0.5	1.5	2	-0.38889	0.8	-1.875	
16	-2	-3	-2	-1.72222	-1.9	-1.5	
17	1.5	-1	0	-0.27778	-0.2	-0.375	
18	1.5	0	0.5	0	0.3	-0.375	
19	0.5	1	-0.5	0.333333	0.5	0.125	
20	-1	0	0.5	-0.83333	0	-1.875	
21	1	2.5	1	1.166667	0.9	1.5	
22	0	2.5	1	0.555556	0.7	0.375	
23	0.5	-0.5	1	0.944444	0.5	1.5	
24	0	-0.5	1.5	0.055556	0	0.125	
25	-1	-1	-1	-1.66667	-1	-2.5	
26	1	-0.5	-1	-0.83333	-0.2	-1.625	
27	-1	0	-1	-1.22222	-0.4	-2.25	
28	-2	-1	-1	-1.77778	-1.3	-2.375	
29	0	0	-1	-0.16667	-0.5	0.25	
30	-1	-0.5	-2	-1.88889	-2.1	-1.625	
31	-1	-1.5	-2	-1.16667	-1.2	-1.125	
32	-0.5	0.5	0	0.277778	0.2	0.375	
33	1	0.5	1	-0.66667	0.3	-1.875	
34	0.5	-1	0.5	-0.5	0	-1.125	

ID	STABLEfit	RELATIONf	HORNORfit	POFIT	relatedfi	achvmntfitscore	
35	-2	-1	-0.5	-1.38889	-1	-1.875	
36	0.5	1	1.5	0.111111	0.9	-0.875	
37	0	-1.5	-0.5	-0.44444	-0.5	-0.375	
38	-0.5	0.5	-0.5	-0.33333	-0.4	-0.25	
39	-1	0	-0.5	-0.83333	-0.7	-1	
40	-1.5	-0.5	-2	-0.5	-0.7	-0.25	
41	0	0	-1	-0.5	-0.3	-0.75	
42	-1	0.5	-1	-0.88889	-0.7	-1.125	
43	0	0	-1	-0.77778	-0.4	-1.25	
44	1	0.5	-1	0.222222	0.4	0	
45	-2	0	-1	-2.22222	-2	-2.5	
46	-1.5	-0.5	-1.5	-1.16667	-0.9	-1.5	
47	-1	2	1	-0.22222	0.6	-1.25	
48	2	1.5	0	-0.16667	0.8	-1.375	
49	-1	-1	0	-0.05556	-0.2	0.125	
50	1.5	2.5	0	0.111111	0.9	-0.875	
51	-1.5	1.5	2.5	-0.22222	0.7	-1.375	
52	0.5	-0.5	-2	-1.27778	-0.7	-2	
53	1	0.5	1	-0.38889	0.3	-1.25	
54	-0.5	-1	-0.5	0	-0.4	0.5	
55	-1	0	-0.5	-0.5	-0.1	-1	
56	0	-2	-0.5	-0.61111	-0.8	-0.375	
57	0	0.5	-1.5	-0.61111	-0.3	-1	
58	0.5	0	-1	-0.11111	0.1	-0.375	
59	2.5	1.5	1.5	1.166667	1.5	0.75	
60	3	0.5	-0.5	-0.33333	0.6	-1.5	
61	1.5	0.5	1	0.055556	1.3	-1.5	
62	-1.5	0.5	-0.5	-1.22222	-0.7	-1.875	
63	-0.5	0	0	-0.88889	-0.3	-1.625	
64	-0.5	-0.5	0.5	0.722222	0.1	1.5	
65	0	1.5	0.5	1.111111	0.3	2.125	
66	-1	0.5	-1	-0.83333	-0.7	-1	
67	-1	0	1	-0.16667	0.1	-0.5	
68	0	0	-1	-0.38889	-0.4	-0.375	

ID	STABLEfit	RELATIONf	HORNORfit	POFIT	relatedfi	achvmntfitscore	
69	-2	0	-0.5	-1.16667	-0.6	-1.875	
70	-0.5	4.5	-1.5	-0.33333	0.5	-1.375	
71	-1	-1	-0.5	-0.55556	-0.6	-0.5	
72	0.5	0.5	-1	-0.55556	-0.1	-1.125	
73	0	0	0.5	-0.44444	-0.1	-0.875	
74	1	0	0.5	-0.05556	0	-0.125	
75	0.5	-0.5	0.5	-0.33333	-0.2	-0.5	
76	0	0.5	0.5	-0.05556	0.1	-0.25	
77	0	0	-1	-0.33333	-0.5	-0.125	
78	0.5	0	1.5	0	0.5	-0.625	
79	0.5	1.5	-0.5	-0.33333	0.5	-1.375	
80	0.5	0	-0.5	0.611111	0	1.375	
81	0.5	0	0.5	0.388889	0.5	0.25	
82	0	1	0	0.5	0.5	0.5	
83	-1	1	-0.5	-1.22222	-0.6	-2	
84	-1	1	0.5	-0.5	-0.1	-1	
85	1.5	0	2.5	0.222222	0.6	-0.25	
86	-1	0	0	-0.66667	-0.7	-0.625	
87	1.5	0	-0.5	-0.05556	0.4	-0.625	
88	1.5	1.5	0.5	-0.11111	0.9	-1.375	
89	0	0	0.5	-0.16667	-0.3	0	
90	-1	0	-3	-1.16667	-1.4	-0.875	
91	1	0	-1	-1.05556	-0.2	-2.125	
92	0	0.5	-1	-0.94444	-0.3	-1.75	
93	0.5	1.5	-0.5	-0.5	0.1	-1.25	
94	0.5	2.5	0.5	0.166667	1.1	-1	
95	-2	0	1	-0.61111	-0.6	-0.625	
96	-1	-1	-1	0.055556	-0.8	1.125	
97	-1	0	-1	-0.88889	-0.7	-1.125	
98	-2	-0.5	-2	-1.94444	-1.8	-2.125	
99	1	2	2.5	0.277778	1.5	-1.25	
100	-3	-0.5	-0.5	-1.88889	-1.4	-2.5	
101	-0.5	0	-2	-1.66667	-1.4	-2	
102	0.5	0.5	0	-0.83333	0.3	-2.25	

ID	STABLEfit	RELATIONf	HORNORfit	POFIT	relatedfi	achvmntfitscore	
103	0.5	-1	-1	-1.05556	-0.4	-1.875	
104	0	-1	-1.5	-0.22222	-0.6	0.25	
105	-1	1	0	-0.16667	0	-0.375	
106	0	0	0	-0.55556	-0.2	-1	
107	1	1	1.5	0.166667	0.8	-0.625	
108	1.5	1	-0.5	-0.05556	0.5	-0.75	
109	1	0	-1.5	-0.77778	-0.4	-1.25	
110	0.5	0	-0.5	-1.33333	-0.2	-2.75	
111	0	0	0	-0.11111	0.3	-0.625	
112	1	-1	-1	0.055556	-0.3	0.5	
113	0	1	0.5	-0.38889	0.3	-1.25	
114	1.5	2	1.5	0.611111	2.1	-1.25	
115	-1	0	-2	-1.55556	-0.8	-2.5	
116	0.5	1.5	1.5	-0.27778	0.6	-1.375	
117	0.5	0.5	0.5	-0.16667	-0.1	-0.25	
118	-1	0	-2	-0.88889	-1.4	-0.25	
119	0.5	1.5	1.5	-0.22222	0.6	-1.25	
120	1.5	1	0.5	-0.11111	1.2	-1.75	
121	-3	-1	-2	-1.05556	-1.8	-0.125	
122	-0.5	2	0	-0.55556	0.3	-1.625	
123	1	1	0	-0.16667	0.9	-1.5	
124	0.5	1.5	0.5	1.222222	1.5	0.875	
125	-1	1.5	0	-0.11111	0.4	-0.75	
126	0.5	0.5	1.5	0.333333	1.1	-0.625	
127	1.5	0	0	-0.55556	0.1	-1.375	
128	1.5	0	-0.5	0	0.1	-0.125	
129	0.5	1.5	1	0.722222	1.4	-0.125	
130	1	2	1.5	1.5	1.9	1	
131	-2	-0.5	-1	-1	-0.7	-1.375	
132	-1	1.5	-2	-0.88889	-0.2	-1.75	
133	-1	0.5	-2	-1	-0.5	-1.625	
134	-0.5	0.5	-2	-0.88889	-0.4	-1.5	
135	-1	0	0	-0.22222	0.3	-0.875	
136	-0.5	-0.5	-1.5	-1.38889	-0.8	-2.125	

ID	STABLEfit	RELATIONf	HORNORfit	POFIT	relatedfi	achvmntfitscore	
137	0.5	1	1.5	0.222222	1.3	-1.125	
138	-1	0.5	-1.5	-1.94444	-1.3	-2.75	
139	3	1	3.5	0.722222	2.7	-1.75	
140	0	1	0.5	-0.66667	0.7	-2.375	
141	0	-0.5	-0.5	0.388889	-0.4	1.375	
142	0.5	0	0	0.944444	0.2	1.875	
143	-1	0	-1	-1.22222	-0.6	-2	
144	-2	0	-1	-1.5	-1.1	-2	
145	-1	0	1	-0.72222	-0.2	-1.375	
146	-1	0	0	-1.11111	-0.7	-1.625	
147	-1	2	2	-0.88889	0.4	-2.5	
148	0	0	0	-0.22222	0	-0.5	
149	-1	0	-1	0.166667	0.2	0.125	
150	1	1.5	0.5	0.444444	1.3	-0.625	
151	-0.5	0	0.5	-0.61111	-0.6	-0.625	
152	-1.5	0	0.5	-0.27778	0	-0.625	
153	2.5	0	0.5	0.5	0.9	0	
154	0	-1	-1	-0.77778	-0.4	-1.25	
155	-0.5	0	-2.5	-0.55556	-0.8	-0.25	
156	2.5	2.5	-0.5	0.055556	0.8	-0.875	
157	1.5	-0.5	1.5	0.5	0.8	0.125	
158	0	-1	-1	-0.55556	-0.4	-0.75	
159	3.5	0	2.5	1.055556	2.4	-0.625	
160	3.5	2.5	1.5	1.166667	2.4	-0.375	
161	-0.5	-0.5	0	-0.22222	-0.4	0	
162	0.5	0	-0.5	-0.61111	-0.3	-1	
163	3	3	3.5	2.666667	2.9	2.375	
164	-0.5	-0.5	-1	-0.5	-0.8	-0.125	
165	-1.5	0.5	0.5	-0.05556	0.1	-0.25	
166	0.5	1.5	0.5	0.666667	0.9	0.375	
167	-1	-1	0	-1.05556	-1.1	-1	
168	0.5	0	0.5	-0.22222	0.1	-0.625	
169	1	1.5	0	0	0.5	-0.625	
170	0.5	1	0.5	0.055556	0.5	-0.5	

ID	STABLEfit	RELATIONf	HORNORfit	POFIT	relatedfi	achvmntfitscore	
171	0	0.5	0.5	-0.05556	0.6	-0.875	
172	0.5	-0.5	-3	-0.61111	-1.1	0	
173	1.5	1.5	2.5	1.222222	1.7	0.625	
174	0	2.5	1	-0.16667	0.9	-1.5	
175	1.5	0.5	0	0.111111	0.6	-0.5	
176	-2	0	-0.5	-1.66667	-1.1	-2.375	
177	1	3	-0.5	-0.88889	0.4	-2.5	
178	-1	1	0	0.111111	-0.1	0.375	
179	-2	-0.5	0	-0.5	-1.1	0.25	
180	0.5	-3	0	-0.61111	-0.3	-1	
181	-0.5	-0.5	-0.5	-0.55556	-0.5	-0.625	
182	-1	0.5	-0.5	-0.44444	0	-1	
183	-1	0	0	-0.27778	-1.2	0.875	
184	-2	-1	-0.5	-0.94444	-1.2	-0.625	
185	-2.5	-0.5	-0.5	-1.44444	-1.5	-1.375	
186	-0.5	-1	-1.5	-1.11111	-0.8	-1.5	
187	-2	1	-0.5	0.333333	-0.2	1	
188	0	0	-1	-0.72222	-0.3	-1.25	
189	1	0	-0.5	0.555556	0.5	0.625	
190	-2	-1	-0.5	-0.5	-0.4	-0.625	
191	-1	0	-3	-1.72222	-1.7	-1.75	
192	0	0	-2	-0.11111	-0.4	0.25	
193	0	1.5	0	0.055556	0.3	-0.25	
194	1.5	-0.5	-0.5	-1.22222	-0.1	-2.625	
195	1	-0.5	0.5	-1.27778	0.2	-3.125	
196	-0.5	0	2	-0.22222	0.4	-1	
197	-1	0	-3	-0.77778	-1	-0.5	
198	2	1.5	-0.5	-1	0.2	-2.5	
199	-1	1	-2	-0.05556	-1.2	1.375	
200	0.5	1	0.5	-0.94444	0.5	-2.75	
201	0.5	1.5	-1	-0.94444	0.1	-2.25	
202	-0.5	-0.5	0	0.111111	-0.1	0.375	
203	-1.5	-0.5	1.5	-0.33333	-0.3	-0.375	
204	1.5	0.5	-1	1.444444	1	2	

ID	STABLEfit	RELATIONf	HORNORfit	POFIT	relatedfi	achvmntfitscore	
205	1.5	1.5	3	1.555556	1.8	1.25	
206	0.5	0	0	1	0.5	1.625	
207	2.5	0.5	1.5	0.555556	1.9	-1.125	
208	-0.5	1.5	-0.5	-1	0.6	-3	
209	1	1	-0.5	0.222222	0.8	-0.5	
210	-1	-1	-3	-1.55556	-0.7	-2.625	
211	1	1	1	0.055556	1	-1.125	
212	-0.5	1.5	1.5	1	0.9	1.125	
213	-3	0	0	-0.44444	-0.3	-0.625	
214	0	-2	-1	-0.16667	-0.5	0.25	
215	0	1.5	0	-0.16667	0.3	-0.75	
216	2.5	2	2.5	1.277778	1.9	0.5	
217	0	0.5	-1	0.166667	-0.4	0.875	
218	3.5	2	-0.5	0.722222	2	-0.875	
219	0	0	-0.5	-1.55556	-0.6	-2.75	
220	0	0.5	0	-0.16667	0.5	-1	
221	0.5	0	-1	-1	-0.7	-1.375	
222	0	0	0.5	-0.94444	0.5	-2.75	
223	1.5	-1	-2	-1.11111	-0.4	-2	
224	0.5	-1	-2	-1.05556	-0.6	-1.625	
225	0.5	-0.5	-1	-1.16667	-0.5	-2	
226	-3	-1	-3	-2.66667	-2.2	-3.25	
227	0.5	0	1	1	0.4	1.75	
228	3.5	2.5	2.5	0.111111	2.2	-2.5	
229	0.5	1	0	-0.72222	0.1	-1.75	
230	0.5	-0.5	0	-0.83333	-0.3	-1.5	
231	-0.5	0	0	-0.88889	0.3	-2.375	
232	0	1	2.5	0.166667	1.5	-1.5	
233	1.5	2.5	2.5	0.388889	2	-1.625	
234	0.5	3	1	-0.05556	0.9	-1.25	
235	-3	0.5	-4	-1.77778	-1.7	-1.875	
236	0	0	2	1	0.7	1.375	
237	-2	1	-3	-1.72222	-0.7	-3	
238	-0.5	0.5	-0.5	-0.38889	-0.1	-0.75	

ID	STABLEfit	RELATIONf	HORNORfit	POFIT	relatedfi	achvmntfitscore	
239	-0.5	0	-0.5	-0.11111	0.2	-0.5	
240	-2	1	-3	-1.77778	-1.9	-1.625	
241	-0.5	0.5	0	0.333333	0	0.75	
242	0	2.5	1	-0.11111	0.7	-1.125	
243	0.5	2	-0.5	-0.88889	0.7	-2.875	
244	-1	-1	-0.5	-1.72222	-1.1	-2.5	
245	-2	0	-2	-1.61111	-1.4	-1.875	
246	-1	0	-1.5	-1.33333	-0.9	-1.875	
247	0	0	-0.5	-1	-0.3	-1.875	
248	-0.5	0	-1.5	-1.22222	-0.3	-2.375	
249	1	1.5	1	0.666667	1	0.25	
250	0	0.5	-1	-0.5	-0.1	-1	
251	-1.5	0.5	-2	-0.72222	-0.7	-0.75	
252	-1	1	-1	-0.61111	-0.4	-0.875	
253	0.5	-0.5	0	0.055556	-0.1	0.25	
254	-0.5	-1.5	-2	-0.55556	-0.8	-0.25	
255	0	1	-0.5	-0.55556	0	-1.25	
256	3	1	-0.5	0.111111	1.3	-1.375	
257	2	0.5	0	-0.44444	0.4	-1.5	
258	3	2.5	2.5	1.555556	2.8	0	
259	2.5	0.5	1.5	0.611111	1.3	-0.25	
260	3	1	1	0.5	1.4	-0.625	
261	-1	-1.5	-2	-1.72222	-1.4	-2.125	
262	-1	1	-1	-0.77778	-0.8	-0.75	
263	2.5	2.5	2	0.611111	2	-1.125	
264	2.5	0	3.5	0.722222	1.8	-0.625	
265	0.5	2.5	0.5	-0.27778	0.3	-1	
266	0	0	0	-0.22222	0.2	-0.75	
267	0	0	-1	-1.44444	-0.8	-2.25	
268	0	1	1.5	-0.33333	0.5	-1.375	
269	-1	-1	-1	-1.72222	-1.3	-2.25	
270	-1	-1	-1	-0.61111	-0.7	-0.5	
271	-0.5	1	-2	-0.88889	-0.7	-1.125	
272	-0.5	0	0.5	0.111111	0.1	0.125	

ID	STABLEfit	RELATIONf	HORNORfit	POFIT	relatedfi	achvmntfitscore	
273	-1	0	-0.5	-0.5	-0.3	-0.75	
274	1	2	0.5	0.388889	1.4	-0.875	
275	-1	-0.5	-0.5	-1.22222	-0.6	-2	
276	0	0	-1	0	-0.2	0.25	
277	2.5	1.5	0	-0.27778	0.3	-1	
278	2.5	1.5	-0.5	-0.38889	0.4	-1.375	
279	-2.5	0	-1.5	-1.61111	-1.2	-2.125	
280	0	0	0	-0.16667	0	-0.375	
281	2	0.5	3.5	1.055556	2.4	-0.625	
282	0	1	-0.5	-1.05556	-0.2	-2.125	
283	0	1	-1	-0.66667	-0.2	-1.25	
284	-0.5	0.5	1	-0.33333	0.6	-1.5	
285	0	0.5	0.5	-0.05556	0.3	-0.5	
286	0	1	1.5	0.777778	1	0.5	
287	-0.5	0	0	0	-0.2	0.25	
288	0	0	1.5	0.944444	1.1	0.75	
289	0.5	0	0.5	-0.11111	-0.3	0.125	
290	0.5	0	0	0	0.1	-0.125	
291	0.5	0	-1	-0.94444	-0.5	-1.5	
292	-0.5	0	-0.5	-0.27778	-0.1	-0.5	
293	-1	1	-0.5	0.333333	0.2	0.5	
294	0	0	0	-0.05556	0.2	-0.375	
295	-0.5	0.5	-2	-0.72222	-0.7	-0.75	
296	0	1	0	0.388889	-0.2	1.125	
297	-0.5	0	-2	-0.38889	-0.7	0	
298	-1	1	-1	-0.77778	-0.8	-0.75	
299	0	0.5	-1	-0.77778	0.1	-1.875	
300	1.5	1.5	0.5	0.277778	0.9	-0.5	
301	1	0.5	0	0.111111	1	-1	
302	-1.5	1	-0.5	-0.88889	-0.5	-1.375	
303	-0.5	-0.5	-0.5	-0.72222	-0.5	-1	
304	-1	1	1	-0.61111	0.3	-1.75	
305	0.5	0	1	-0.22222	0.6	-1.25	
306	-0.5	0.5	-1.5	-1.16667	-0.7	-1.75	

ID	STABLEfit	RELATIONf	HORNORfit	POFIT	relatedfi	achvmntfitscore	
307	1	1	1	0.333333	1	-0.5	
308	0.5	-0.5	1	0.333333	0.4	0.25	
309	-0.5	-1.5	-1.5	-1.22222	-1.5	-0.875	
310	0	-4	-1	-1.22222	-1.4	-1	
311	2.5	0.5	1	-0.16667	0.8	-1.375	
312	1	2	0.5	0.055556	1.3	-1.5	
313	0.5	1	1.5	-0.16667	0.9	-1.5	
314	-1	1	-2	-0.77778	-0.8	-0.75	
315	-0.5	0	-1	-0.61111	-0.3	-1	
316	-1	-1	0	-1	-0.4	-1.75	
317	0	1	-0.5	-0.05556	0.3	-0.5	
318	-1	-1	-1	-1.05556	-1	-1.125	
319	1	-1	-1	-0.72222	-0.6	-0.875	
320	0	0	-1	-1.05556	-0.7	-1.5	
321	2	0.5	0.5	0.388889	1.2	-0.625	
322	0	-1	-0.5	-1.33333	-0.9	-1.875	
323	1.5	1	-1	0.111111	0.8	-0.75	
324	1.5	0.5	0	-0.61111	0.3	-1.75	
325	0.5	0	0.5	-0.11111	0.5	-0.875	
326	0	-1	0	-0.33333	-0.2	-0.5	
327	0	0	-1	-0.33333	0	-0.75	
328	0	0	0.5	-0.38889	0	-0.875	
329	0	0.5	-0.5	0.111111	0	0.25	
330	-1	-1.5	0	-1.11111	-0.8	-1.5	
331	1	1	0	-0.77778	0.3	-2.125	
332	0.5	0	0.5	1.055556	0.7	1.5	
333	1	2.5	2.5	0.666667	2.1	-1.125	
334	0	1	0	0.111111	0.2	0	
335	0	0	0	0.222222	0.1	0.375	
336	0	-1	-1	-1.33333	-0.8	-2	
337	-0.5	-0.5	0	0.333333	-0.2	1	
338	2.5	2	1	0.722222	1.7	-0.5	
339	-1	-1	0	-1.11111	-0.6	-1.75	
340	0	1	0	-0.72222	-0.4	-1.125	

ID	STABLEfit	RELATIONf	HORNORfit	POFIT	relatedfi	achvmntfitscore	
341	0	-1	-1.5	-2	-1.2	-3	
342	0	0	0	-0.16667	0.1	-0.5	
343	-0.5	-0.5	-1.5	-1.5	-0.7	-2.5	
344	-1	1.5	0.5	0.722222	0.5	1	
345	-0.5	0	0	-1	-0.4	-1.75	
346	-1	0	0	-0.55556	-0.2	-1	
347	-1	0.5	1.5	-1	-0.5	-1.625	
348	-1	0	-1	-0.61111	-0.6	-0.625	
349	-1	0	-1.5	-0.77778	-0.7	-0.875	
350	0.5	-0.5	2	0.388889	0.4	0.375	
351	-0.5	1.5	3.5	1.333333	1.3	1.375	
352	-1	0	-2	0.222222	-0.9	1.625	
353	-0.5	-0.5	-1.5	-0.44444	-0.8	0	
354	0	1.5	1.5	0.222222	1.5	-1.375	
355	3.5	3.5	2	1.444444	2.9	-0.375	
356	-0.5	-1	-3	-1.83333	-1.7	-2	
357	0	0	0.5	0.166667	0	0.375	
358	0.5	1.5	1.5	-0.16667	0.8	-1.375	
359	-1	-1	-0.5	-1.61111	-0.7	-2.75	
360	-1.5	-0.5	0	-1.38889	-0.7	-2.25	
361	-0.5	-1	-3	-2.16667	-1.5	-3	
362	0.5	0.5	1	-0.38889	0.5	-1.5	
363	1	0.5	-0.5	-0.94444	0.2	-2.375	
364	-0.5	-0.5	1	-0.38889	0.2	-1.125	
365	0.5	1.5	-0.5	-0.72222	0.5	-2.25	
366	2	2	2.5	0.388889	1.6	-1.125	
367	2	0	0.5	-0.05556	1	-1.375	
368	1	1.5	1	-0.27778	0.7	-1.5	
369	-2	2	1.5	-0.16667	0.5	-1	
370	1.5	0	0	1.111111	1.1	1.125	
371	-1	1	0	-0.27778	0.3	-1	
372	-0.5	0.5	1.5	0.222222	0.5	-0.125	
373	0.5	0	1.5	0.833333	0.7	1	
374	0.5	0	0	-1	-0.6	-1.5	

ID	STABLEfit	RELATIONf	HORNORfit	POFIT	relatedfi	achvmntfitscore	
375	0	0	0	-0.11111	0	-0.25	
376	-0.5	0.5	1.5	0.277778	0.5	0	
377	0.5	1	0	0.444444	0.5	0.375	
378	0.5	0.5	1	-0.38889	0.6	-1.625	
379	-0.5	-1	-2	-1.44444	-1	-2	
380	0	0	0.5	-0.27778	0.4	-1.125	
381	2.5	-1	1.5	0.166667	0.9	-0.75	
382	1.5	0	0.5	-0.16667	1.2	-1.875	
383	0	0.5	0	-0.88889	-0.1	-1.875	
384	1.5	2.5	1	0.055556	1.1	-1.25	
385	-2	-2	-0.5	-0.66667	-1	-0.25	
386	-0.5	0	0	-0.44444	-0.1	-0.875	
387	0	0	0.5	-0.77778	0.2	-2	
388	-2	0	0	-0.5	-0.9	0	
389	1.5	2	1.5	0.888889	1.5	0.125	
390	-0.5	-1	-0.5	-0.72222	-0.8	-0.625	
391	-1.5	0	0	-0.88889	-0.4	-1.5	
392	0	2	-0.5	0.055556	1	-1.125	
393	-2	-0.5	-2	-0.61111	-1	-0.125	
394	0.5	1	0.5	0.944444	0.3	1.75	
395	2	1	0	1.166667	1.3	1	
396	0	-1	-1	-0.66667	-0.8	-0.5	
397	-1	-1	-1.5	-0.72222	-0.7	-0.75	
398	1.5	-1	0.5	-0.66667	0	-1.5	
399	1	-0.5	-1.5	-0.66667	-0.6	-0.75	
400	-1	-1	0	-0.77778	-0.7	-0.875	
401	0.5	0	-2	-1.05556	-0.7	-1.5	
402	3	4	2.5	1.277778	2.7	-0.5	
403	-0.5	0	2.5	0.722222	0.6	0.875	
404	-1	0	-1	-1.16667	-0.6	-1.875	
405	0.5	0	-0.5	0.111111	0	0.25	
406	-1	-1	-1.5	-1.44444	-1.3	-1.625	
407	2.5	2.5	2.5	0.277778	1.7	-1.5	
408	-1	0.5	-2	-0.22222	-0.3	-0.125	

ID	STABLEfit	RELATIONf	HORNORfit	POFIT	relatedfi	achvmntfitscore	
409	-1	-0.5	-1.5	-1.16667	-1	-1.375	
410	-1	0	-2	-1.33333	-1	-1.75	
411	0.5	0.5	0	-0.55556	0	-1.25	
412	-2	0	-2	-1.66667	-1.2	-2.25	
413	0	1	0	0.222222	0.4	0	
414	-2	-1	-2	-1.38889	-1.5	-1.25	
415	0	-0.5	-1	-1.05556	-0.6	-1.625	
416	-1	0	-1	-0.66667	-0.7	-0.625	
417	2	0	1	-0.22222	0.8	-1.5	
418	-0.5	-1	-0.5	-0.77778	-0.6	-1	
419	0.5	1	2.5	0.5	0.9	0	
420	1.5	2.5	1.5	1.277778	1.7	0.75	
421	-2	-1.5	-1	-1.55556	-1	-2.25	
422	-0.5	-1	0	-0.77778	-0.5	-1.125	
423	-0.5	0	0	-0.38889	0	-0.875	
424	-1	-1	-1	-0.88889	-1	-0.75	
425	-1	1.5	-3	-1.5	-0.8	-2.375	
426	0.5	-1	-0.5	0.277778	0.1	0.5	
427	-0.5	0	-1	-0.5	-0.3	-0.75	
428	0	1.5	0	-0.05556	0.3	-0.5	
429	1.5	0	0.5	0.055556	0.9	-1	
430	0	0	-1	-0.38889	-0.3	-0.5	
431	0	-1	0	0.166667	-0.4	0.875	
432	-0.5	-0.5	-0.5	-0.83333	-0.7	-1	
433	1.5	0	-0.5	0.722222	0.3	1.25	
434	0	0	0	0.111111	0.1	0.125	
435	1	2	2.5	0.166667	1.4	-1.375	
436	2	1.5	1.5	0.833333	1.6	-0.125	
437	0	-0.5	-1	-0.66667	-0.5	-0.875	
438	0	1	-1	-0.22222	0	-0.5	
439	-3	0.5	0	-0.77778	-0.4	-1.25	
440	0.5	-0.5	0	0.166667	0.4	-0.125	
441	-0.5	0	-1	-0.27778	-0.5	0	
442	-1	-0.5	-0.5	-1.11111	-0.5	-1.875	

ID	STABLEfit	RELATIONf	HORNORfit	POFIT	relatedfi	achvmntfitscore	
443	-3	0	-1	-1.44444	-1.4	-1.5	
444	3.5	3.5	3.5	1.277778	3.1	-1	
445	0	1	0.5	0.055556	0	0.125	
446	-2	0	0.5	-0.5	-0.4	-0.625	
447	1	0.5	0.5	0	1	-1.25	
448	-4	-1	0	-1.66667	-1.4	-2	
449	0.5	0	-0.5	-0.55556	0.2	-1.5	
450	-1.5	0	-0.5	-0.77778	-0.5	-1.125	
451	0	1.5	-1	0.388889	0.8	-0.125	
452	-1	0	-0.5	-1.11111	-0.8	-1.5	
453	-1	-1	-1.5	-0.27778	-0.8	0.375	
454	-1	-1	-1	-1.11111	-0.6	-1.75	
455	-2	0	-0.5	-0.55556	-0.7	-0.375	
456	2.5	1	0	1.444444	1.2	1.75	
457	0	2	1	0.444444	1	-0.25	
458	-1	0	1.5	-0.22222	0	-0.5	
459	0	-0.5	-2	-1.11111	-0.7	-1.625	
460	0	1.5	2.5	1.222222	1	1.5	
461	0.5	1	1	0.611111	1	0.125	
462	-1	0	-1	-1.44444	-0.8	-2.25	
463	0	-3	-0.5	-0.16667	-0.8	0.625	
464	0.5	0.5	1.5	0.111111	0.4	-0.25	
465	-0.5	1	0	-0.66667	0	-1.5	
466	0	1	0	-0.88889	0.1	-2.125	
467	0	0	0.5	0.222222	0.4	0	
468	0.5	1	0	-0.11111	0.8	-1.25	
469	0	1	1	0.555556	0.8	0.25	
470	-1	-1.5	0.5	-0.44444	-0.7	-0.125	
471	-1	0	-1.5	-0.5	-1.1	0.25	
472	-1	1	0	-0.22222	0.6	-1.25	
473	-1	0	1.5	-0.27778	0.4	-1.125	
474	-0.5	-0.5	-1	-0.83333	-0.5	-1.25	
475	-1.5	-1	-1	-1.22222	-0.7	-1.875	
476	0	-1	-2	-1.66667	-1.2	-2.25	

ID	STABLEfit	RELATIONf	HORNORfit	POFIT	relatedfi	achvmntfitscore	
477	1.5	0.5	-0.5	0.5	0.5	0.5	
478	-1	0	0	-0.5	-0.1	-1	
479	-1	0	-1.5	-1.16667	-1	-1.375	
480	1	4	3.5	1.277778	3	-0.875	
481	-1	1	-0.5	-0.38889	0.3	-1.25	
482	3.5	1.5	2	0.833333	2.3	-1	
483	0	1.5	0	-0.88889	0.6	-2.75	
484	0	-1	0.5	0.388889	-0.1	1	
485	-1.5	-0.5	-2	-1.05556	-0.9	-1.25	
486	-3	-0.5	-0.5	-1.83333	-1.3	-2.5	
487	-1.5	0	-1	-1.16667	-0.6	-1.875	
488	0.5	0.5	1	1.055556	0.8	1.375	
489	0	1	-3	-0.94444	0	-2.125	
490	0	1	0.5	-0.77778	0.4	-2.25	
491	-0.5	-1	-2.5	-1.66667	-1.6	-1.75	
492	3	-2	0.5	-0.72222	0.3	-2	
493	-1	0	-0.5	-0.16667	-0.2	-0.125	
494	2	0	1	0.722222	0.7	0.75	
495	-4	0	-1	-2.44444	-1.8	-3.25	
496	2	-1	0	0.722222	0.5	1	
497	1.5	0	1.5	0.944444	0.7	1.25	
498	-1	0	-1	-0.72222	-0.8	-0.625	
499	0	1	0	-0.16667	0.4	-0.875	
500	0	0	1	-0.27778	-0.1	-0.5	
501	1.5	1.5	-2.5	-1.16667	-0.1	-2.5	
502	0	1	2.5	-0.44444	0.5	-1.625	
503	-3	1	-0.5	-0.77778	-0.3	-1.375	
504	0.5	1.5	1	-0.11111	0.9	-1.375	
505	0	1	0.5	-0.88889	0.3	-2.375	
506	2.5	1.5	2.5	-0.05556	1.9	-2.5	
507	-0.5	0	1	-0.55556	0.4	-1.75	
508	-1.5	2	1.5	0.444444	0	1	
509	0.5	0.5	-0.5	-0.83333	0	-1.875	
510	-2	-1	-0.5	-1.88889	-1	-3	

ID	STABLEfit	RELATIONf	HORNORfit	POFIT	relatedfi	achvmntfitscore	
511	-1	0	-0.5	-0.83333	-0.8	-0.875	
512	0	-0.5	0	-0.83333	-0.2	-1.625	
513	-2	-1	-3	-2.33333	-1.6	-3.25	
514	1	1	0.5	0	1.3	-1.625	
515	-1.5	0.5	-1.5	-1.5	-0.6	-2.625	
516	-1	1	0.5	-0.11111	-0.2	0	
517	0.5	1	-1	0.388889	0.1	0.75	
518	-0.5	-1.5	0.5	-0.38889	0	-0.875	
519	-1	-1	0.5	-0.44444	-0.4	-0.5	
520	-1	0.5	0.5	-0.22222	0.1	-0.625	
521	0	-1	-1.5	-0.72222	-1	-0.375	
522	2	-1	-1	-0.05556	-0.2	0.125	
523	0	0	0	0	0	0	
524	-1	-1	-1	-0.66667	-1	-0.25	
525	0.5	0	-0.5	0.055556	0.7	-0.75	
526	-0.5	-0.5	0.5	-0.05556	-0.1	0	
527	0	0	1	-0.72222	-0.4	-1.125	
528	0	0.5	1	0.333333	0.8	-0.25	
529	-1	0	-0.5	0.444444	0.1	0.875	
530	-1	0	-1	-0.77778	-0.5	-1.125	
531	-1	0	-1	-1.11111	-0.6	-1.75	
532	0	0	0	-0.38889	0	-0.875	
533	-1	-1	-2	-1.22222	-1	-1.5	
534	1.5	0	0.5	0.722222	0.3	1.25	
535	-0.5	-1	-1	-1.66667	-1.2	-2.25	
536	-0.5	1.5	0.5	0.166667	0.3	0	
537	0	0.5	-0.5	-0.44444	-0.3	-0.625	
538	3.5	2.5	2.5	1.222222	3	-1	
539	-1	0	0	-0.22222	-0.1	-0.375	
540	-1	1	1	0.277778	0.3	0.25	
541	1.5	1	0.5	0.833333	1.1	0.5	
542	-1	0	-1	-1.22222	-0.6	-2	
543	0.5	0.5	1	0.222222	0.9	-0.625	
544	0.5	0	0	0.055556	0.5	-0.5	

ID	STABLEfit	RELATIONf	HORNORfit	POFIT	relatedfi	achvmntfitscore	
545	-1	0	-1	-0.88889	-0.6	-1.25	
546	-1	1	-3	-0.72222	-1.1	-0.25	
547	1.5	2	2	1.611111	1.8	1.375	
548	-1.5	-1	-1.5	-1.27778	-1.5	-1	
549	1	2.5	1.5	0.777778	2	-0.75	
550	0.5	0	0	0.166667	0.3	0	
551	-0.5	-2	-1.5	-1.55556	-1.3	-1.875	
552	-1	-1	0	0.111111	-0.6	1	
553	-1	-1	-1	-0.55556	-0.8	-0.25	
554	-2	0	-1	-0.94444	-0.8	-1.125	
555	-1.5	-0.5	0	0.388889	-0.9	2	
556	1.5	2.5	0.5	1.166667	1.3	1	
557	0.5	1.5	0	0.5	0.7	0.25	
558	-2.5	0.5	-2.5	0.055556	-0.7	1	
559	-2	-0.5	-1	-0.11111	-0.8	0.75	
560	-0.5	0	-0.5	0.166667	-0.1	0.5	
561	1.5	0	-1	1.5	1	2.125	
562	0.5	1	-1.5	-0.5	-0.1	-1	
563	1.5	0.5	1.5	-0.44444	0.7	-1.875	
564	0	-1	-0.5	-0.44444	-0.3	-0.625	
565	0.5	0	0	-0.33333	0	-0.75	
566	-0.5	0	1	0.277778	0.2	0.375	
567	-1.5	0	0.5	0	-0.1	0.125	
568	-1.5	-1	-2	-1.83333	-1.2	-2.625	
569	-0.5	-0.5	-1	-0.88889	-0.5	-1.375	
570	-1	0.5	0	-0.66667	-0.5	-0.875	
571	1	0	1	-0.38889	0.7	-1.75	
572	-0.5	1	1	0.722222	0.6	0.875	
573	1	0.5	0.5	0.944444	0.9	1	
574	-2	0	-1	-1.05556	-0.7	-1.5	
575	-2	-1	-1	-1.66667	-1.2	-2.25	
576	-1	0	-0.5	-0.88889	-0.2	-1.75	
577	2.5	2	1.5	0.944444	2	-0.375	
578	0	0	0.5	0.222222	0.1	0.375	

ID	STABLEfit	RELATIONf	HORNORfit	POFIT	relatedfi	achvmntfitscore	
579	1.5	1	2	0.333333	1.7	-1.375	
580	0.5	0	-0.5	-1.05556	0	-2.375	
581	4	3.5	3.5	1.166667	2.9	-1	
582	1	0.5	0.5	-0.88889	0	-2	
583	1	0.5	-1	-0.33333	-0.1	-0.625	
584	2	1.5	1	0.944444	2	-0.375	
585	0.5	2	1	-0.22222	1	-1.75	
586	-1	-0.5	-0.5	-0.77778	-0.2	-1.5	
587	0	0.5	-2	-0.05556	-0.6	0.625	
588	2	1	2.5	0.277778	1.6	-1.375	
589	-0.5	0	-1.5	-1.66667	-1.5	-1.875	
590	-4	1	-2	-0.77778	-1.5	0.125	
591	2	1.5	1	0.166667	1.4	-1.375	
592	-3	0	-1.5	-1.66667	-1.4	-2	
593	-0.5	-1	-1	-0.94444	-0.7	-1.25	
594	1	1	-0.5	0.388889	0.5	0.25	
595	-0.5	-0.5	-1	-1.33333	-1.3	-1.375	
596	0	0	-0.5	0.777778	0.3	1.375	
597	1	2.5	0.5	0.111111	1.3	-1.375	
598	-1	0	0	-0.11111	0.3	-0.625	
599	-2.5	0	-2	-2	-1.3	-2.875	
600	-3.5	0.5	0.5	-1.38889	-1.7	-1	
601	-1	-1	0	-0.77778	-0.9	-0.625	
602	1.5	2	-2.5	-0.94444	0.4	-2.625	
603	4	3.5	3	0.666667	2.6	-1.75	
604	-1.5	-0.5	-1.5	-1.44444	-1.2	-1.75	
605	-3	0.5	0.5	-0.94444	-1.2	-0.625	
606	2	1	-0.5	0	0.7	-0.875	
607	1	2	0.5	-0.77778	0.6	-2.5	
608	1	0.5	-1	-0.44444	-0.2	-0.75	
609	0.5	1.5	0.5	-0.22222	0.7	-1.375	
610	-1.5	-0.5	-1.5	-1.44444	-1.2	-1.75	
611	1.5	1	0.5	0.333333	0.9	-0.375	
612	1.5	1	-0.5	-0.11111	0.6	-1	

ID	STABLEfit	RELATIONf	HORNORfit	POFIT	relatedfi	achvmntfitscore	
613	0.5	1.5	3	0.277778	1.4	-1.125	
614	-1.5	0	1.5	0.222222	-0.1	0.625	
615	-1	0.5	-1	-0.61111	-0.6	-0.625	
616	1	0.5	0.5	0.388889	0.8	-0.125	
617	-1	-1	1	0	-0.2	0.25	
618	-1	-1	-1	-0.94444	-0.7	-1.25	
619	1.5	0	0.5	0.611111	0.7	0.5	
620	-1	0	-0.5	-0.66667	-0.2	-1.25	
621	-1	0	0.5	-0.38889	-0.4	-0.375	
622	-0.5	0	0	-0.5	-0.4	-0.625	
623	0	-0.5	-3	-2.05556	-1.3	-3	
624	-1	1.5	0	-0.66667	0.5	-2.125	
625	-0.5	1	-0.5	0.166667	-0.6	1.125	
626	0	-1	-2	-0.33333	-0.8	0.25	
627	-1	0	-1	-1.22222	-0.8	-1.75	
628	0.5	0	-2.5	-0.66667	-0.3	-1.125	
629	-1	0	0.5	-1.16667	-0.3	-2.25	
630	-1	0	-2	-0.66667	-0.4	-1	
631	0	0	-2	-0.94444	-0.4	-1.625	
632	0	1	-2	-0.33333	0	-0.75	
633	0	0.5	-1	0.444444	0.3	0.625	
634	-0.5	0	-0.5	-0.88889	-0.6	-1.25	
635	-1	-1	-2	-2.11111	-1.6	-2.75	
636	1	2.5	0.5	0.888889	1	0.75	
637	-0.5	-0.5	0	-0.33333	-0.2	-0.5	
638	1	-2	0.5	0.166667	0.3	0	
639	0	-0.5	-1.5	-0.55556	-1.1	0.125	
640	0.5	-0.5	-1	-1.33333	0	-3	
641	2	-0.5	1.5	0.611111	0.8	0.375	
642	2	-1	-1	-1.11111	-0.4	-2	
643	-1	2	-0.5	-0.61111	-0.1	-1.25	
644	0.5	0	-2	-1.11111	-0.1	-2.375	
645	-1	1	0	0.333333	0.1	0.625	
646	0	2	0	0	0.2	-0.25	

ID	STABLEfit	RELATIONf	HORNORfit	POFIT	relatedfi	achvmntfitscore	
647	0	2	2	0.944444	0.8	1.125	
648	1.5	0	0.5	0.333333	0.7	-0.125	
649	1	1	0.5	0.722222	1	0.375	
650	0	1	2	-0.05556	0.6	-0.875	
651	-3	-1	-4	-2.22222	-2.2	-2.25	
652	-1	-1	-2	-1.38889	-1.4	-1.375	
653	1.5	0.5	1.5	0.888889	1.1	0.625	
654	1	1	0	0.555556	0.7	0.375	
655	1	0.5	1	-0.38889	0.6	-1.625	
656	0	0.5	-0.5	-1.33333	-0.9	-1.875	

NAME	TYPE	WIDTH	LABLE	VALUES
ID	Numeric	6		None
ORG	String	26	organization	None
ORGID	Numeric	2	org id	None
DISTRICT	Numeric	1	区	{1, 庐阳区}…
province	Numeric	7	省（市）	{1, anhui;2, shandong; 3, shaanxi;4 shanghai}
rank	Numeric	6	rank	{1, no title;2, junior;3, middle; 4, senior}
quotastat	Numeric	1	是否在编	{1, inquota;2, outquota;3 contract}
GENDER	Numeric	1	gender	{0, female;1, male}
MARRI	Numeric	1	marriage	{1, notmarried;2 married; 3 divorced; 4,other}
AGE	Numeric	2	年龄	None
agegrp	Numeric	6	年龄段	{1, -29;2, 30-39;3, 40-49; 4, 50-59; 5, 60-}
WORKYEAR	Numeric	2	卫生工作年限	None
post2	Numeric	3	专业重编	{1, 临床专科}...
WKYEARCH	Numeric	2	tentureinchc	None
FULLTIME	Numeric	1		None
mngment	Numeric	1	management post	None
MEDEDUCA	Numeric	1	education	{1, middle school;2,professional college;3,university graduate;4,postgrad;5,non}
WESTMED	String	3	westernmed	None
CTM	String	3	chinesemed	None
WCM	String	3	west&chin	None
PREVENT	String	3	prevention	None
NURSING	String	3	nurse	None
MEDLAB	String	3	medlaboratory	None
ORAL	String	3	oral	None
PHARM	String	3	pharmacy	None
GPAE	String	3	gp	None
REHAB	String	3	康复	None
HEALTHMA	String	3	health admin	None
OTHERAE	String	3	other	None
genjs_1	Numeric	3	job satisfaction	None
LEAVE_1	Numeric	8	turnover intention	None
INCM_1	Numeric	8	income supply	None
BENEFIT_1	Numeric	8	benefit supply	None
LRN_1	Numeric	8	learning supply	None

NAME	TYPE	WIDTH	LABLE	VALUES
CAREER_1	Numeric	8	career develp supply	None
PEERRELA_	Numeric	3	peer relation spl	None
RESPECTS_	Numeric	3	community respect sp	None
SECURE_1	Numeric	3	job security spl	None
PARTIRES_	Numeric	3	participation in dec	None
HONOR_1	Numeric	3	honor spl	None
INCOME5	Numeric	6	income need	None
BENEFIT5	Numeric	6	benefit need	None
LEARN5	Numeric	6	learning	None
CAREER5	Numeric	6	career devlp need	None
PEERRELAT	Numeric	6	peer relation need	None
RESPECT5	Numeric	6	community respect ne	None
SECURE5	Numeric	6	job secure need	None
PARTICI5	Numeric	6	particpt in decisonm	None
HORNOR5	Numeric	6	hornor need	None
CAREERfit	Numeric	6		None
INCOMEfit	Numeric	6		None
LEARNfit	Numeric	6		None
BENEFITfi	Numeric	6		None
PARTfit	Numeric	6		None
RESPECTfi	Numeric	6		None
STABLEfit	Numeric	6		None
RELATIONf	Numeric	6		None
HORNORfit	Numeric	6		None
POFIT	Numeric	6		None
relatedfi	Numeric	6	fit score of related	None
achvmntfi	Numeric	6	fit score of achieve	None
